# An insight into the runs of homozygosity distribution and breed differentiation in Mangalitsa pigs

**DOI:** 10.3389/fgene.2022.909986

**Published:** 2022-10-18

**Authors:** Sowah Addo, Lisa Jung

**Affiliations:** Animal Breeding Section, University of Kassel, Witzenhausen, Germany

**Keywords:** Mangalitsa pig, ROH islands, genetic diversity, selection signatures, runs of homozygosity

## Abstract

Mangalitsa pigs exhibit three distinct coat color patterns based on which they are described as Red, Blond, and Swallow-bellied. The current study investigated genome-wide diversity and selection signatures in the three breeds using fixation index, runs of homozygosity and population structure analyses. The analyses were originally based on quality-controlled data on 77 Mangalitsa animals from Germany, including 23 Blond, 30 Swallow-bellied and 24 Red Mangalitsa genotyped with a customized version of the ProcineSNP60 v2 Genotyping Bead Chip. Also, 20 Hungarian Mangalitsa genotypes were included as outgroup data for comparison. Estimates of observed heterozygosity were 0.27, 0.28, and 0.29, and inbreeding coefficients estimated based on runs of homozygosity were 24.11%, 20.82%, and 16.34% for Blond, Swallow-bellied and Red Mangalitsa, respectively. ROH islands were detected in all breeds, however, none of these were shared amongst them. The KIF16B gene previously reported to play a role in synaptic signaling was found in a ROH island (SSC17: 16–26) in Swallow-bellied Mangalitsa. The same gene was found to harbor a significantly differentiated SNP (MARC0032380) while contrasting either Blond or Red to Swallow-belied Mangalitsa. In the Red Mangalitsa, some ROH islands were associated with genes that play a role in meat quality traits, i.e., ABCA12, VIL1, PLSCR5, and USP37. Our population structure analysis highlighted a separation of the three breeds, but also showed the closest relatedness between Red and Blond Mangalitsa pigs. Findings of this study improve our understanding of the diversity in the three breeds of Mangalitsa pigs.

## Introduction

Domestication and selection events can lead to both favorable and unfavorable allele reconstitution in animal species. Following its creation by domestication of wild pigs (*Sus scrofa fer* *us*) in the 19th century, Sumadija pigs of Serbia were reared under favorable conditions that made them lose their original form to become one of the progenitors of Mangalitsa pigs (Jukes 2017). Although originally from Serbia, Mangalitsa was systematically developed in Hungary at a time when market demand for good quality fat, bacon and less fibrous meat necessitated the crossing of small Hungarian sows such as Alfoldi, Bakony and Szalonta with Serbia’s improved Sumadija (Egerszegi et al., 2003; Jukes 2017). The product of such crosses was the Blond Mangalitsa, which was subsequently crossed either with Black Syrmian to develop Swallow-bellied Mangalitsa or with Szalonta to develop Red Mangalitsa (Zsolnai et al., 2013; Jukes 2017). Thus, three types of Mangalitsa pigs exist and these exhibit varying phenotypes, particularly coat color variation. In spite of the differences, Mangalitsa pigs are broadly described as fat-type, curly-haired pigs with strong motherliness and adaptability, but low reproductive performance (Egerszegi et al., 2003). They have geographical predominance in Hungary but are also distributed across Serbia, Romania, Austria, Croatia, Germany and Switzerland.

The three breeds of Mangalitsa are usually managed with restricted gene flow amongst them. Zsolnai et al. (2006) investigated the genetic relationships between the breeds using 10 microsatellite markers, and proposed a rejection of the hypothesis that Mangalitsa individual form just one unpartitioned population. They also found Blond and Swallow-bellied Mangalitsa to be genetically closer to each other than to Red Mangalitsa as did Marincs et al. (2013) who based their analysis on mitochondrial DNA D-loop sequences. Meanwhile, in a separate study, a conclusion has been drawn that mitochondrial DNA D-loop polymorphism could not distinguish between the three breeds (Molnár et al., 2013). Marincs et al. (2013) also unraveled the presence of both common European and Mangalitsa-specific mitochondrial DNA D-loop haplotypes in a Hungarian population and concluded that these pigs may have originated either by introgression of common European bloodline into the Mangalitsa breed or by isolation of some Mangalitsa ancestor species. Furthermore, Frank et al. (2017), for the first time found the mitogenomes of some Mangalitsa animals to be highly related to the Croatian Turopolje breed, which they attributed to either common origin of maternal lineages or admixture events. A comparative study of whole genome sequences of the three breeds of Mangalitsa and a Duroc pig highlighted on one hand, 52 Mangalitsa-specific genes involved in lipid metabolic processes, and on the other hand, several exonic polymorphisms unique to each of the breeds (Molnár et al., 2014). More recently, an investigation into the genetic basis of different colorations in Mangalitsa pigs revealed a display of signature of divergent directional selection in the solute carrier family 45-member 2 (SLC45A2) gene for the comparison of Red and Blond Mangalitsa breeds (Bâlteanu et al., 2021).

In spite of their genetic differentiation, there was no mentioning of breed type in a number of studies involving Mangalitsa pigs, and it was not immediately clear if animals of different coat colors where used (García et al., 2006; Wilkinson et al., 2013; Herrero-Medrano et al., 2014; Manunza et al., 2016; Schachler et al., 2020). In one of such studies, the mean genomic inbreeding coefficient estimated for 20 Hungarian Mangalitsa animals based on runs of homozygosity was high (0.22) for which reason the authors advocated for special conservation interventions to be put in place (Yang et al., 2017). Gorssen et al. (2021) recently presented a repository of ROH island for several breeds of eight animal species among which the Hungarian Mangalitsa pigs used in Yang et al. (2017) were featured. The repository shows high incidence of ROH occurring on chromosomes 11, 13, 14, and 17 in the Mangalitsa pigs. Bâlteanu et al. (2019) found runs of homozygosity based inbreeding coefficient ranging from 0.09 to 0.14 in different populations of Mangalitsa pigs and argued that the pattern of homozygosity in these local breeds is comparable to those of the majority of cosmopolitan breeds. Albeit, they found a clear indication of strong and recent inbreeding in Romanian and Hungarian Red Mangalitsa pigs which was attributed to mating of related individuals and a reduction in population size. In Germany, Managalitsa pigs are referred to as “Wollschweine”, and an attempt to preserve their genetics is evidenced by the naming of Mangalitsa as breed of the year 1999 (Flegler 1999). Nowadays, there is growing interest of German breeders in breeding all three types of Mangalitsa, while the Society for the Conservation of Old and Endangered Livestock Breeds (GEH e.V.) is rapidly promoting the establishment of a herd book for the Mangalitsa pigs in Germany.

The availability of different Mangalitsa genotypes that exhibit phenotypic variability offers new possibilities of studying signatures of selection that may have played a role in the development of the three breeds. Besides, the ROH landscape of these breeds can be conveniently compared to those of other breeds published in a novel ROH repository to improve our understanding of selective sweeps in pigs. Therefore, the present study aimed at 1) investigating genome-wide relatedness among Blond, Red and Swallow-belied Mangalitsa pigs, 2) identifying genomic regions with high level of inbreeding termed ROH islands, and 3) finding candidate genes that may be associated with significantly differentiated genomic regions in the breeds.

## Materials and methods

### Animal description

**FIGURE 1:**
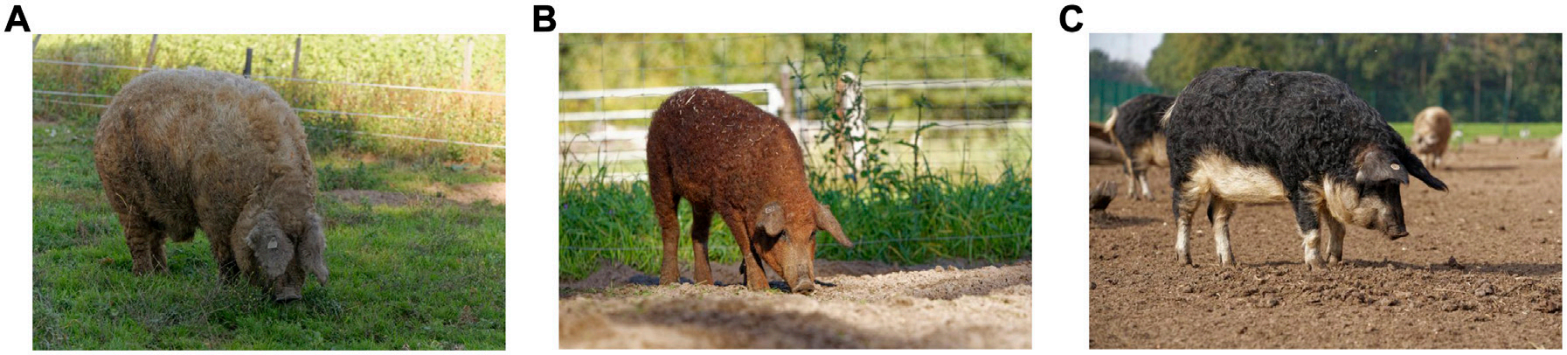
The three different breeds of Mangalitsa pigs including Blond **(A)**, Red **(B)** and Swallow-bellied **(C)**. Pictures were provided by Rudi Gosmann.

In this study, 109 animals belonging to the three main breeds of Mangalitsa pigs were initially considered. These include Blond, Red and Swallow-bellied Mangalitsa, hereafter referred to as BM, RM, and SM, respectively. The animals were from individual breeders and animal parks, and each had a registration number provided by the GEH e.V. in Germany. Animals were distinguishable, predominantly by their coat-color variation (Figure 1). BM individuals have a general grey to yellow to yellowish red coat-color with the head and leg regions often almost black (Figure 1A). RM tend to have darker or lighter shades of reddish-brown (Figure 1B) while SM have a blackish-brown coloration at the back and flanks and yellow, white or silvery grey at the underside, belly and cheek areas (Figure 1C). No extensive pedigree data were available at the time of sample collection, and a number of animals were said to have been produced by crossing two of the three breeds. Due to issues of misidentification and uncertainty, these animals, totaling 17, were labeled as mixed breed (MM) and removed from the analyses. The remaining animals included 34 SM, 29 RM, and 29 BM pigs. Additionally, we included data on 20 Hungarian Mangalitsa pigs (HUMA) of unspecified breed type from previous studies (Yang et al., 2017) for comparison.

### Genotyping and quality control

The collection of hair samples, DNA extraction and genotyping followed standard procedures and were performed in two batches, in 2018 and 2020. Animals were genotyped with customized versions of the ProcineSNP60 v2 Genotyping Bead Chip. SNP markers common to both batches were extracted and mapped to the *Sus scrofa* 11.1 genome assembly. A number of quality control procedures were conducted in PLINK v 1.9 (Chang et al., 2015) depending on the desired type of analysis. Unmapped and non-autosomal SNPs were broadly removed as were 2 (SM) and 1 (RM) animals whose genomic relationship coefficient with other pairs exceeded 0.95. Individual and marker genotyping rate thresholds were both set to 90%, and SNPs with minor allele frequency (MAF) lower than 0.05 or that deviated from Hardy Weinberg Equilibrium (HWE: 10^−6^) were removed. Specifically, for ROH analyses, SNP call rate was set to 95% for easy comparison (Gorssen et al., 2021) and there was neither MAF nor HWE pruning as recommended in previous studies (Meyermans et al., 2020). MAF pruning was also not conducted on data destined for Fixation index (F_st_) analysis (Weir and Cockerham 1984) and the quality control steps were performed for each breed separately. Furthermore, a minor linkage disequilibrium (LD) pruning was applied to the dataset destined for population structure analysis by using the PLINK (Chang et al., 2015) command “-- indep-pairwise 50 25 0.5”. After all filtering steps, 23 BM, 24 RM, and 30 SM genotypes remained for further analyses.

### Diversity and population structure

Within-breed genetic diversity was investigated based on observed heterozygosity estimates calculated as a difference between the number of homozygous and non-missing genotypes, expressed as a proportion of non-missing genotypes. Also, the relationship between BM, RM, and SM was investigated using principal component analyses. LD-pruned SNPs totaling 10,323 were used to compute Plink-based (Chang et al., 2015) eigenvectors for the first 20 components for each individual. Subsequently, eigenvectors of the first (PC1) and second (PC2) principal components were visualized in R (R Core Team 2020) using breed as color code. This investigation was further expanded to include the 20 HUMA outgroup data and the analysis was based on 8,624 quality-controlled SNPs common to all animals. The population structure was also studied using ADMIXRURE (Alexander et al., 2009). For this, a 5-fold cross-validation procedure was performed for a range of k between 1 and 20, and the k with the lowest cross-validation error was considered as the optimal number of clusters for the data. Cluster assignments ranging from k = 2 to k = 9 were visualized using Pophelper (Francis 2017).

To detect genetic differentiation over time, the F_st_ (Weir and Cockerham 1984) between breeds was calculated for all loci using PLINK (Chang et al., 2015). Subsequently, an empirical *p*-value for each SNP was estimated following a z-score calculated from the distribution of F_st_ values. The F_st_ values were visualized using Manhattan plots implemented in the qqman r package (Turner 2018), and the top 0.1% were suggested as signatures of selection.

### ROH calling and analysis

The number of SNPs available for ROH analyses after quality control was 36,617 (BM), 38,085 (RM), and 36,964 (SM). ROH calling was performed using an R-script developed for standardized breed-by-breed quality control and analysis (Gorssen et al., 2021), which is available at Open Science Framework (https://doi.org/10.17605/OSF.IO/XJTKV). Default parameter settings as described in Gorssen et al. (2021) were followed in defining ROH, ROH incidence and ROH islands. Briefly, we considered a sliding window with minimal number of SNPs determined by an L-parameter (Purfield et al., 2012; Meyermans et al., 2020). Within this window, 1 SNP could be missing but no heterozygous SNP was allowed, and there was at least 1 SNP every 150 kb. Additionally, the maximal gap between two consecutive homozygous SNPs was set to 1,000 kb.

Genomic inbreeding coefficient (F_ROH_) was calculated for each animal as the total length of all ROH in the genome of an individual expressed as a proportion of the length of autosomal genome coverage expressed by SNPs in the analysis (McQuillan et al., 2008). F_ROH_ was calculated considering all ROH (F_ROH_all_) in an individual but also for different ROH length categories including 1–2 Mb (F_ROH1-2_), 2–4 Mb (F_ROH2-4_), 4–8 Mb (F_ROH4-8_), 8–16 Mb (F_ROH8-16_) and >16 Mb (F_ROH16_).

For a given breed, the percentage of individuals with a specific SNP in ROH was defined as ROH incidence. From the distribution of ROH incidences, a threshold (*p*-value) was calculated based on standard normal z-scores. ROH islands were defined as the top 0.1% of SNPs with a *p*-value higher than 0.999 using a z-score table for ROH incidence (Purfield et al., 2017; Gorssen et al., 2020). Finally, a ROH must be present in at least 30% of the population to be part of a ROH island. ROH incidences and thresholds were visualized for each breed *via* Manhattan plots using the qqman package (Turner 2018).

### Identification of candidate gene

Significant genome variants from both F_st_ and ROH analyses were annotated to the *Sus scrofa* genome version 11.1 reference assembly, and the “sscrofa_gene_ensembl” dataset was explored using biomaRt v. 2.50.3 (Durinck et al., 2009). Furthermore, genes within 100 kb distance on either side of these variants were identified as candidate genes that may have played a role in the development of the breeds.

## Results

### Population structure and fixation index

**FIGURE 2:**
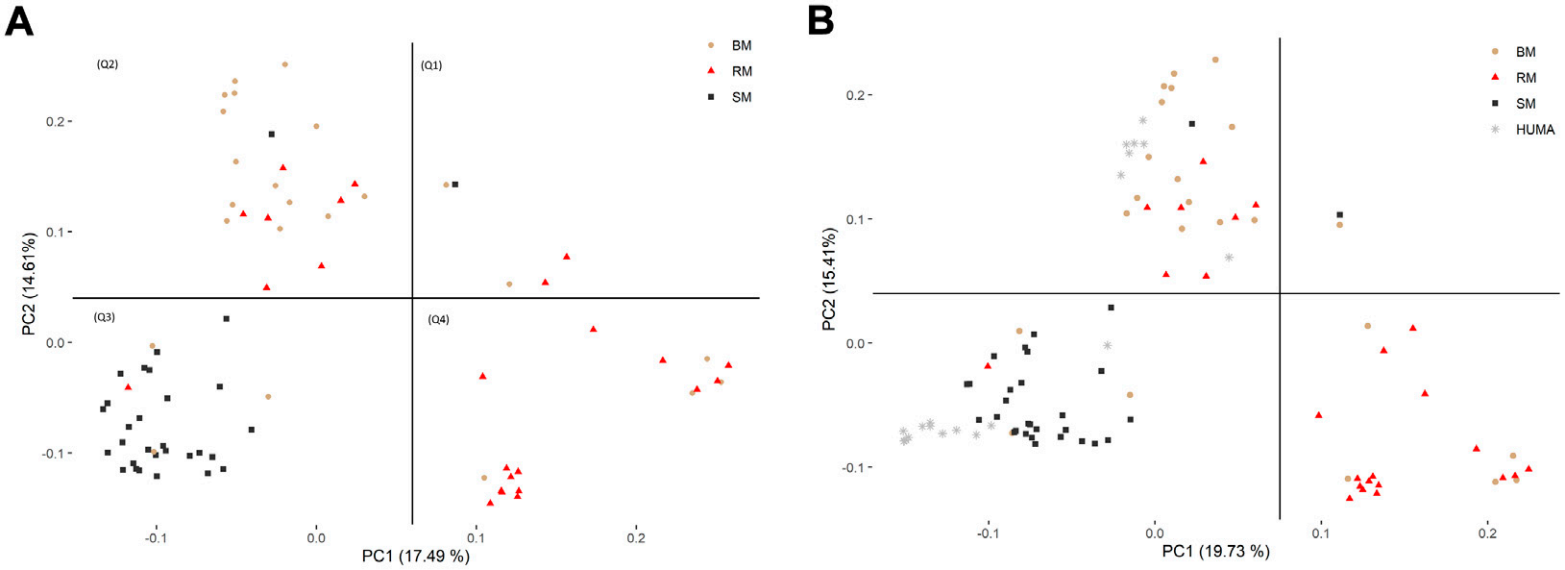
Distinguishing BM, RM and SM through principal component analysis based on 10,323 SNP markers **(A)**. The inclusion of HUMA in the analysis **(B)** was based on 8,624 SNP markers mapped to the Sus scrofa 10.2 genome assembly. The plotted area is divided into quadrants based on the occurrence of clusters and breeds are distinguished by color and shape.

**FIGURE 3:**
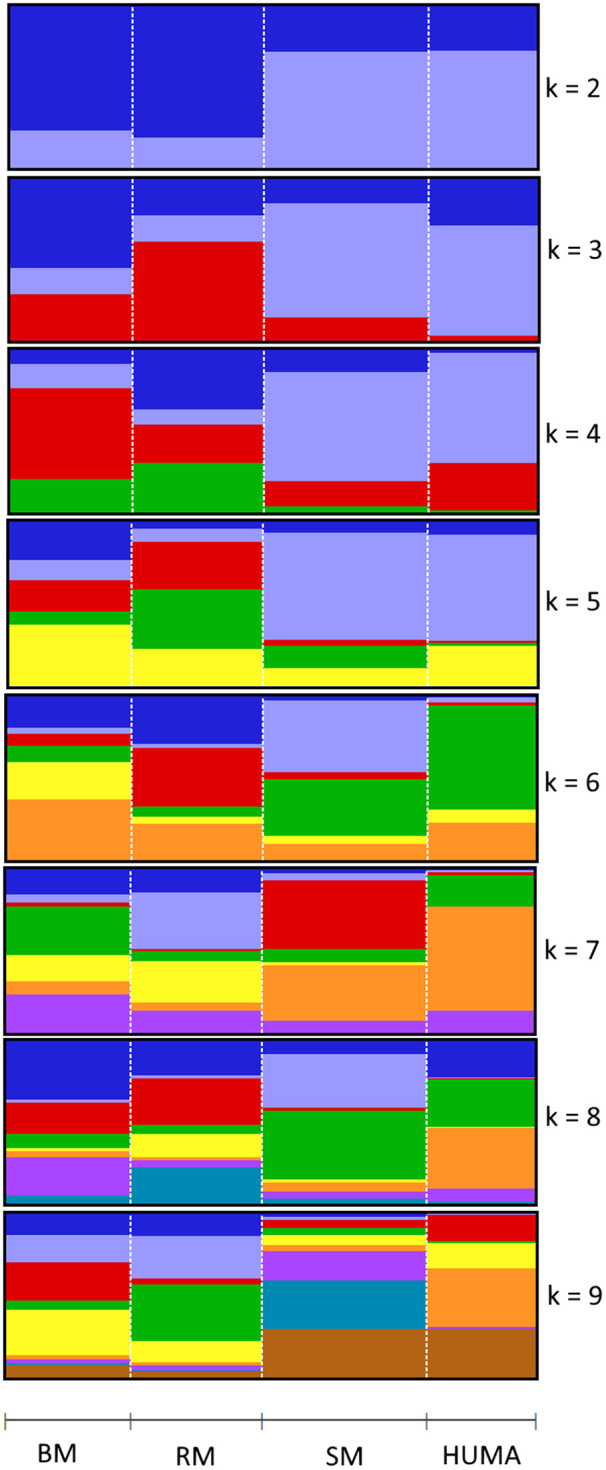
Admixture analysis of four Mangalitsa pigs populations (BM, RM, SM and HUMA) with graphs representing cluster levels 2 through 9. The optimal cluster level was found at k = 6.

In the principal component analysis, PC1 and PC2 together explained 32,1% of variation in the three breeds (Figure 2A). There was a clustering along the lines of breeds, but notably, clusters were not well defined. In the third quadrant (Q3) of the plotted area, SM was predominantly separated from RM and BM by PC1 and PC2, respectively. In the second quadrant, what seemed to be a BM cluster harbored several genotypes of RM. The RM breed formed a cluster in the fourth quadrant with the highest level of dispersion. This latter cluster also harbored genotypes of BM. By including HUMA genotypes in the analysis (Figure 2B), 35.14% of the variation in the data was explained. Majority of the HUMA clustered close to SM, and a few were in the cluster of BM. The ADMIXTURE analysis for low levels of k, especially, k = 2 showed a high degree of similarity of genetic background between BM and RM on one hand, and on the other hand, similarity between SM and HUMA (Figure 3). However, there was no complete separation of all breeds as demonstrated by traces of admixture at all cluster levels including k = 6, which produced the lowest cross-validation error estimate (Supplementary Figure S1). Additionally, genetic background was highly heterogeneous in all breeds.

**FIGURE 4:**
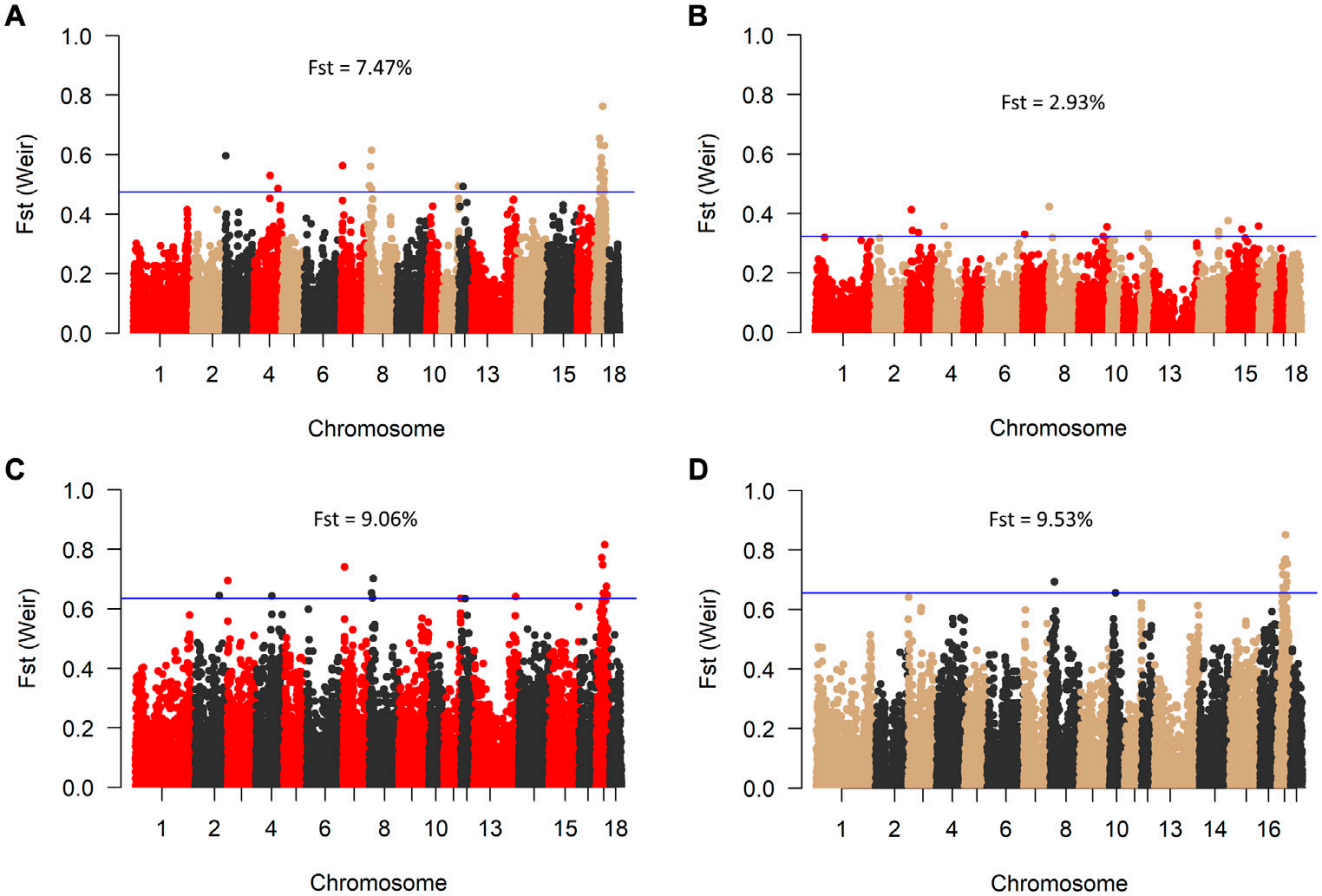
Manhattan plot of genome-wide Fst values between BM, RM and SM **(A)**; RM and BM **(B)**; RM and SM **(C)**, and BM and SM **(D)**. The blue line indicates genome-wide significant threshold above which SNPs were considered significant for candidate gene identification. Mean Fst values for each comparison is specified in percentage.

Consistent with the principal component analysis, the lowest mean F_st_ (0.029) was found between RM and BM, while these two breeds were relatively distant from SM (Figure 4). By comparing all three breeds in a single F_st_ analysis, 30 genome-wide significant variants were detected across SSC 3, 4, 7, 8, 11, 12, and 17. Most of the significant variants (67%) were located on SSC17, which also had the topmost SNP (DRGA0016741) at position 38908507. The restriction of the analysis to a pairwise comparison revealed 14 significant variants between RM and BM with none occurring on chromosome 17. A number of significant variants were common to the comparison between RM and SM and between BM and SM. Five of these variants are depicted in Table 1 with superscript letters a—e. Additionally, Table 1 provides candidate genes located within 100 kb on either side of the F_st_-based significant variants.

**TABLE 1:** Summary information of significant SNPs and candidate genes from F_st_ comparison of three Mangalitsa pig breeds (BM, SM and RM). Superscripts a-e denote common SNPs across pairwise comparisons.

Breeds	CHR	SNP	Position	F_ST_	Candidate genes
BMSM	17	DRGA0016741^a^	38908507	0.85	CEP250, ERGIC3, SPAG4, CPNE1, NFS1
	17	MARC0026961	39771108	0.77	DLGAP4, TGIF2, MYL9
	17	MARC0017379	33921649	0.76	SIRPB2, NSFL1C, SDCBP2, SNPH
	17	ALGA0095426^b^	46764111	0.75	OSER1, GDAP1L1, FITM2, R3HDML, HNF4A
	17	MARC0032380^c^	25017561	0.74	KIF16B
	17	H3GA0048218	26643149	0.72	ZNF133, DZANK1, POLR3F, RBBP9
	17	ALGA0094584^d^	33341575	0.72	STK35
	17	H3GA0049036	41739085	0.72	SLC32A1, ACTR5, PPP1R16B
	8	ALGA0046856^e^	20576000	0.69	STIM2
	17	ASGA0077154	45575325	0.69	PTPRT
	17	ALGA0094114	27748357	0.68	SLC24A3, RIN2
	17	ALGA0095121	41180101	0.67	RPRD1B, TGM2, KIAA1755
	17	ASGA0076251	30236910	0.66	THBD, CD93
	10	H3GA0029711	26091506	0.66	ERCC6L2
RMSM	17	DRGA0016741^a^	38908507	0.82	CEP250, ERGIC3, SPAG4, CPNE1, NFS1
	17	MARC0032380^c^	25017561	0.77	KIF16B
	7	ALGA0038333	7188826	0.74	TFAP2A
	8	ALGA0046856^e^	20576000	0.70	STIM2
	3	H3GA0008443	2734718	0.70	SDK1
	17	ALGA0095426^b^	46764111	0.68	OSER1, GDAP1L1, FITM2, R3HDML, HNF4A
	17	ALGA0094584^d^	33341575	0.65	STK35
	17	ASGA0077190	46189837	0.65	SRSF6, SGK2, IFT52, MYBL2
	17	M1GA0022187	49729457	0.65	NCOA3, SULF2
	2	H3GA0007369	113554653	0.64	FBXL17
	4	ALGA0026041	75779884	0.64	PLAG1, MOS, RPS20, LYN
	13	M1GA0017756	193064698	0.64	GRIK1
	8	ALGA0046640	16400498	0.64	KCNIP4
RMBM	8	M1GA0011680	2332002	0.42	ADRA2C
	3	ALGA0017963	20715185	0.41	HS3ST4
	4	MARC0024274	39449105	0.36	TSPYL5, CPQ
	15	MARC0036536	137538236	0.36	RBM44, RAMP1, SCLY
	9	ALGA0055521	130209873	0.35	RPS6KC1
	15	ALGA0107554	60110316	0.35	FMNL2
	3	ALGA0018104	25852288	0.34	GPR139, GPRC5B, IQCK
	3	ALGA0019015	53054275	0.34	CREG2, RNF149, CNOT11, TBC1D8
	12	MARC0039239	35751427	0.33	DHX40, CLTC
	7	ASGA0031021	8122178	0.33	NEDD9, TMEM170B

### Within-breed diversity

**FIGURE 5:**
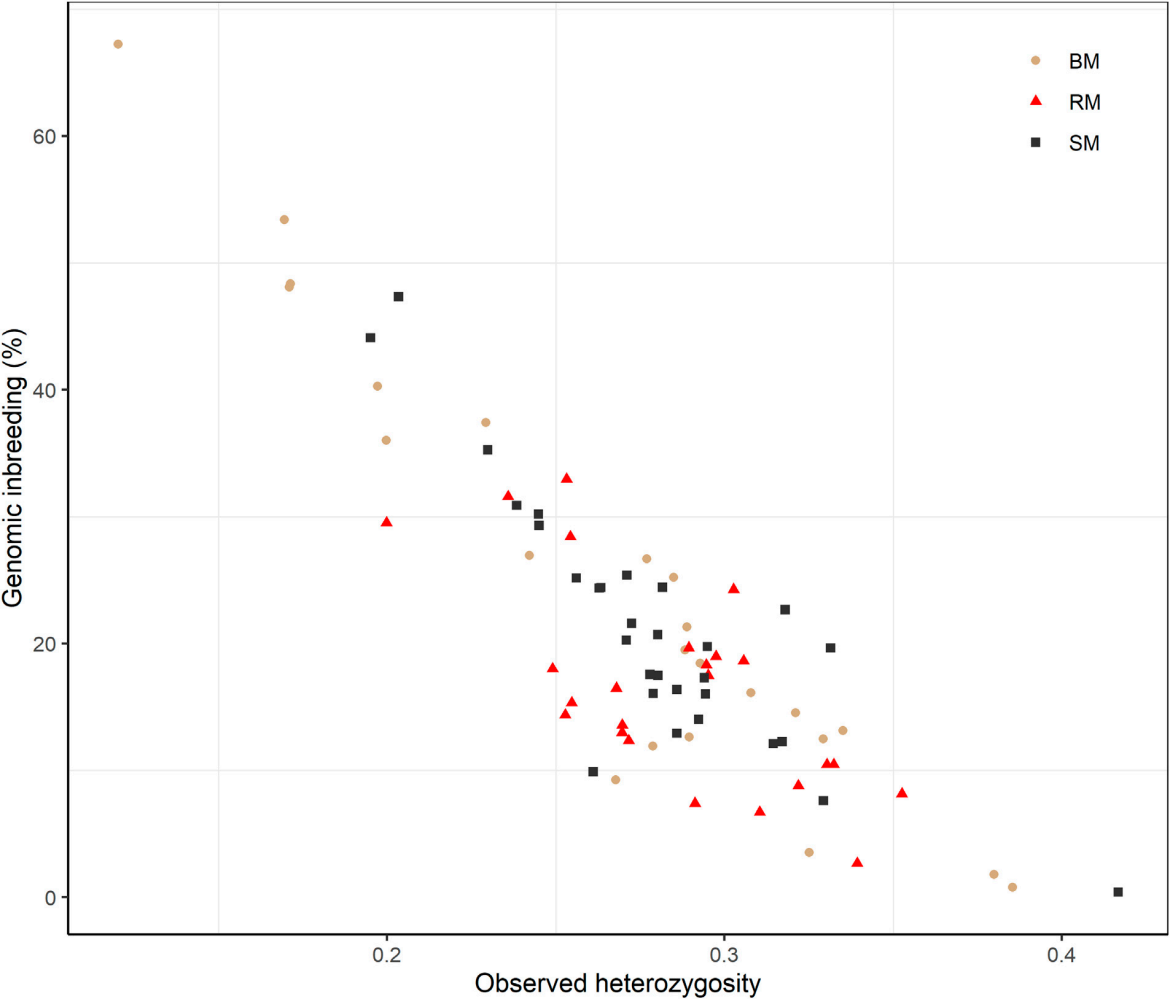
Scatterplot of the correlation between observed heterozygosity and genomic inbreeding (FROH) for Blond, Red and Swallow-bellied Mangalitsa pigs. The Pearson correlation coefficient was significant across breeds (r = 0.88; p-value < 2.2e-16).

Average Ho estimates were 0.27, 0.28 and 0.29 for BM, SM and RM, respectively. The heterozygosity estimates correlated significantly and negatively with total genomic inbreeding (r = -0.88; *p*-value < 2.2e-16) as shown in Figure 5. Across breeds, Ho ranged from 0.12 at the highest level of F_ROH_ (67.25%) to 0.42 at the lowest inbreeding level (0.39%).

**FIGURE 6:**
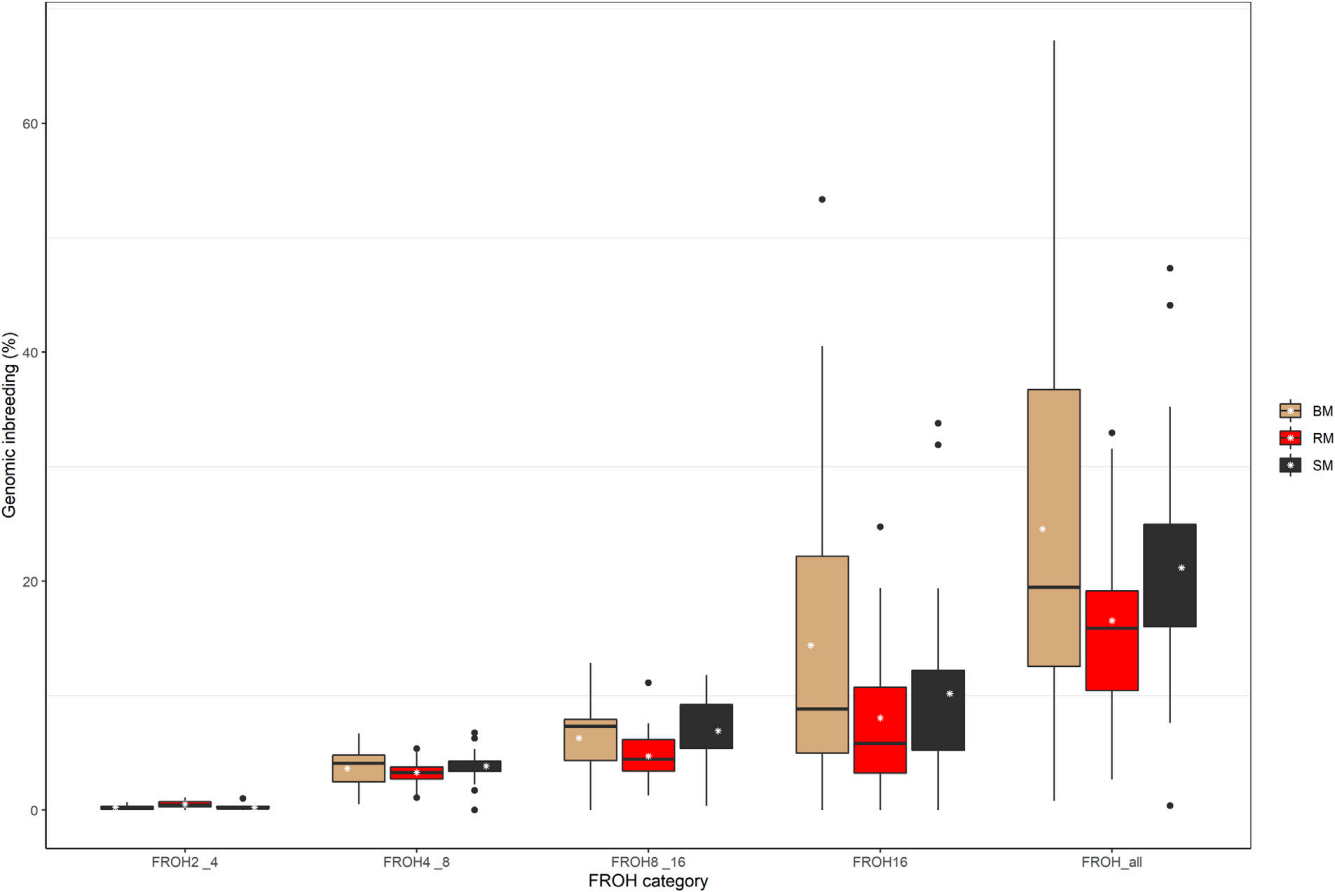
Boxplot showing the distribution of genomic inbreeding for various ROH length categories in Blond, Red and Swallow-bellied Mangalitsa pigs. The white stars represent estimate of average inbreeding.

The computed values of F_ROH_ for various ROH length considerations are presented with respect to breed in Figure 6. Considering all ROH, average inbreeding was highest in BM (24.11%) and lowest in RM (16.34%). This is also true for other ROH length considerations. Larger ROH segments played a major role in autozygosity in all breeds, and ROH segments below 2 Mb in size were completely absent in all animals. In general, fewer number of ROH segments were found in RM than in BM and SM (Table 2). Although BM and SM had about the same average number of ROH segments, the former had the highest sum of ROH (543626.6 kb) per individual. Across all breeds, 2715 ROH segments were found in this study.

**TABLE 2:** The average and total number of ROHs, and average sum of ROH length in BM, RM, SM and across all three breeds (ALL). Minimum and maximum values are provided in brackets.

Breed	Mean Nr. ROH (min∔max)	Total Nr. ROH	Mean ∑ROH in kb (min∔max)
BM	37.00 (3–64)	851	543626.6 (17568–1487860)
RM	30.83 (9–50)	740	368431.3 (59347–733062)
SM	37.47 (1–56)	1,124	469469.6 (8,582–1049150)
ALL	35.26 (1–64)	2,715	460128.0 (8,582–1487860)

**FIGURE 7:**
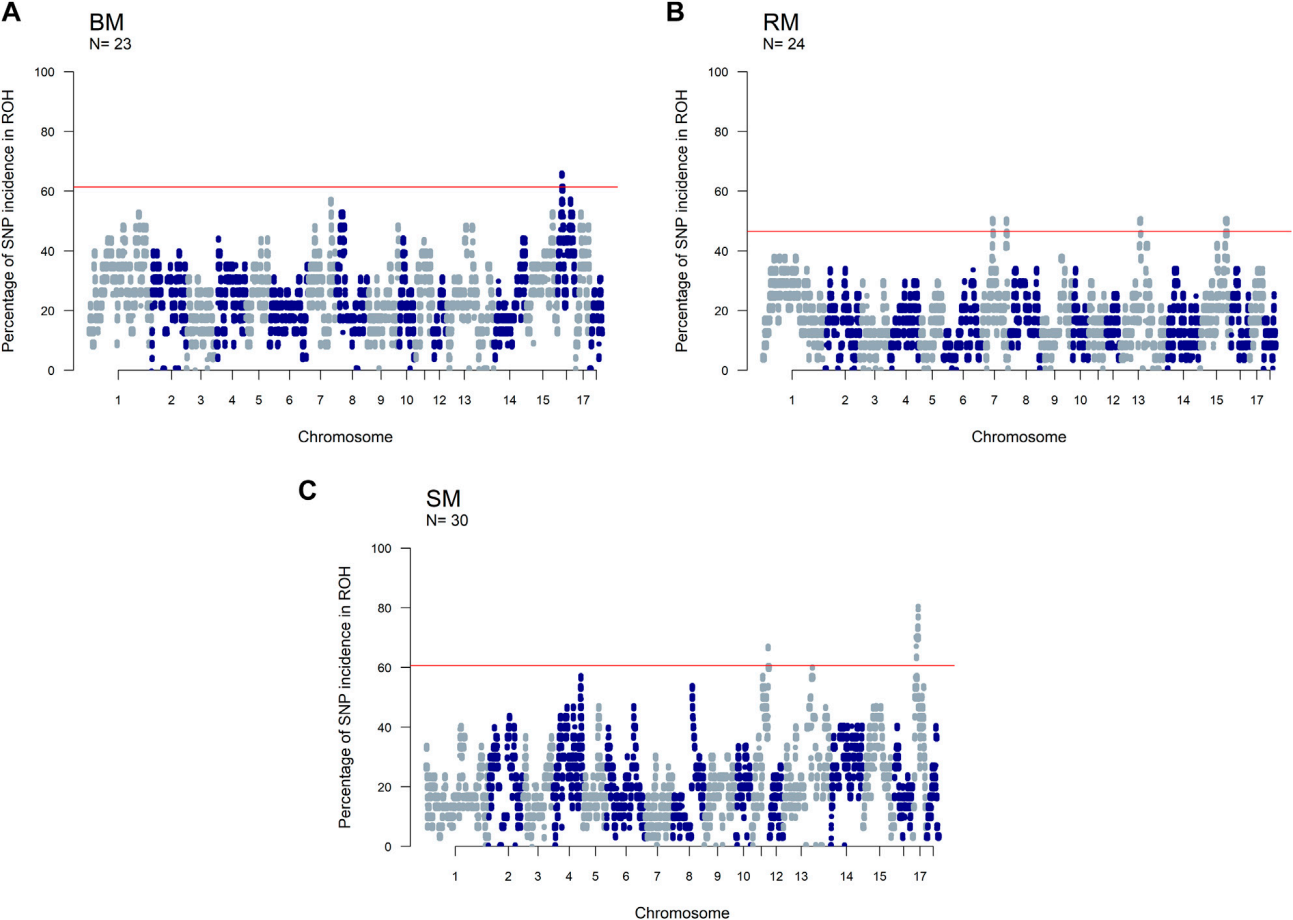
Manhattan plots of the incidence of SNPs in ROH for three Mangalitsa pig breeds: Blond **(A)**, Red **(B)** and Swallow-bellied **(C)**. The red line corresponds to a population specific p-value above which SNPs are considered to be in ROH islands.

The incidence of ROH and ROH islands varied across breeds (Figure 7). BM and SM had higher baseline ROH levels with ROH islands detected on SSC16 in BM, and on SSC11 and SSC17 in SM. The most significant variant in ROH island was found in 24 out of 30 SM animals on SSC17. In RM, ROH incidence levels were generally low, however, ROH islands were found on SSC7, SSC13 and SSC15. ROH incidence plots per chromosome show seemingly similar patterns across breeds (Supplementary Figures S2–S4). Furthermore, candidate genes proximal to ROH islands are presented in Table 3. Significant variants with no genes found within 100 kb distance on either side were not included.

**TABLE 3:** Summary information of significant SNPs and candidate genes from ROH islands in three Mangalitsa pig breeds (BM, SM and RM).

Breed	CHR	SNP	Position	ROH (%)	Candidate genes
SM	17	MARC0086847	25118032	80.08	KIF16B
	17	H3GA0048106	23564839	73.89	MACROD2
	17	MARC0030901	24049827	73.54	MACROD2
	17	ALGA0093805	22510190	70.63	ESF1, NDUFAF5, SEL1L2
	17	MARC0109531	20937630	70.63	BTBD3
	17	H3GA0048183	26170282	70.40	RRBP1, BANF2
	17	H3GA0047989	17680444	63.92	PLCB1
	11	H3GA0032842	77609850	60.66	ARHGEF7
	11	DRGA0011540	74100734	60.65	EFNB2
BM	16	MARC0086770	20525463	65.77	RAI14, BRIX1, RAD1, AGXT2, PRLR
	16	H3GA0046210	21372379	61.39	IL7R, CAPSL, LMBRD2, SKP2
	16	ALGA0115908	22998327	60.99	WDR70, GDNF
RM	15	DRGA0015508	120520780	50.81	CATIP, SLC11A1, CTDSP1, VIL1, USP37, CNOT9
	15	CASI0006663	117450632	50.81	ABCA12
	15	MARC0003725	119092405	50.81	TNP1
	15	ALGA0086843	114044775	50.80	ERBB4
	7	DIAS0001981	46539039	50.80	TMEM14A, GSTA1, GSTA4
	13	ASGA0058507	87695642	50.73	ZIC4, ZIC1
	13	H3GA0037098	89899499	50.72	WWTR1, COMMD2, ANKUB1
	7	ALGA0041512	47324213	50.71	CHRNA3, CHRNA5
	13	ALGA0071380	86992936	50.70	PLSCR5
	7	ASGA0033712	48095614	50.57	MORF4L1, CTSH, RASGRF1
	15	ASGA0070454	116910010	50.38	VWC2L
	15	DRGA0015443	113969520	49.67	ERBB4
	13	CASI0008610	90639691	46.65	TSC22D2
	7	DIAS0001022	110412608	46.65	SPATA7, PTPN21, ZC3H14, EML5,
	15	MARC0014079	115472905	46.50	IKZF2
	7	ALGA0044724	111026023	46.45	FOXN3
	7	ALGA0041861	49748787	46.44	CEMIP, MESD, TLNRD1

In the separate analysis, where SNP positions were based on *Sus scrofa* genome version 10.2, ROH islands were detected on SSC7 (113–117 Mb) and SSC15 (125–134 Mb) in RM, and on SSC11 (80–86 Mb) and SS17 (17–29 Mb) in SM (Supplementary Table S1). For BM, no SNP reached a *p*-value > 0.999 for ROH due to the breed’s high level of inbreeding. Therefore, we set a maximal threshold of 55% (*p*-value ⩾ 0.998) for ROH island detection. Hence, ROH islands were found on SSC7 (104–106 Mb), SSC8 (29–31 Mb) and SSC16 (21–24 Mb; 60–62 Mb) in BM.

## Discussion

The Mangalitsa pig with its dense curly hair and forward falling ears is considered precious, offering advantages such as resistance to challenging weather conditions and diseases (Egerszegi et al., 2003). Also, it is not very demanding on housing conditions and feeding. Therefore, breeders in Balkan countries, Austria, Switzerland, and Germany aim to conserve the original Mangalitsa without deleterious effects of inbreeding (Egerszegi et al., 2003). There is a growing interest in the exploration of the genome resource of Mangalitsa pigs, relying on an array of genetic markers including microsatellites (Zsolnai et al., 2006; Druml et al., 2012; Kharzinova and Zinovieva 2020), mitochondrial DNA D-loop sequences (Marincs et al., 2013; Molnár et al., 2013; Frank et al., 2017), SNPs (Bâlteanu et al., 2019; Bâlteanu et al., 2021; Zorc et al., 2022) and whole genome sequences (Molnár et al., 2014). Previous studies have attempted to discriminate between Mangalitsa pigs of different colors, highlighting a rejection of the hypothesis that Mangalitsa individuals belong to one indistinguishable group (Zsolnai et al., 2006). The current study did not only investigate genetic differentiation between the breeds, but also probed the occurrence of candidate genes in the vicinity of significantly differentiated marker loci. Additionally, we listed genes that are proximal to genomic regions displaying high levels of autozygosity. Whether or not these genes are differentially expressed in the studied breeds remains a subject for discussion.

### Population structure and diversity

The high degree of clustering of individual Mangalitsa pigs by breed in this study (Figure 2) is a notable observation echoing the findings of previous studies. Based on 10 microsatellite markers, Zsolnai et al. (2006) separated the three breeds *via* genetic distance and Structure analyses. Bâlteanu et al. (2019) also reported some degree of differentiation between the three breeds and other pig types through Admixture and principal component analysis using the Porcine SNP60 Beadchip. The persistency of traces of common genetic background across all cluster levels in our study reflects the developmental history of the breeds—RM and SM derived from BM. This is also consistent with previous studies that partly suggest that a present-day Mangalitsa population evolved by introgression of other European breeds and wild boars (Marincs et al., 2013). Explaining their observation of a close relationship between Mangalitsa and Turopolje pigs, Frank et al. (2017) mentioned a common wild boar, the Siska pig, assumed to be amongst the ancestors of both breeds. Another study suggests a possible geneflow from Slavonian Black and Pietrain pigs into both Mangalitsa and Turopolje pig populations, and reported lower levels of differentiation (from F_st_ = 0.05 to F_st_ = 0.09) between Mangalitsa and Slavonian Black (Druml et al., 2012). The closeness between Mangalitsa (SB) and Slavonian Black was confirmed by Zorc et al. (2022) who also showed potential gene flow between SB and Moravka pigs using both microsatellite and SNP data. What remains inconsistent across studies is the degree of differentiation among the breeds. We found in the current study, close connectedness between BM and RM than each of them to SM, which was corroborated by our F_st_ analysis, i.e., 0.029 (BMRM) vs. 0.091 (RMSM) or 0.095 (BMSM). On the contrary, Zsolnai et al. (2006) found BM and SM to be closer to each other (F_st_ = 0.064) than each of them to RM (F_st_ equals 0.094 and 0.099, respectively). Similarly, Marincs et al. (2013) found lower estimates of both F_st_ and Nei’s distance for BMSM than for either BMRM or RMSM. Their findings are consistent with the observation of a high degree of similarity of genetic background between BM and SM at low cluster levels in Bâlteanu et al. (2019). Differences between our findings and those of previous studies could stem from differences in breeding focus and management across populations since the genotypes in the previous study were of Hungarian and Romanian origin. It is therefore, not surprising that the 20 HUMA genotypes, which we later included in our analysis, formed clusters predominantly with SM, and with BM to a lesser extent. Thus, we suspect the HUMA outgroup genotypes to belong to the SM breed, however, this cannot be immediately confirmed. Albeit, we consider the high resemblance between BM and RM (our finding) symbolic of the crossing of BM with Szalonta to develop RM (Zsolnai et al., 2013; Jukes 2017). Worth mentioning is that Szalonta is already one of the progenitors of BM (Egerszegi et al., 2003; Jukes 2017).

The dispersion of RM genotypes in our analysis reveals either a comparatively high level of genetic diversity or population substructures in this breed. Consistently, RM had the highest heterozygosity estimate (0.29) in our study. Even so, for the same breed types, observed heterozygosity estimates in previous studies are slightly higher than in ours, i.e., 0.32–0.38 vs. 0.29 for RM, 0.31 vs. 0.27 for BM and 0.29 vs. 0.28 for SM (Bâlteanu et al., 2019). Higher heterozygosity values in Bâlteanu et al. (2019) were associated with relatively low levels of inbreeding. Thus, across the three breeds, average F_ROH_ ranged from 0.09 to 0.14 in Bâlteanu et al. (2019) compared to our range of values being 0.16 to 0.24. A lower SNP-based observed heterozygosity value of 0.26 with a corresponding F_ROH_ estimate of 0.29 was previously estimated for SM by Zorc et al. (2022). They found SM to be second (after Turopolje: F_ROH_ = 0.51), to have low level of genetic diversity amongst other local Balkan pig breeds. Contrary to previous finding (Bâlteanu et al., 2019) of RM exhibiting the strongest and most recent inbreeding, BM in our study had the highest level of autozygosity regardless of the age of inbreeding (Figure 6). Nevertheless, consanguinity increased in all three breeds in more recent generations, probably, about three generations ago as demonstrated by predominance of ROH larger than 16 Mb (Meszaros, 2015). More generally, Mangalitsa pigs are known to have undergone a serious demographic decline in the past (Egerszegi et al., 2003; Posta et al., 2015; Bâlteanu et al., 2019). For the outgroup HUMA breed, observed heterozygosity estimated by Gorssen et al. (2021) was 0.26 with a corresponding high level of F_ROH_ estimate of 0.41. Since Gorssen et al. (2021) mentioned Yang et al. (2017) as source of the HUMA dataset used in their ROH analysis, we compared the estimate of F_ROH_ obtained for HUMA in the two studies. Average F_ROH_ for the same individuals was almost halved (0.22) in Yang et al. (2017). Variability in ROH statistics across studies are a direct consequence of differences in ROH calling criteria (Addo 2020; Meyermans et al., 2020) In this study, we used an R-script identical to Gorssen et al. (2021) for ROH calling, making it possible to compare our results to those available at the Open Science Framework (https://doi.org/10.17605/OSF.IO/XJTKV). For suitability of ROH island comparison, we further performed a separate analysis of our data using a previous genome assembly (*Sus scrofa* 10.2 genome build) as was used in the previous study. Autozygosity across breeds in our population of Mangalitsa pigs (*n* = 77) was by far lower (∼0.21) than was found for the Hungarian population (*n* = 20). The HUMA genotypes were sampled between 2008 and 2010 while we sampled our animals about a decade later, between 2018 and 2020. It could be that breed conservation strategies are being effectively implemented to save these endangered animals in recent years. Moreover, Bâlteanu et al. (2019) argued that ROH statistics recorded in Mangalitsa pigs are comparable to those measured in most cosmopolitan breeds, adding the difficulty in predicting significant statistical differences due to high dispersion of F_ROH_ data in Mangalitsa pigs. Nevertheless, comparing F_ROH_ value of 0.14 (Bâlteanu et al., 2019) to that of 0.29 in SB, Zorc et al. (2022) emphasized recent increase in inbreeding due to severe reductions in census numbers.

The inverse relationship between inbreeding coefficients and heterozygosity was generally studied across breeds in the current study. Our finding of a strongly negative correlation (−0.88) between the two variables is not limited to this study. Slate et al. (2004) investigated the use of multi-locus heterozygosity as a robust surrogate for inbreeding coefficients for subsequent investigation of inbreeding depression, which is especially important for captive populations. Based on a limited number of microsatellite markers, not only Slate et al. (2004) but also other studies, e.g., Hedrick et al. (2001), Overall et al. (2005), Jensen et al. (2007) and Alho et al. (2009) generally reported negative weak correlation coefficient values between heterozygosity and pedigree-based inbreeding coefficients, although the estimate for one of the populations (Scandinavian wolves) reached −0.72. The authors identified the effect of marker density and pedigree errors among other factors that impact the estimate of correlation coefficients between the two variables. Higher estimate of correlation in our study could be legitimate owing to high maker density and a better precision of genomic inbreeding estimates. Our findings reemphasize the utility of heterozygosity as proxy for inbreeding in the study of inbreeding depression in light of the availability of current genetic markers and where phenotypes are available.

### Genes under selection

The detection of ROH island in all our breeds allows for the investigation of selection signatures, which are a consequence of selection, recombination and genetic drift (Ceballos et al., 2018; Gorssen et al., 2020). Surprisingly, identified ROH islands were not shared across the three breeds in our study, rather, SM and the outgroup HUMA shared the same ROH island on SSC11 (80–86 Mb). By contrast, in an investigation of breed substructures in Pietrain pigs, Gorssen et al. (2020) found several ROH islands common to different populations of the same pig type, and even reported a large ROH island on SSC8 (34–126 Mb), which appears to be fixed. For our findings, it is compelling that BM, RM, and SM have been managed as separate breeds whose ROH patterns have been predominantly shaped through within-breed selection. Assuming that the HUMA genotypes are from Swallow-bellied Mangalitsa as suggested by our principal component analysis, the shared ROH island region of this breed with SM indicates a breed-specific signature of selection present in both the Hungarian and German populations. The shared ROH island between SM and HUMA was mapped to SSC11 (73–78 Mb) in our original analysis, where an updated reference assembly (*Sus scrofa* genome version 11.1) was used. One out of nine previously detected ROH islands in SM was mapped to SSC11, however, the exact position (33,516,188–36,493,548) differs from our finding (Zorc et al., 2022). The authors identified PCDH2*0* which is associated with brain activity and tameness in this region. They also found many other genes in regions some of which overlapped with those in Black Slavonian, Banija spotted and Moravka pig breeds. To investigate candidate genes in our study, we limited our scope to 100 kb distance flanking the significant SNPs defining ROH islands (Table 3). The proximal, genes ARHGEF7 (SSC11: 77,427,368–77,530,995) and EFNB2 (SSC11: 74,186,64–4,233,354) appear to be important in the SM and HUMA. ARHGEF7 is associated with the storage of materials, including nutrients, pigments and waste products (GO:0000322), and might influence the coat color pattern in SM. Among others, EFNB2 plays a role in the negative regulation of neuron projection development (GO:0010977), a positive regulation of neuron death (GO:1901216) and the movement of cells in response to specific chemical signals (GO:0050920). The gene has been suggested as a candidate for hearing and visual impairment, and pigmentary anomalies in human (Lévy et al., 2018). Distinguishing between Landrace and Yorkshire pigs *via* fixation index analysis, Wang et al. (2021) found EFNB2 to have been under intense selection pressure.

The second ROH island in SM (SSC17: 16–26), which was neither shared by HUMA nor BM and RM revealed a high level of selection for the kinesin family member 16B (KIF16B) gene. Interestingly, the same gene also popped up while comparing SM to both BM and RM (Table 1). Located on SSC17 (24,871,985–25,188,204), the 31,6219 BP length KIF16B gene is predominantly involved in microtubule-based movement (GO:0007018). By this, it plays an important role in regulating early development and organogenesis such as in embryos, kidneys and in stem cells (Hirokawa and Tanaka 2015). KIF16B gene was found to significantly influence wool length and greasy yield in fine wool sheep (Wang et al., 2014; Zhao et al., 2021). In humans it has been associated with synaptic signaling, which confers intellectual abilities (Loo et al., 2012). Therefore, SM may differ from the other breeds in terms of cognitive ability. To our knowledge, this has not yet been investigated in our breeds. Nevertheless, several studies have shown general differences in cognitive abilities in pigs (Gieling et al., 2011). The ROH region in SM also harbors other important genes—BANF2 and BTBD3 are associated with male (Omolaoye et al., 2022) and female (Kim et al., 2012) fertility in pigs; ESF1 is associated with meat quality in pigs (Puig-Oliveras et al., 2014); and MACROD2 is reported to be associated with disease resistance in cattle (González-Ruiz et al., 2019).

Autozygosity in RM is especially related to genes associated with meat quality including ABCA12 (Wang Xiao et al., 2019), VIL1 (Zhang et al., 2015; Verardo et al., 2017), PLSCR5 (Wang Zezhao et al., 2019) and USP37 (Verardo et al., 2017). Meat quality assessment in Mangalitsa pigs has been very general and shown that meat from these pigs have higher intramuscular fat content compared to a commercial pig breed (Stanišić et al., 2016). Nistor et al. (2012) found significant differences in the ratio of saturated and unsaturated fatty acids in RM and BM meat (35.88%:62.76% and 38.42%:59.94%, respectively). Although not properly documented, we know that among German Mangalitsa pig breeders, RM is mostly preferred owing to a perceived higher meat quality in the breed. Also, of importance in RM are genes previously reported to be associated with characteristics such as feed efficiency—CTSH (Russo et al., 2008) and VWC2L (Kejun et al., 2015); growth—RASGRF1 (Magee et al., 2010); and fertility—SPATA7 (Marques et al., 2018), TNP1 (Gòdia et al., 2020) and WWTR1 (Budna et al., 2017). In Egerszegi et al. (2003), RM had the highest body weight of 220 kg and 180 kg compared to lowest values of 165 kg and 170 kg in SM boars and sows, respectively.

Identified in the ROH island of BM, the PRLR gene is reported to play a role in prolactin signaling (GO:0038161), lactation (GO:0007595), mammary gland development (GO:0060644) and response to bacterium stimuli (GO:0009617). Studies on lactation in Mangalitsa pigs are lacking, however, there is one that only reported a mean lactation length of 52.57 days in BM (Petrović et al., 2013).

The occurrence of identical loci under divergent selection for the pairwise comparison of BMSM and RMSM signals similarity between BM and RM on one hand, and on the other hand, differences between each of the pair and SM. Inferring from the associated genes, characteristics such as behavior influenced by SPAG4 (Bélteky et al., 2016; Bélteky et al., 2017); body size by STIM2 (Osei-Amponsah et al., 2017); carcass and fat deposition by HNF4A (Fan et al., 2010; Alexandre et al., 2014; MoreiraMonterio et al., 2018; Criado-Mesas et al., 2020) and CEP250 (Damon et al., 2012); muscularity by CPNE1 (Dawei et al., 2010); and disease resistance by NFS1 (Dauben et al., 2021) are more likely to be similar between BM and RM than between each of these and SM. However, this may not be entirely true if several other differentially expressed gene control these traits. The RMBM comparison showed the lowest differentiation in this study, for which the SNP with the highest signal (M1GA0011680) was found proximal to an autoregulatory α-adrenergic receptor 2C (ADRA2C) gene involved in the regulation of smooth muscle contraction among others (GO:0006940). Contrasting RM and BM, Bâlteanu et al. (2021) previously found the region SSC16 (18–20 Mb) to have a potential effect on hair pigmentation. Their findings were, however, based on selection scans with HapFLK, BAYESACN and GWAS, and furthermore, the analysis of gene content which revealed the solute carrier family 45-member 2 (SLC45A2) locus as a candidate gene.

Since their development, Mangalitsa pigs have evolved differentially as evidenced by both intra- and inter-population statistics in the current and previous studies. Adjudged by F_st_ estimates, the degree of differentiation, which peaked at about 9.5% (BMSM) in the current study is low compared to those in other breeds such as the Turopolje, being 21% on average (Druml et al., 2012). Besides, Mangalitsa pigs are collectively described as endangered and require conservation interventions. The maintenance of small populations under restricted geneflow between animals of different coat colors raises concerns about long-term implications for the conservation of genetic diversity. In our original data, 17 animals were said to be crossbreds from the three main breeds, meaning that some German breeders already practice crossbreeding amongst different Mangalitsa pig breeds. The lack of pedigree records makes it difficult to confirm this type of cross, and to use such animals in our analyses. The crossing of different Mangalitsa breeds by German breeders is not yet documented, however, possible reason for crossbreeding in these pigs can be the favorable effect of breed complementarity and the avoidance of inbreeding.

## Conclusion

In this study, we provided an insight into the differences in the three Mangalitsa pigs breeds using a medium density SNP information. The Blond and Red Mangalitsa breeds are more similar to each other than each of them to the Swallow-bellied Mangalitsa. This finding sharply contrasts previous reports of the lowest differentiation between Blond and Swallow-bellied Mangalitsa pigs. Genetic diversity was highest in Red Mangalitsa; however, inbreeding was considerably high in all the breeds. Highly homozygous genomic regions were not shared across breeds and this, to a large extent, emphasizes the restriction of geneflow among the breeds. We found several breed specific signatures of selection including those that may underline growth and meat quality traits in Red Mangalitsa or that suggest intellectual ability in Swallow-bellied Mangalitsa. By providing a list of candidate genes for all genome-wide significant variants, we propose further investigations that would link these genes to actual phenotypes where available. Furthermore, we would add Manhattan plots of our ROH incidence to the repository of ROH islands for comparisons with future studies.

## Data Availability

The datasets presented in this study can be found in online repositories. The names of the repository/repositories and accession number(s) can be found in the article/Supplementary Material.

## Supplementary material

Supplementary Figure 1 Cross-validation for varying number of clusters ranging from k = 2 to k = 20 in Mangalitsa pigs.

Supplementary Figure 2 ROH incidence plots for each chromosome in Blond Mangalitsa pigs.

Supplementary Figure 3 ROH incidence plots for each chromosome in Red Mangalitsa pigs.

Supplementary Figure 4 ROH incidence plots for each chromosome in Swallow-bellied Mangalitsa pigs.

Supplementary Table 1 SNPs in ROH island in Blond (BM), Red (RM) and Swallow-bellied (SM) Mangalitsa pigs. SNP positions were mapped to the Sus scrofa genome version 10.2.

Supplementary Data 1 Plink formatted files consisting of Blond (BM), Red (RM) and Swallow-bellied Mangalitsa (SM) genotypes.
