# Supplementary figures and images for: An insight into the runs of homozygosity distribution and breed differentiation in Mangalitsa pigs
Source: Front Genet. 2022 Oct 18;13:909986. doi: 10.3389/fgene.2022.909986 (PMC9632489; doi:10.3389/fgene.2022.909986)

N= 30

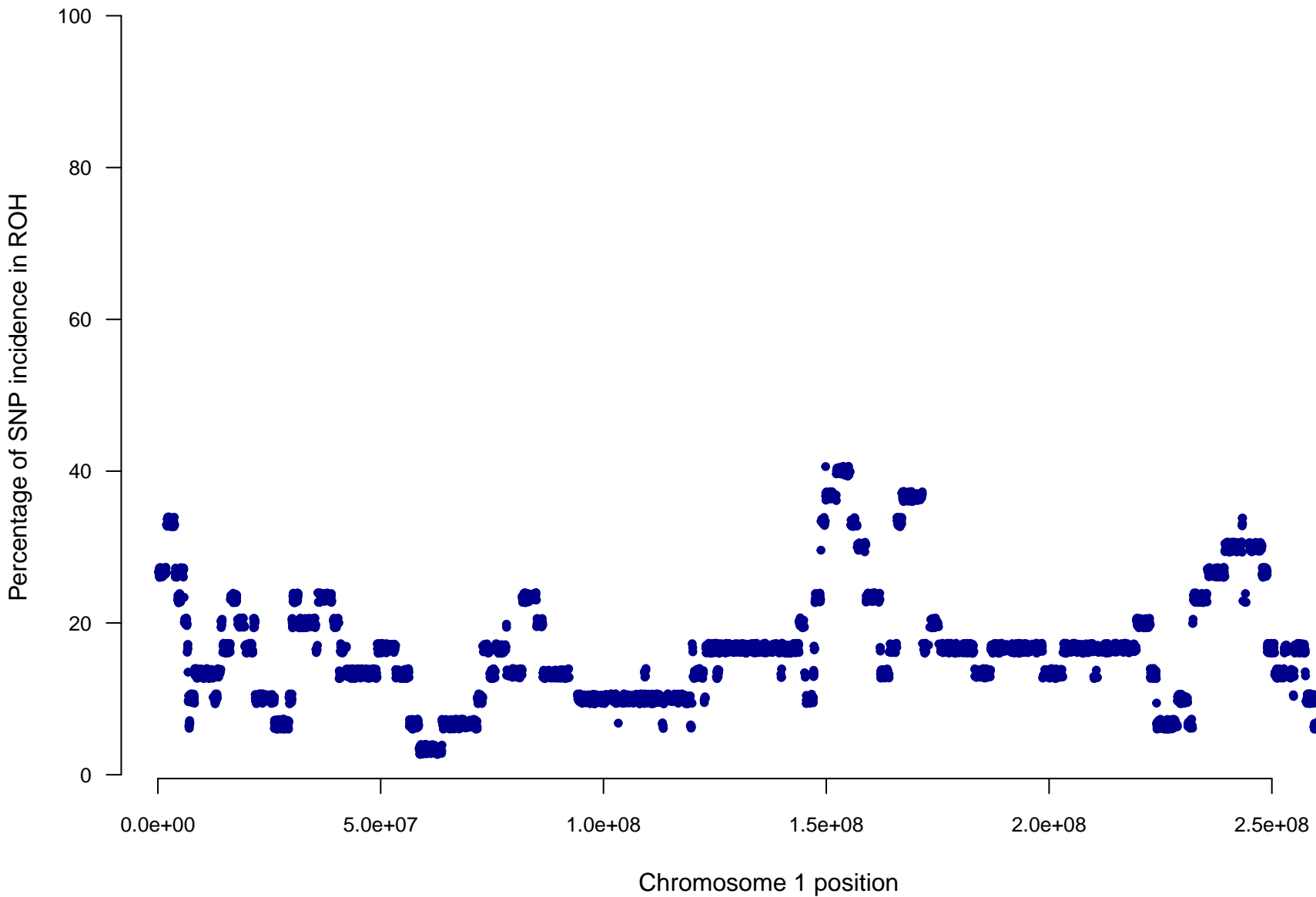

SM  
N= 30

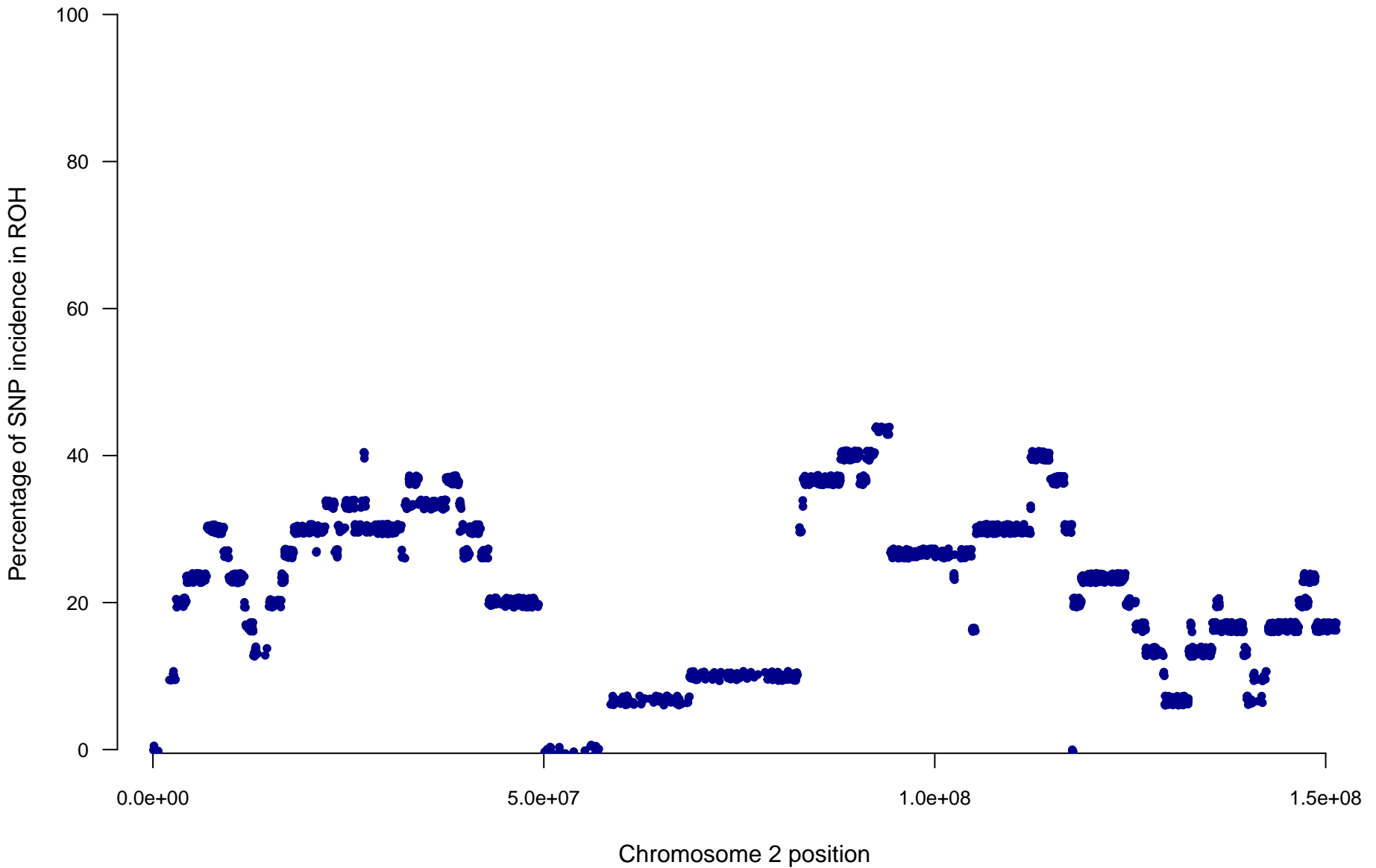

SM  
N= 30

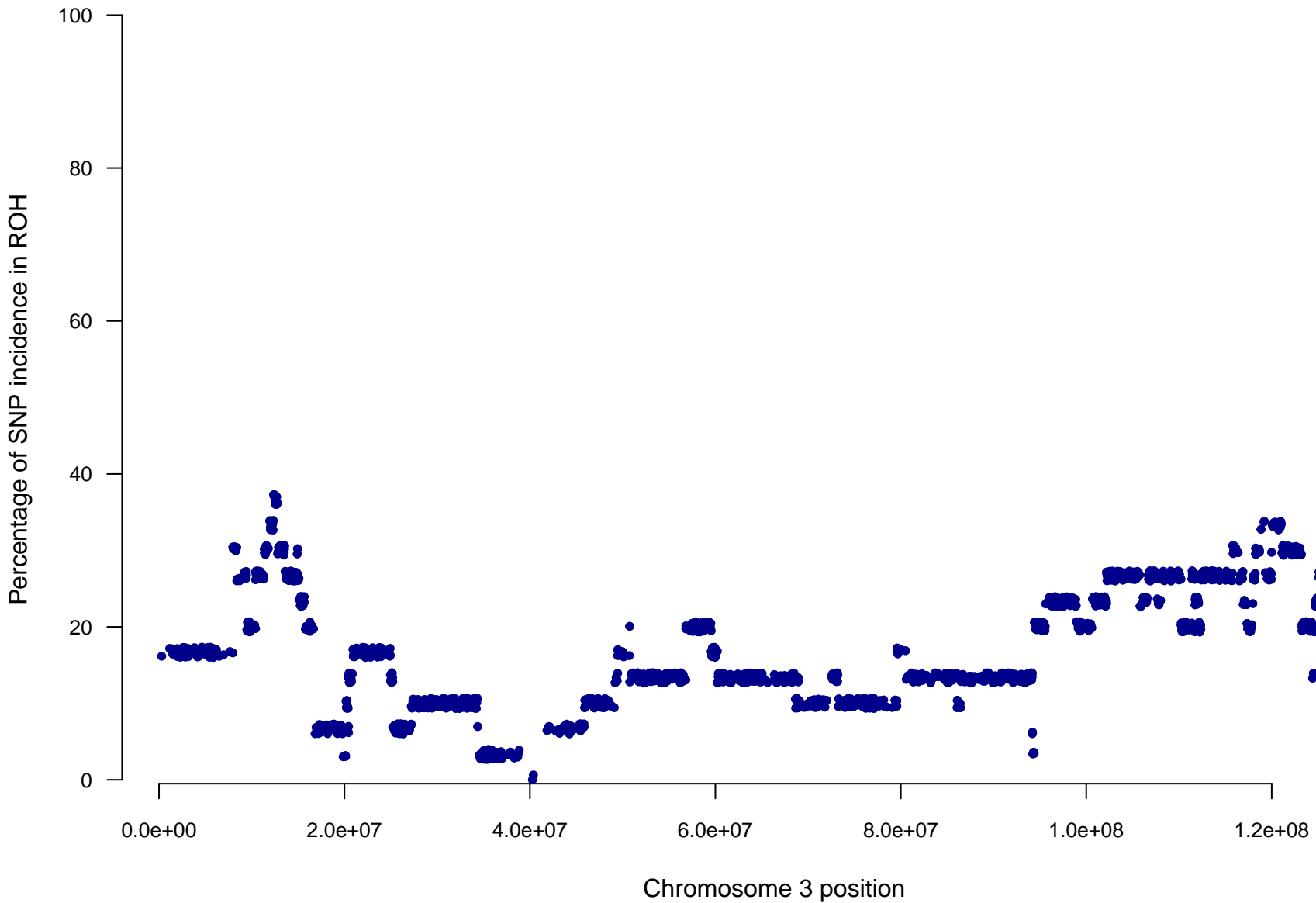

SM  
N= 30

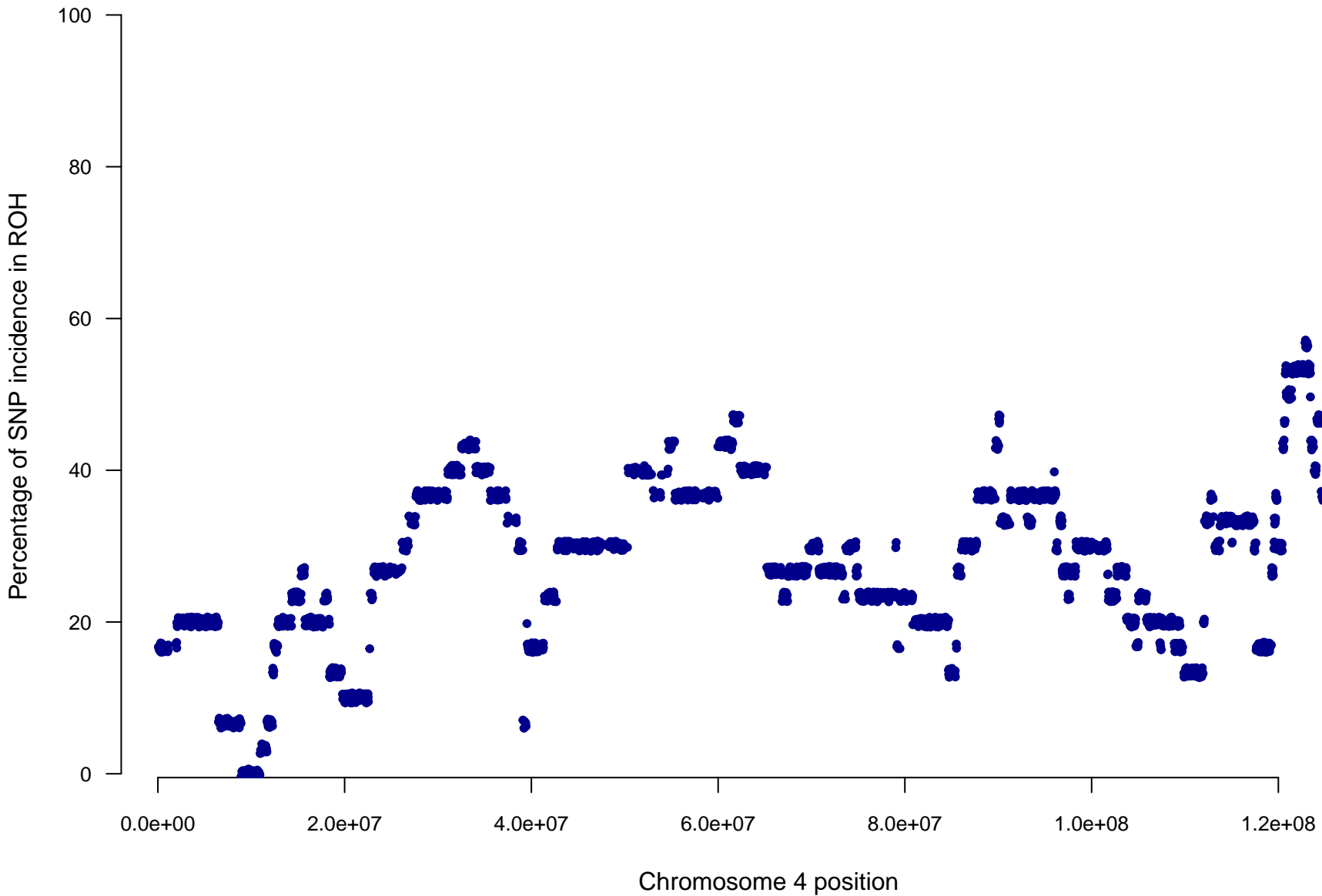

SM  
N= 30

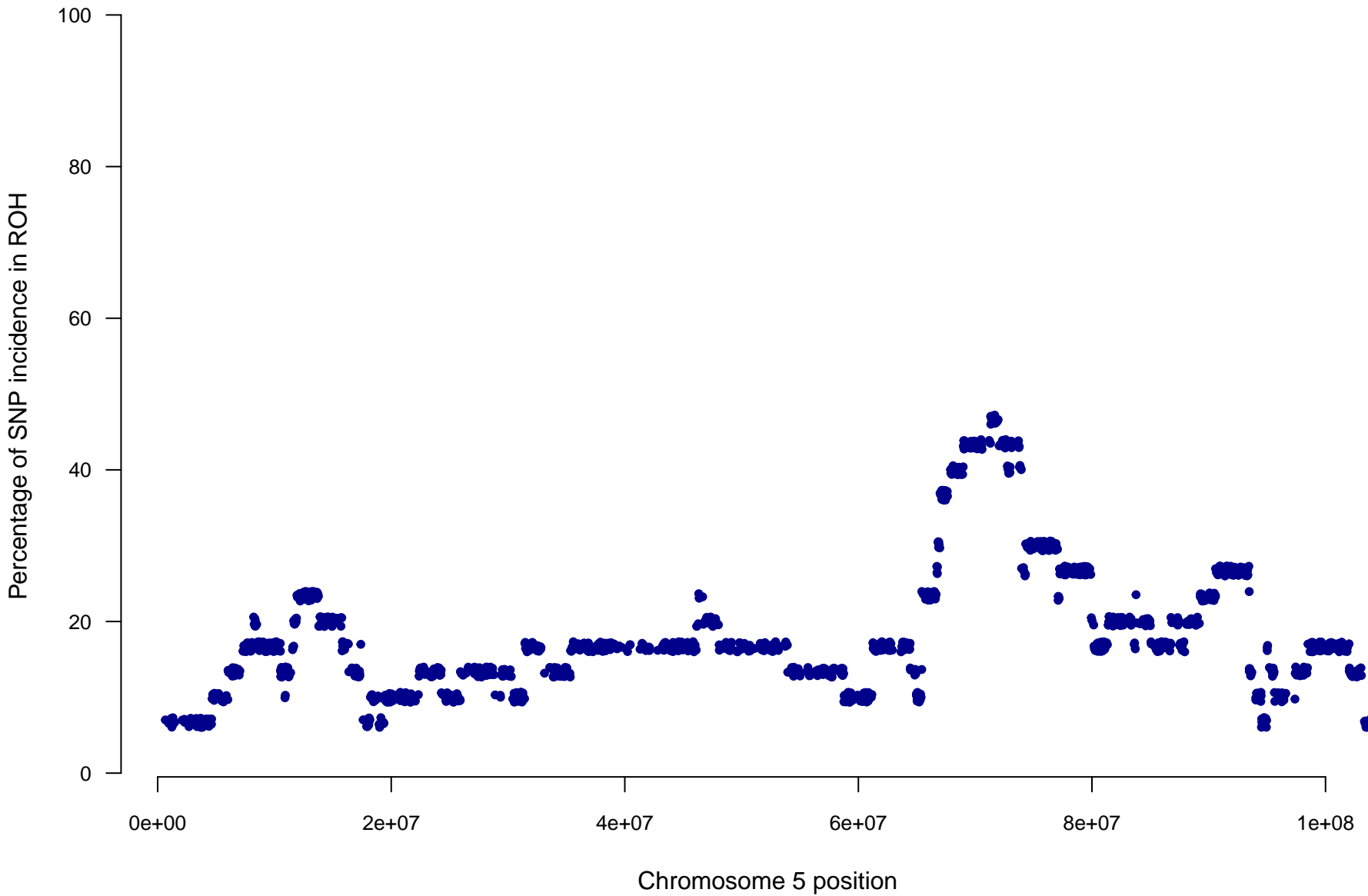

SM  
N= 30

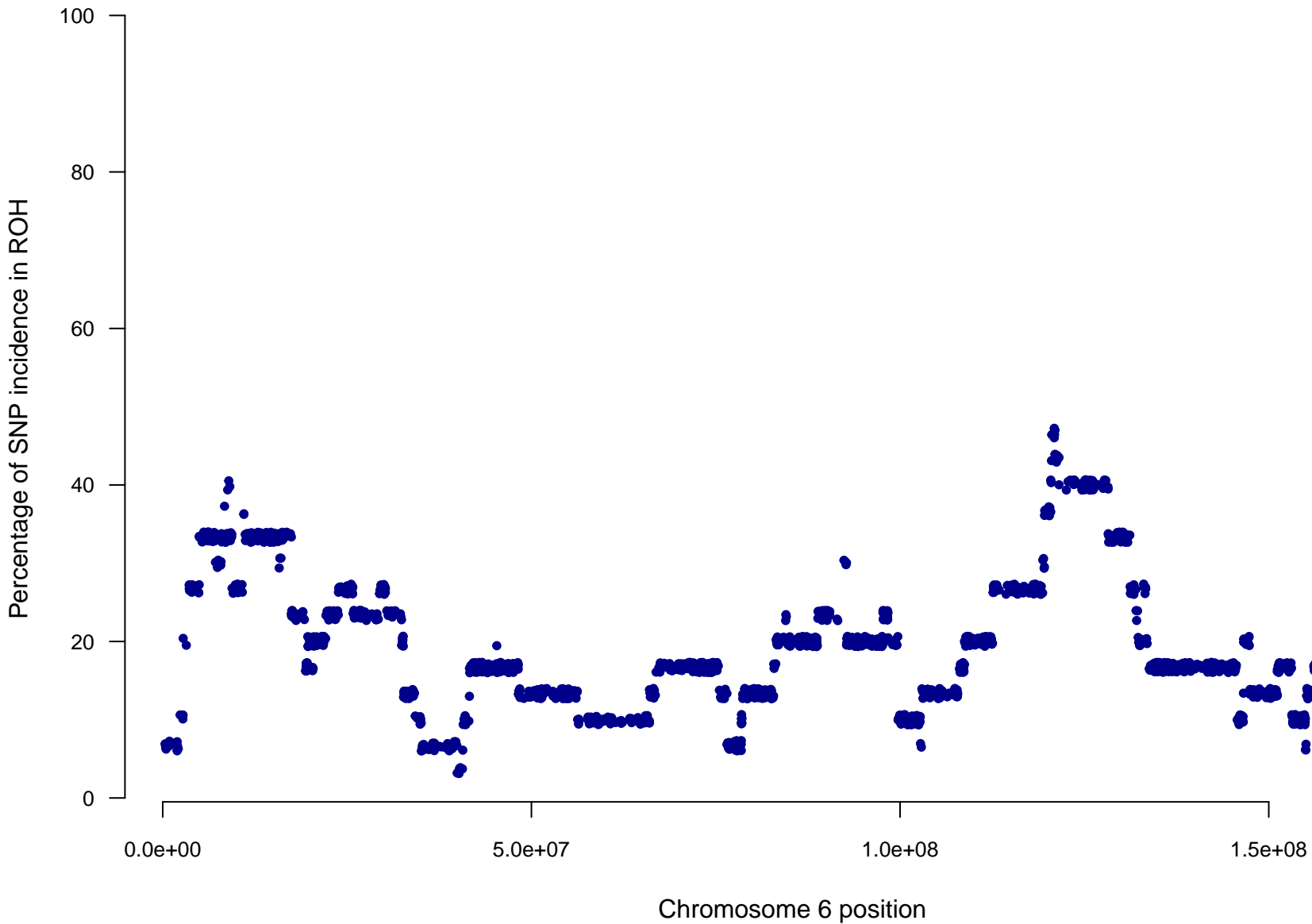

SM  
N= 30

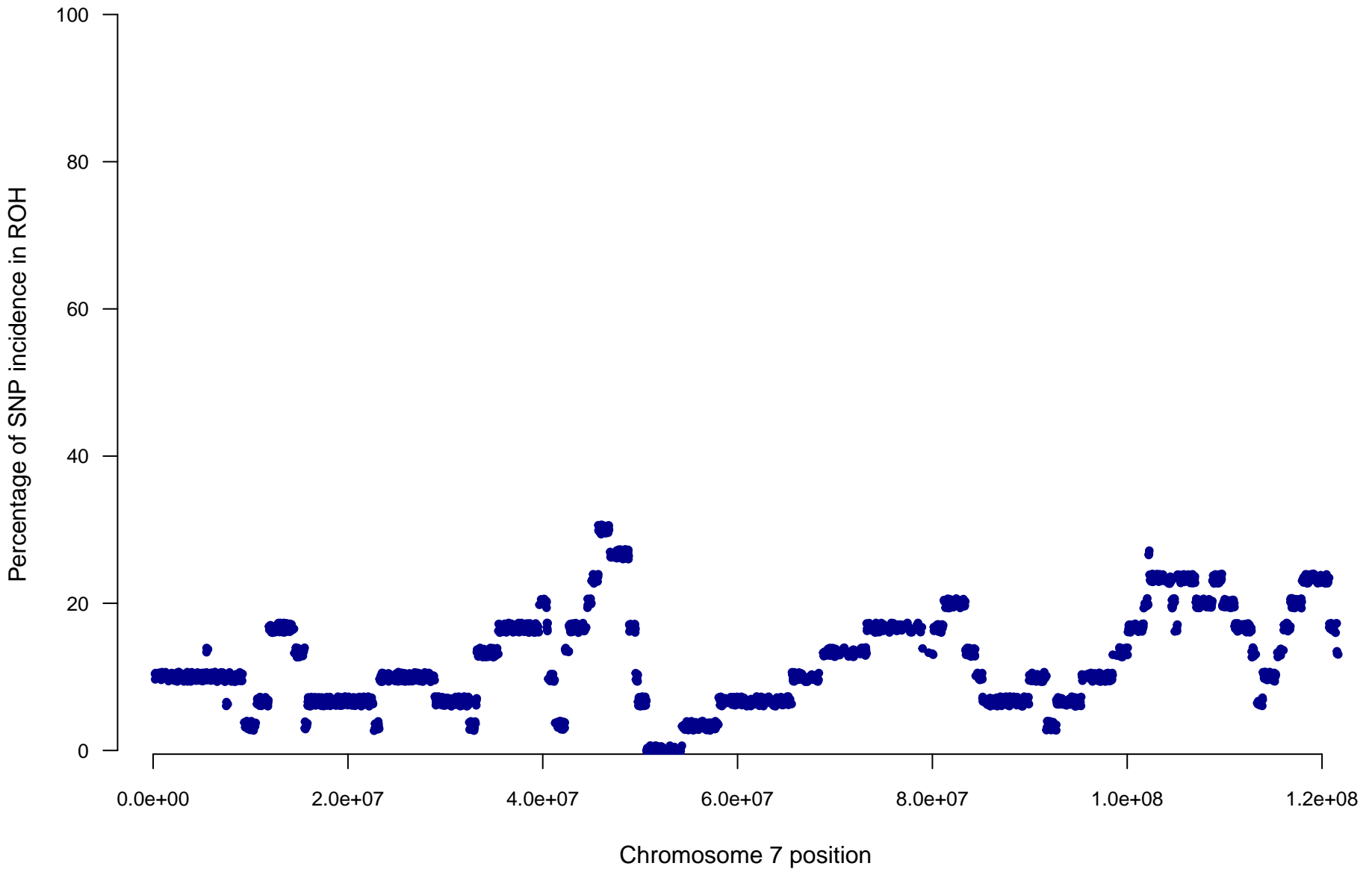

SM  
N= 30

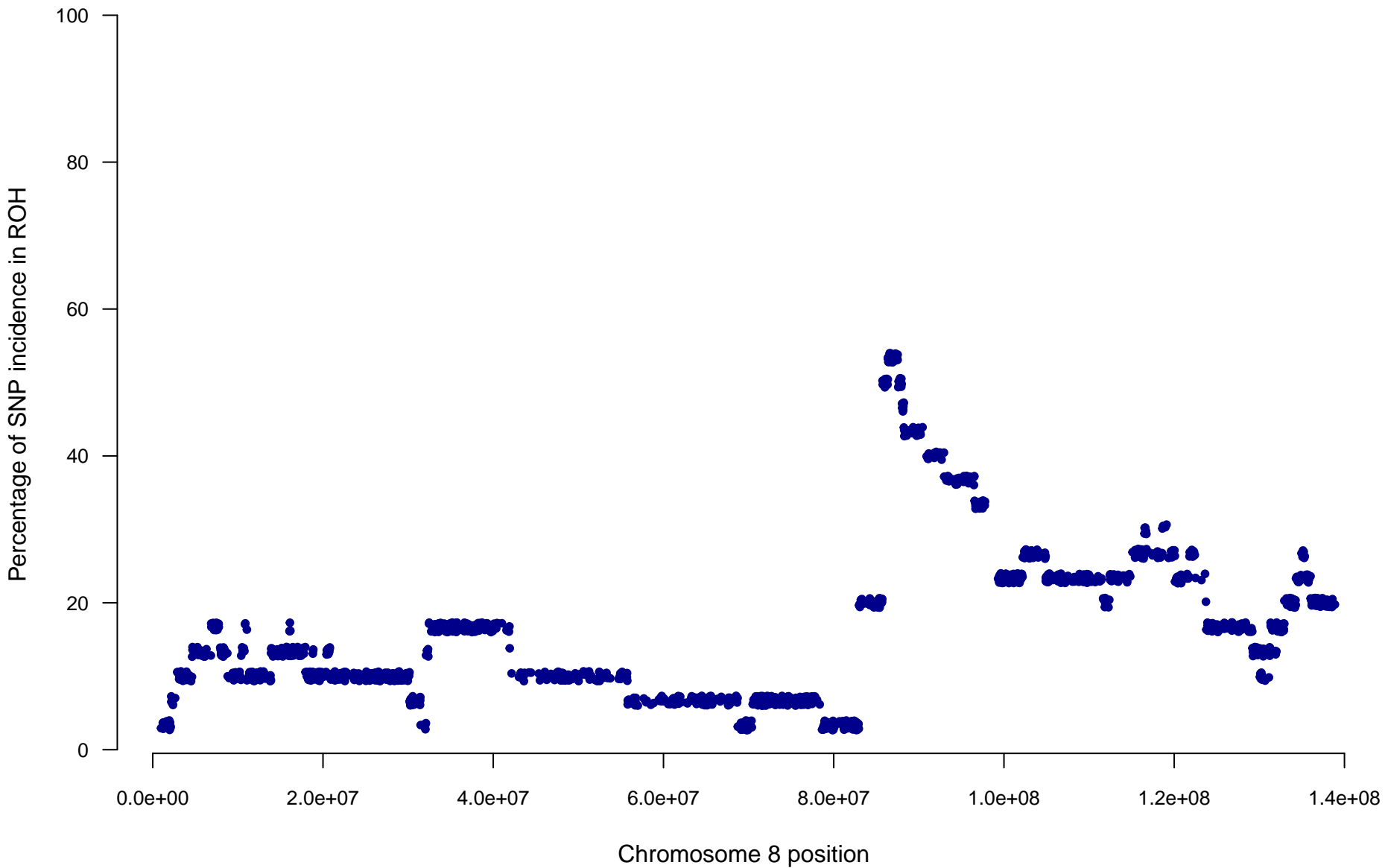

SM  
N= 30

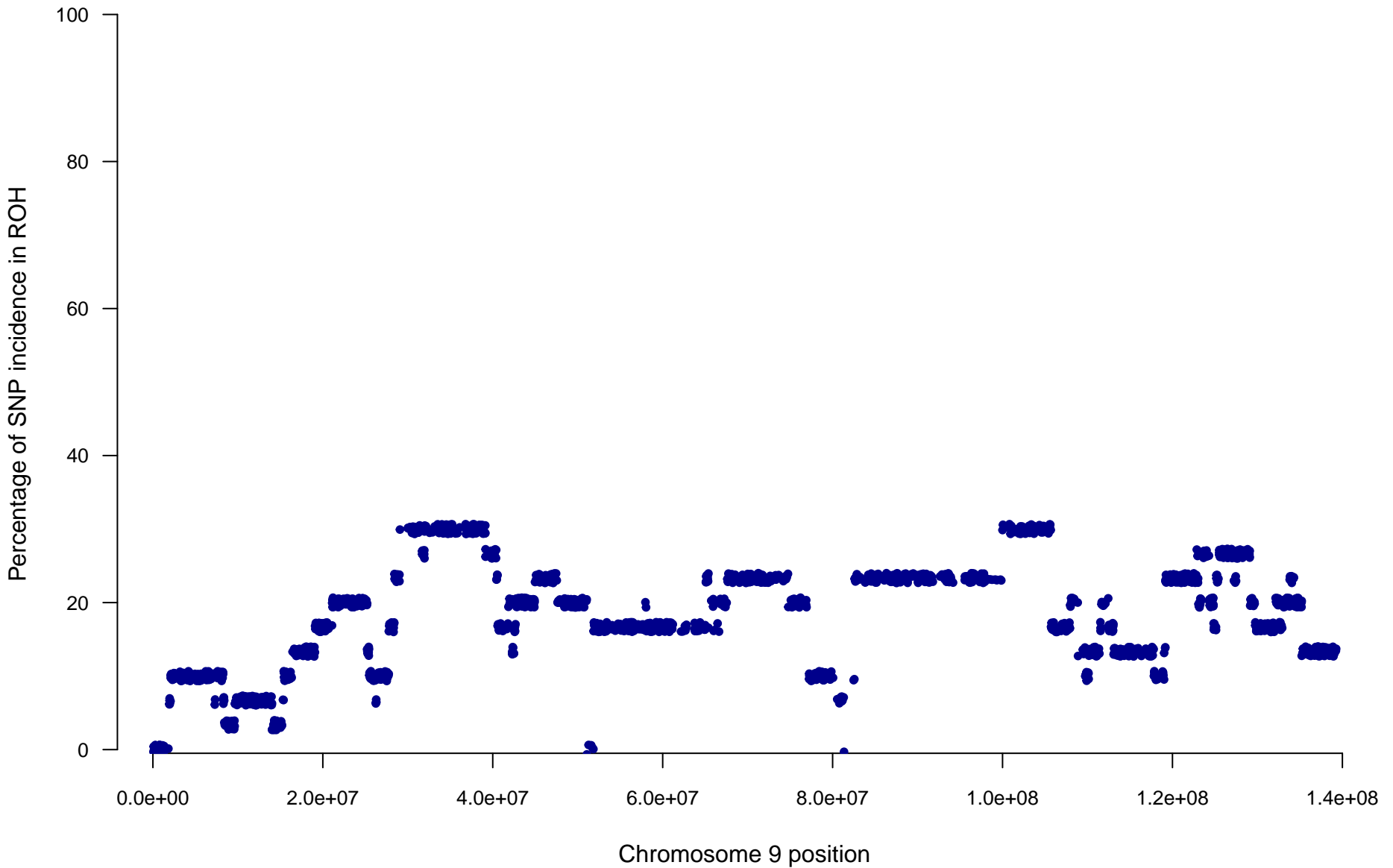

SM  
N= 30

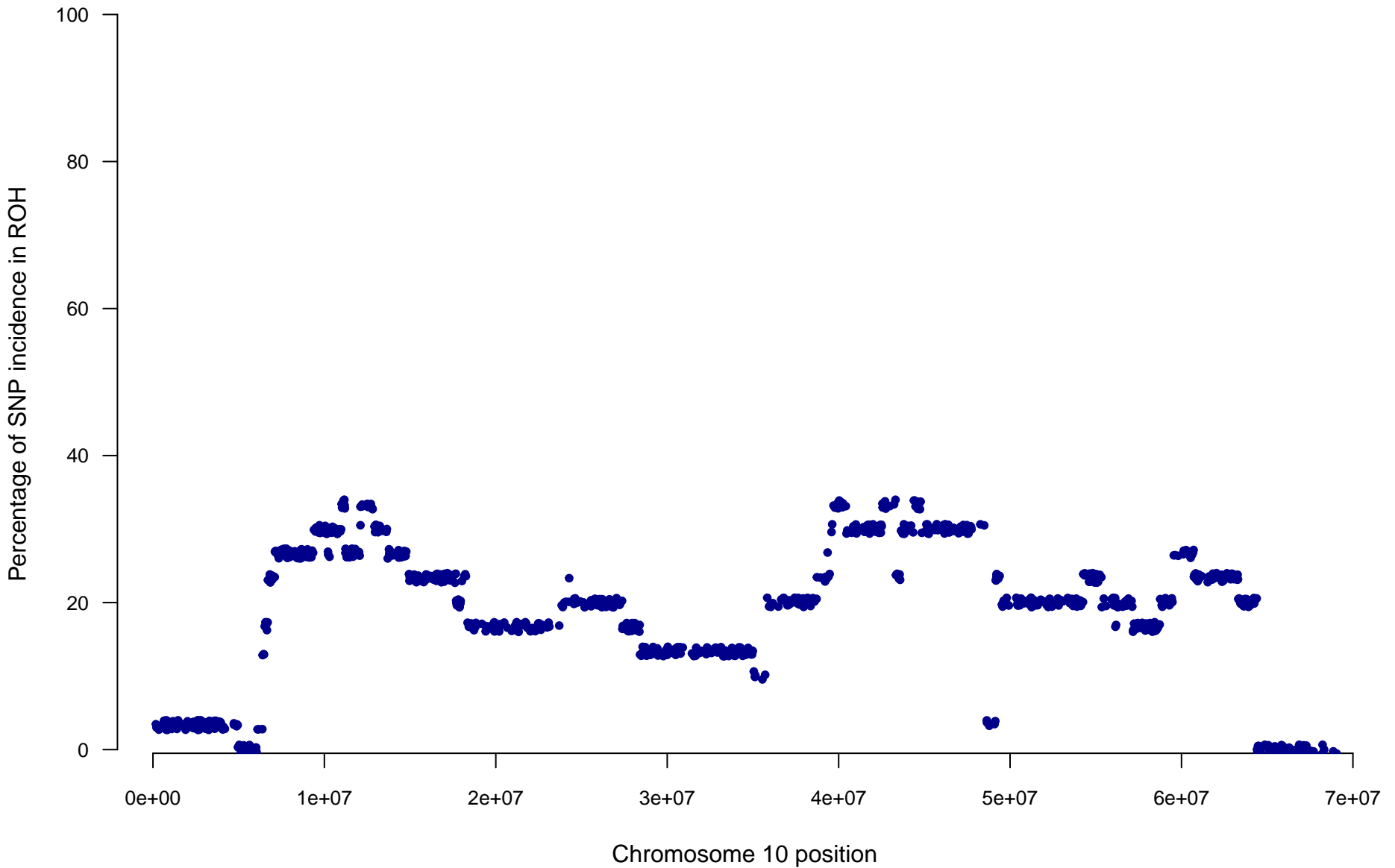

SM  
N= 30

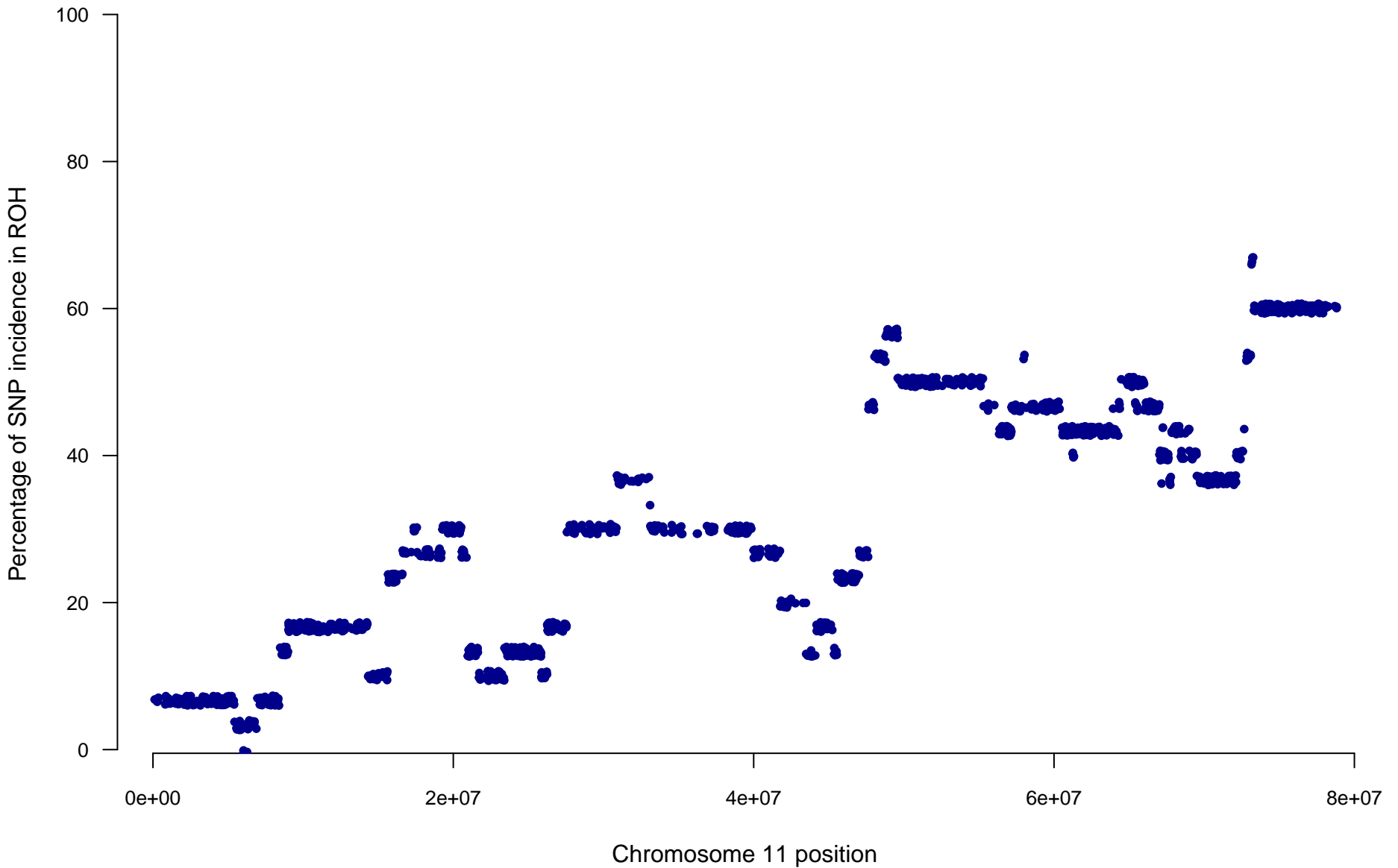

SM  
N= 30

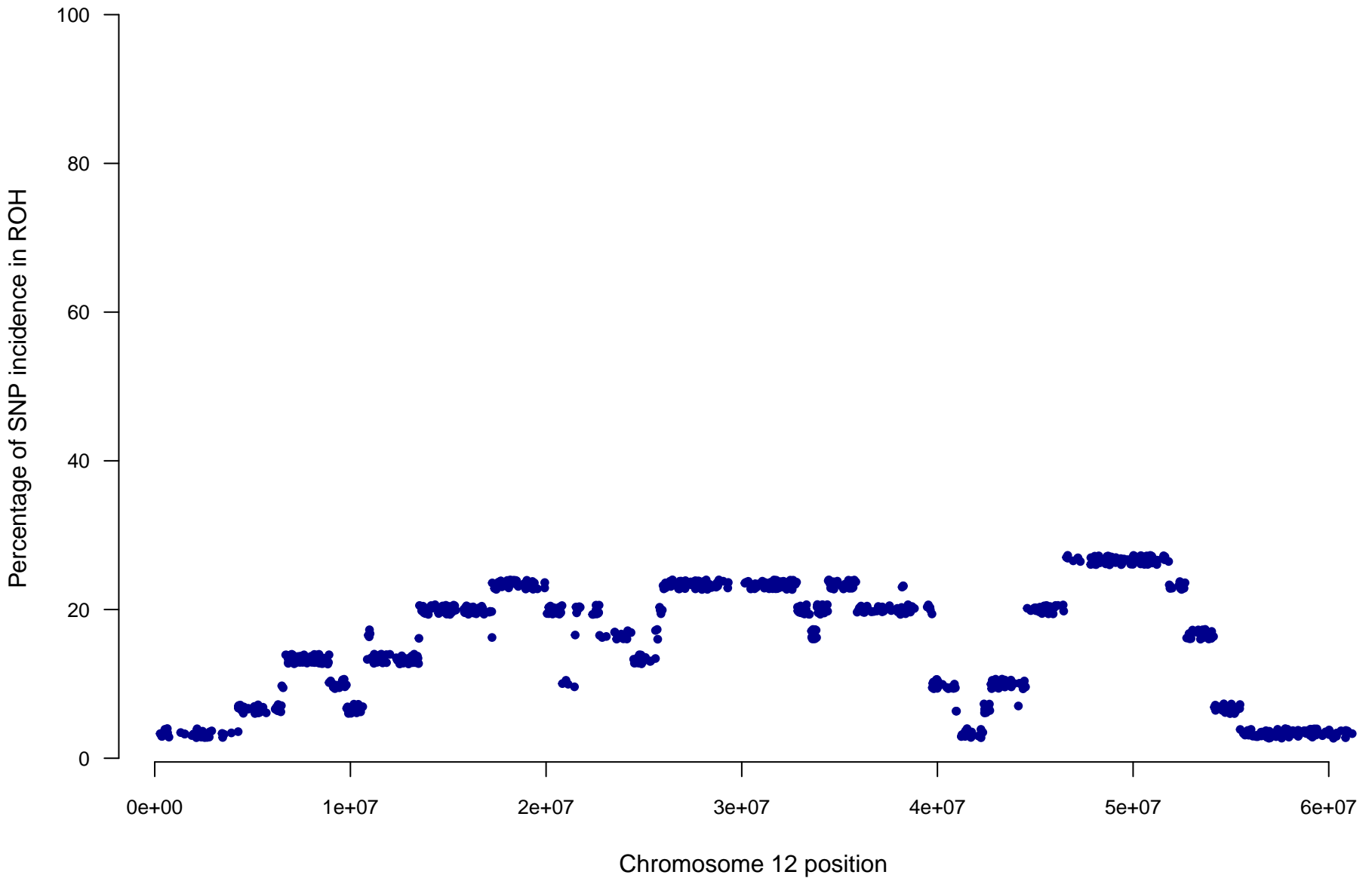

SM  
N= 30

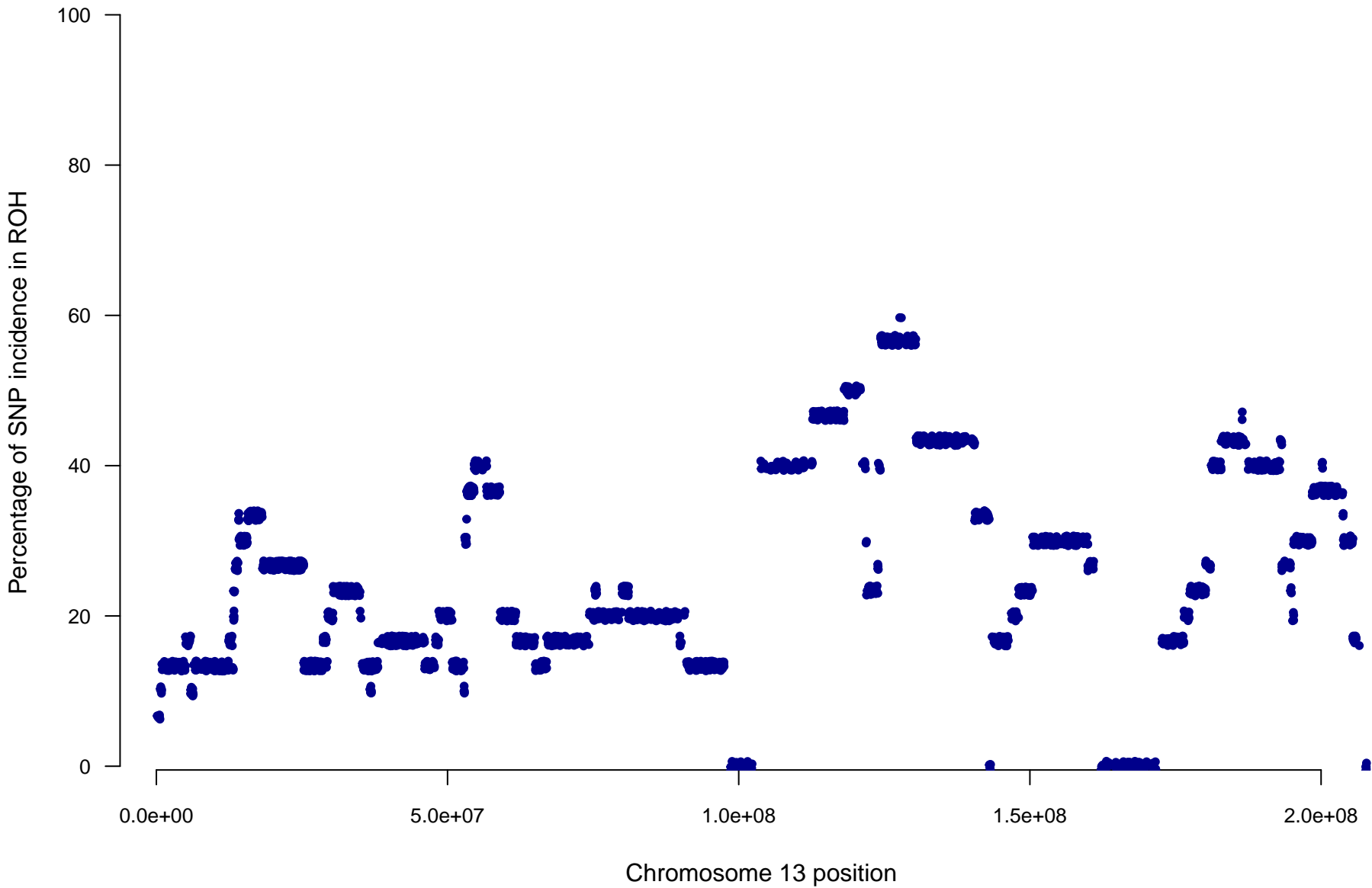

SM  
N= 30

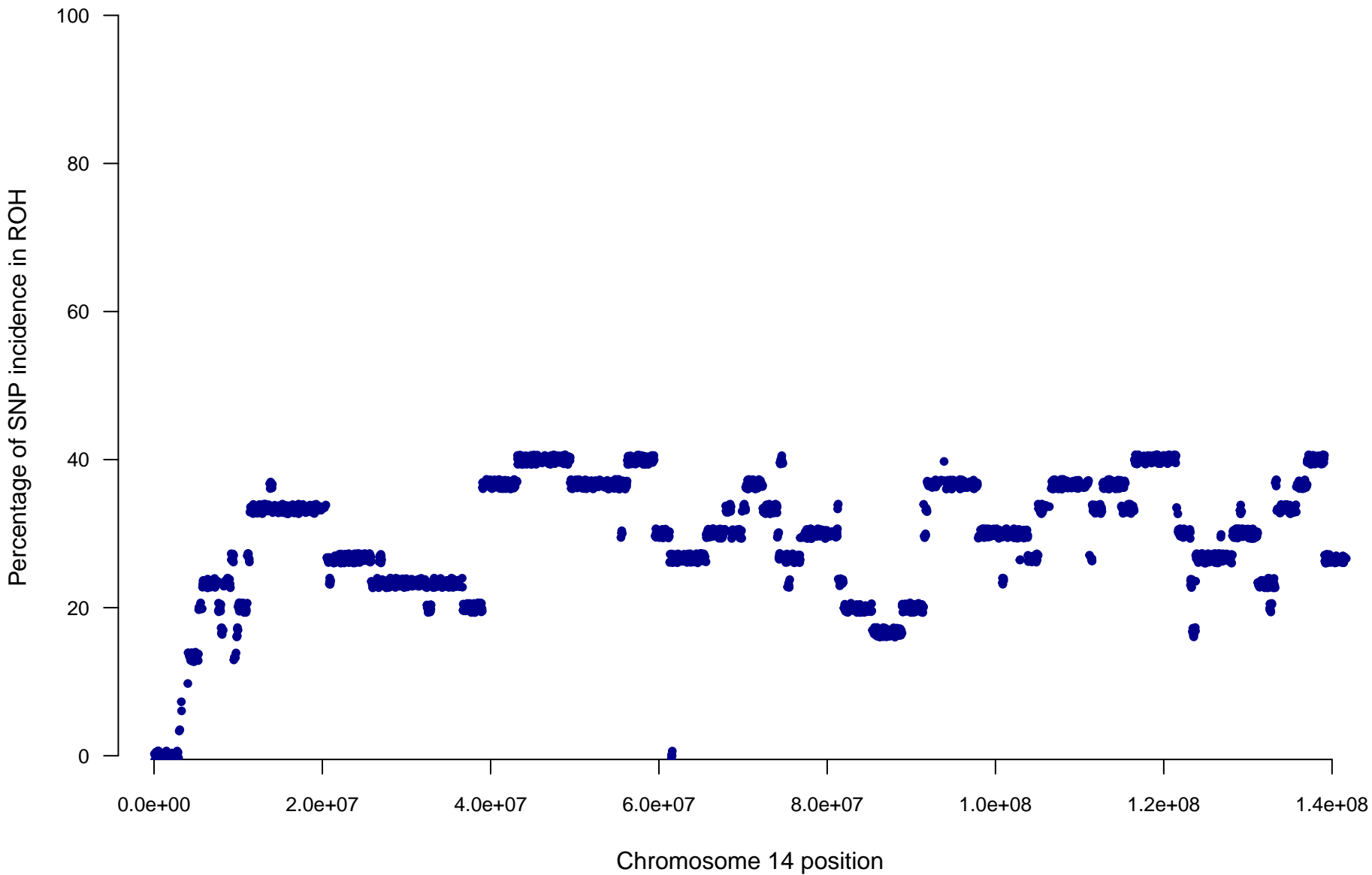

SM  
N= 30

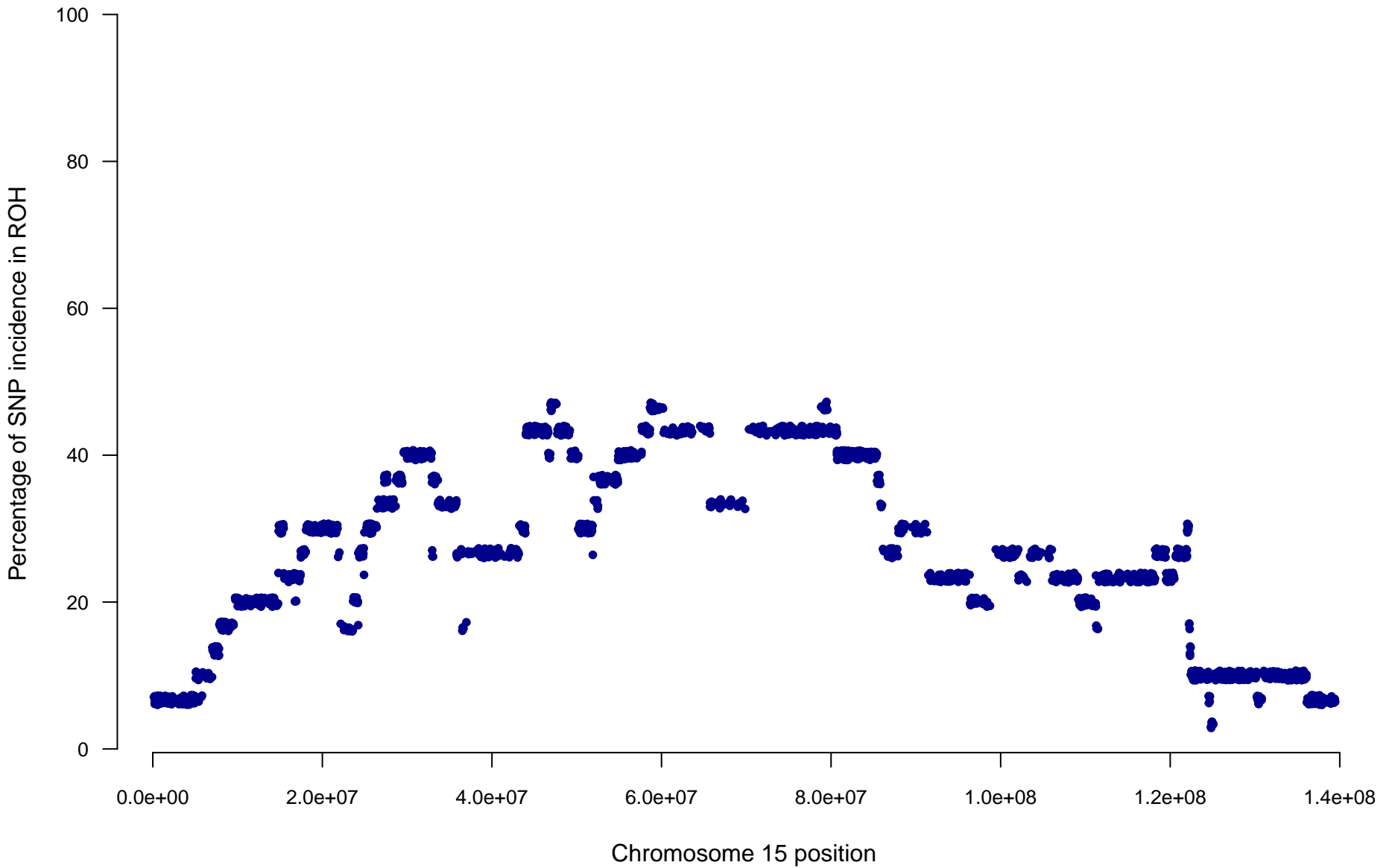

SM  
N= 30

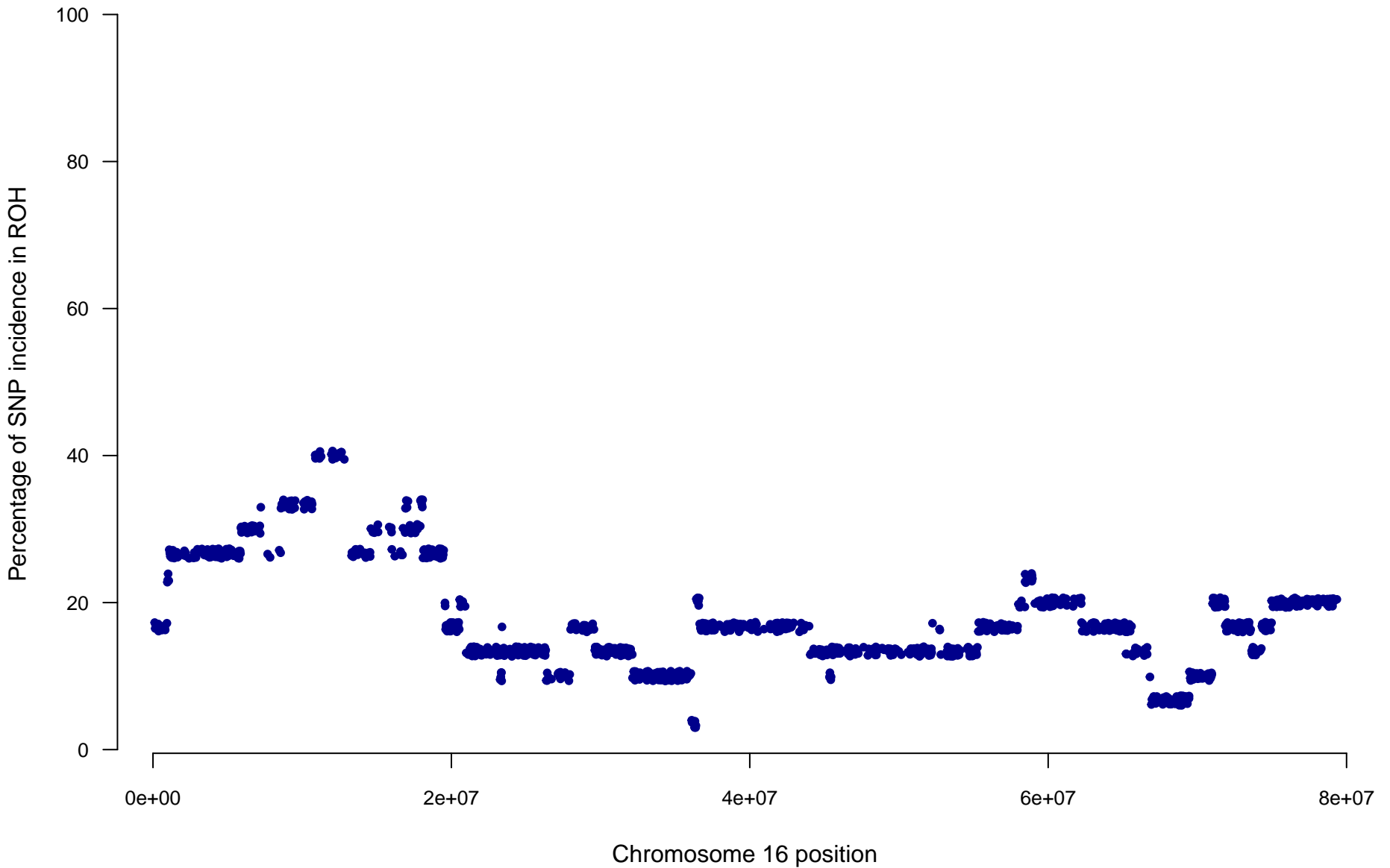

SM  
N= 30

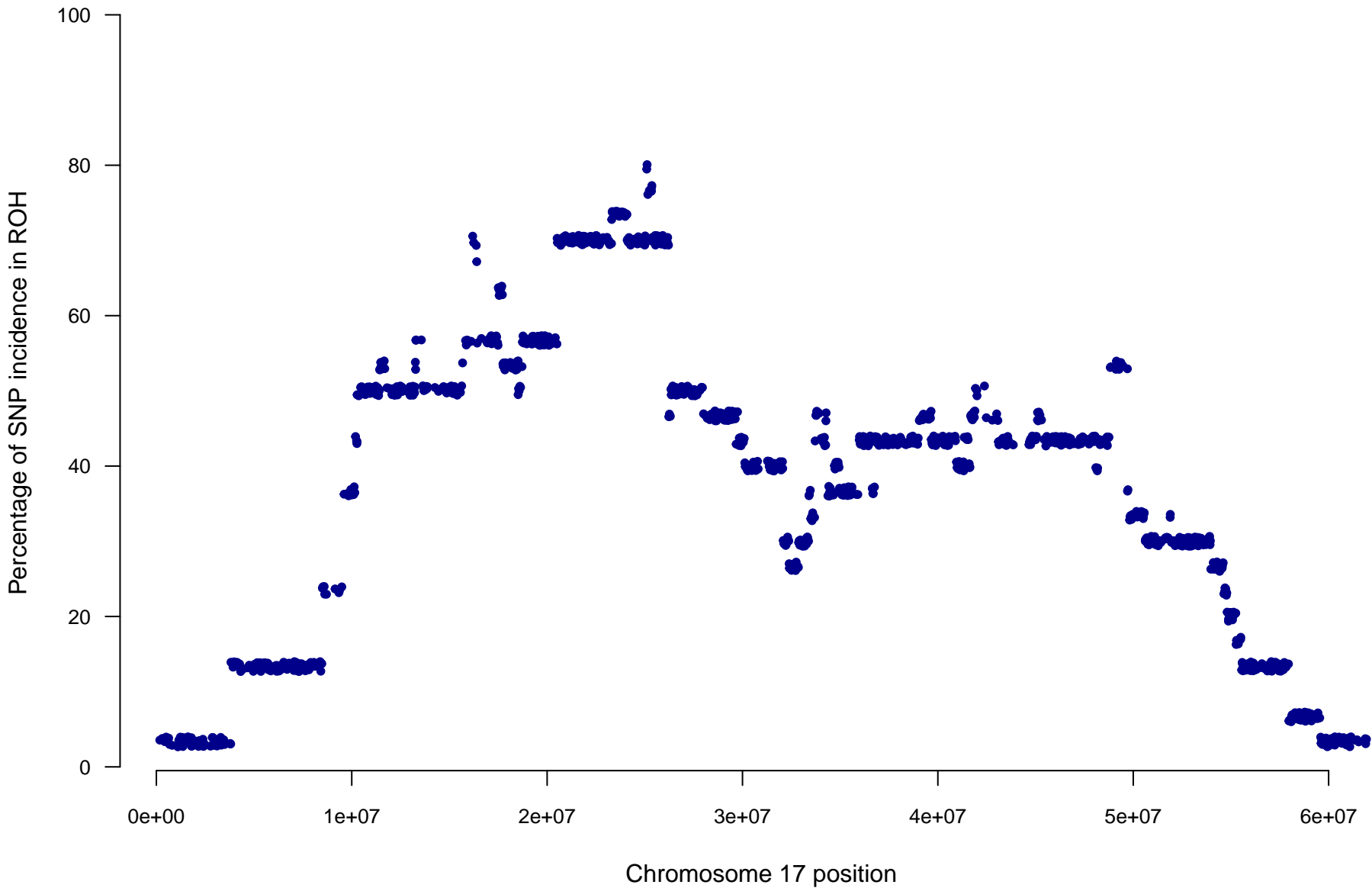

SM  
N= 30

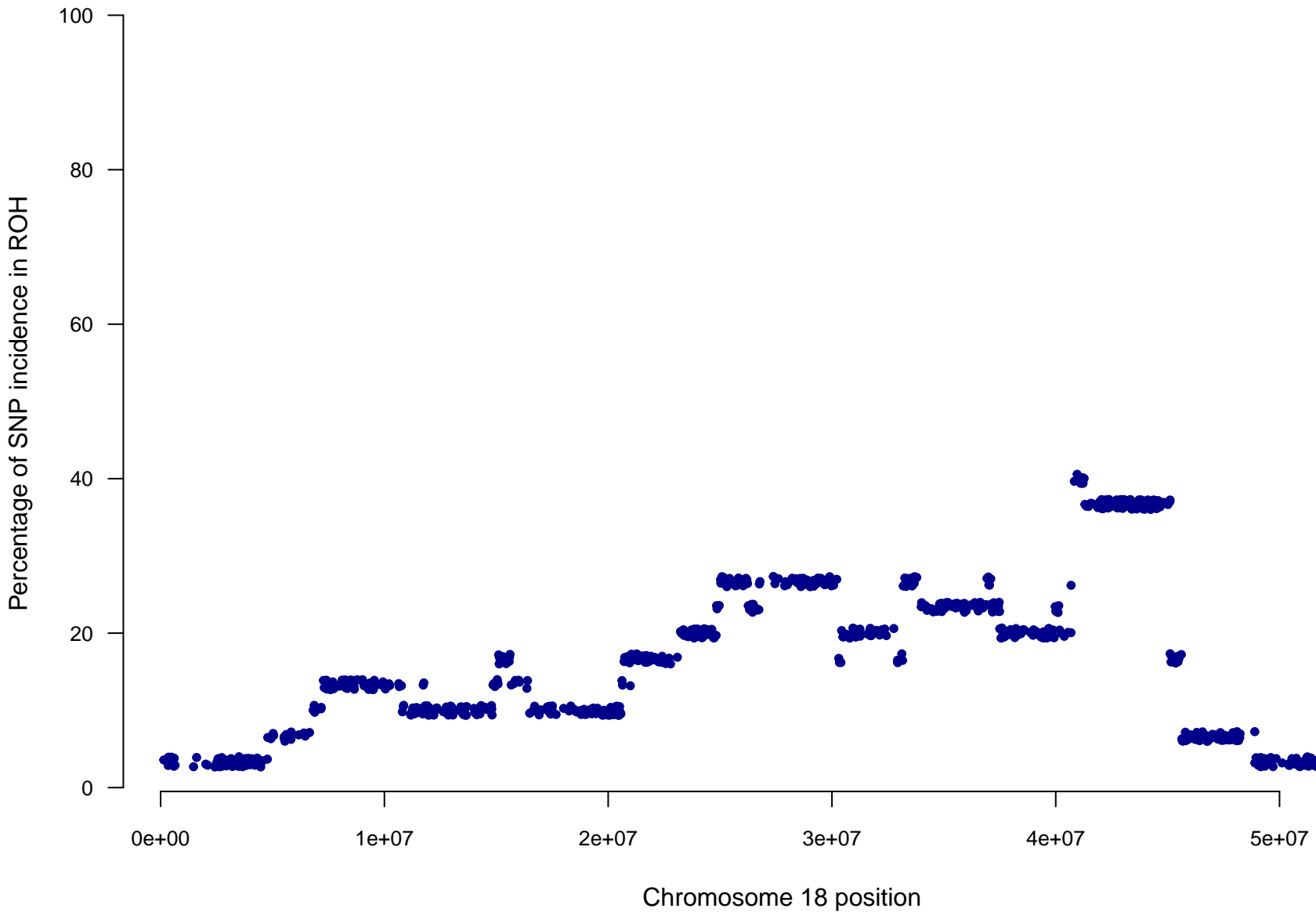

Supplement: Supplementary file 4 [file Image4.pdf]

BM  
N= 23

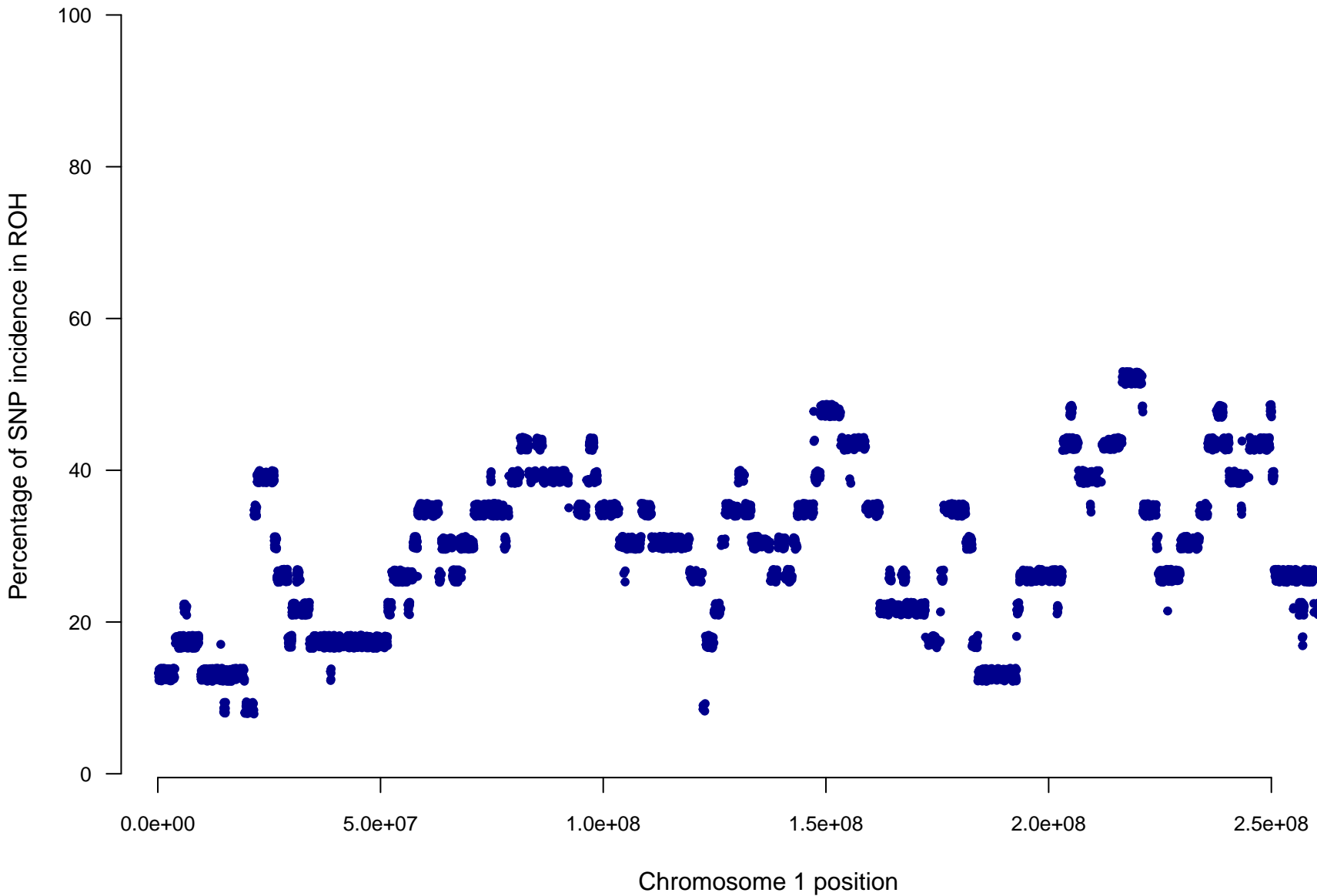

BM  
N= 23

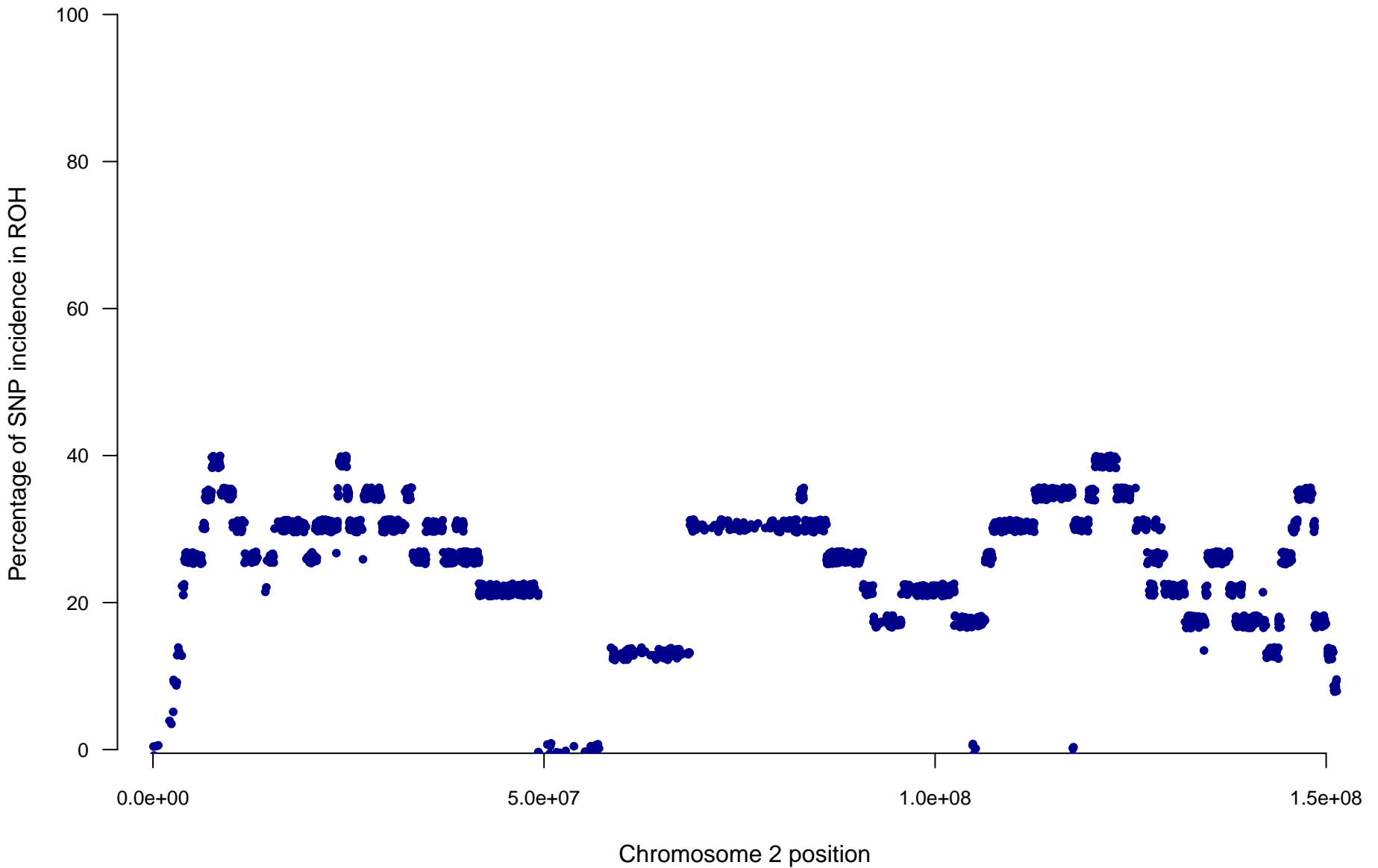

BM  
N= 23

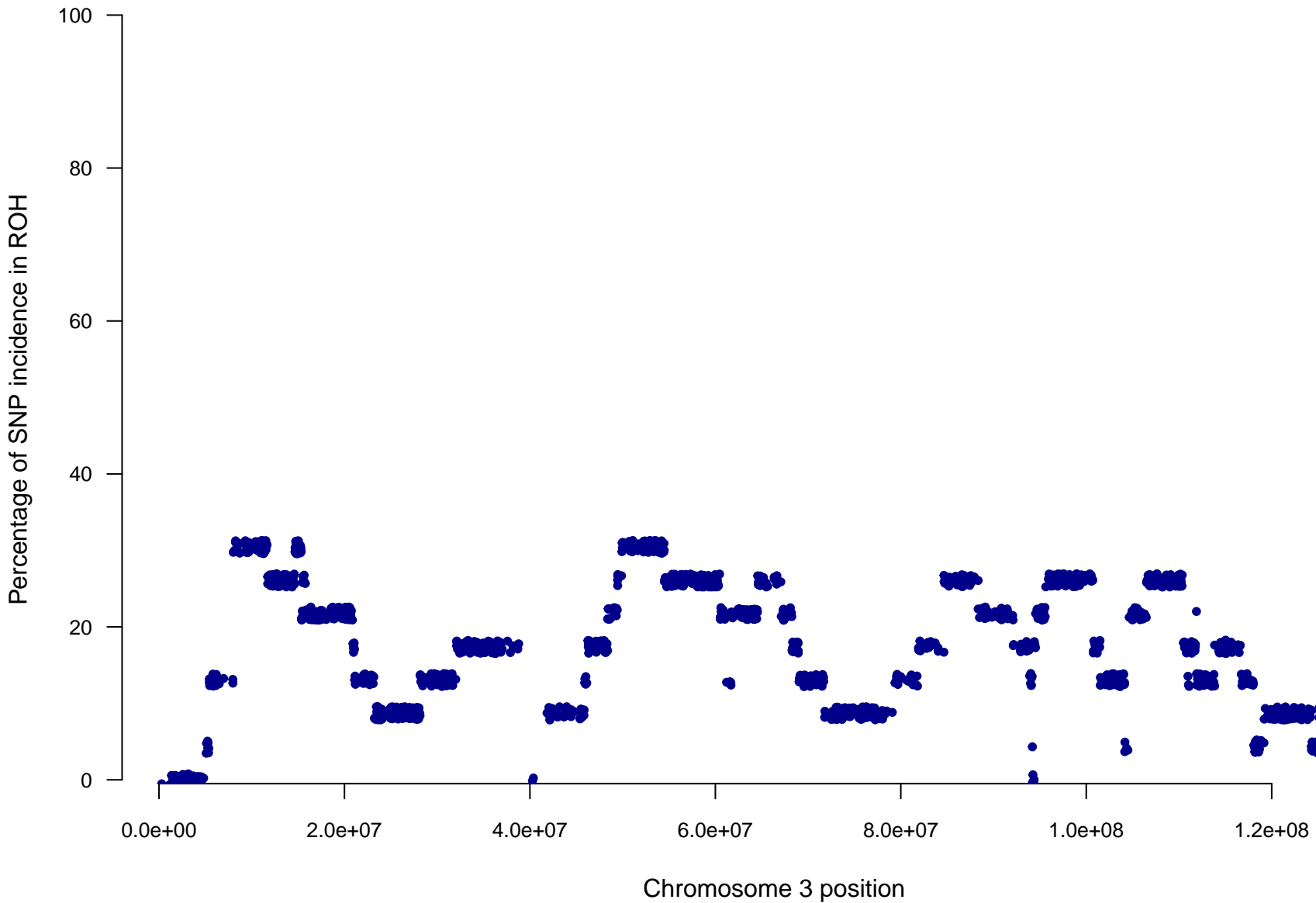

BM  
N= 23

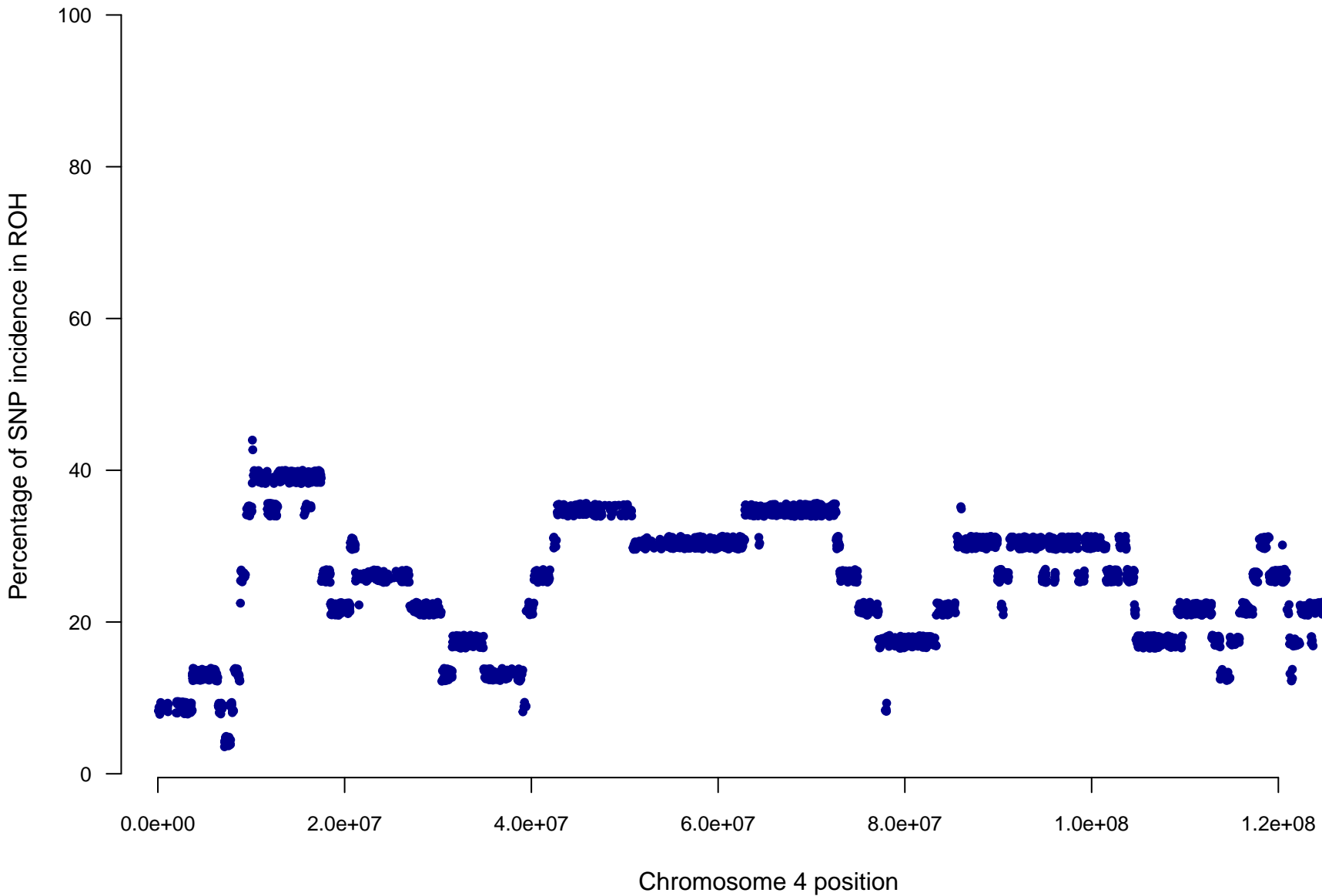

BM

N= 23

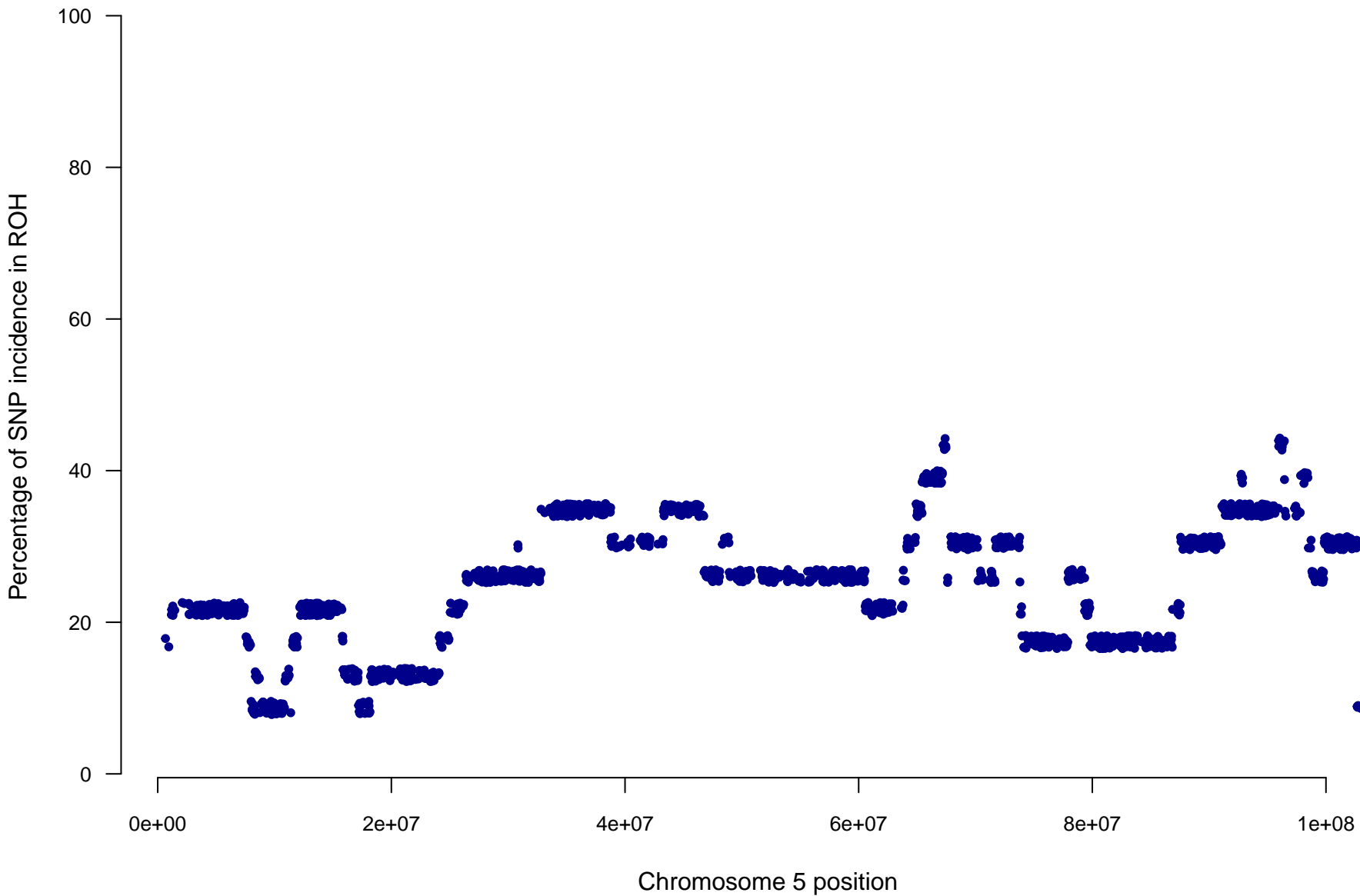

BM  
N= 23

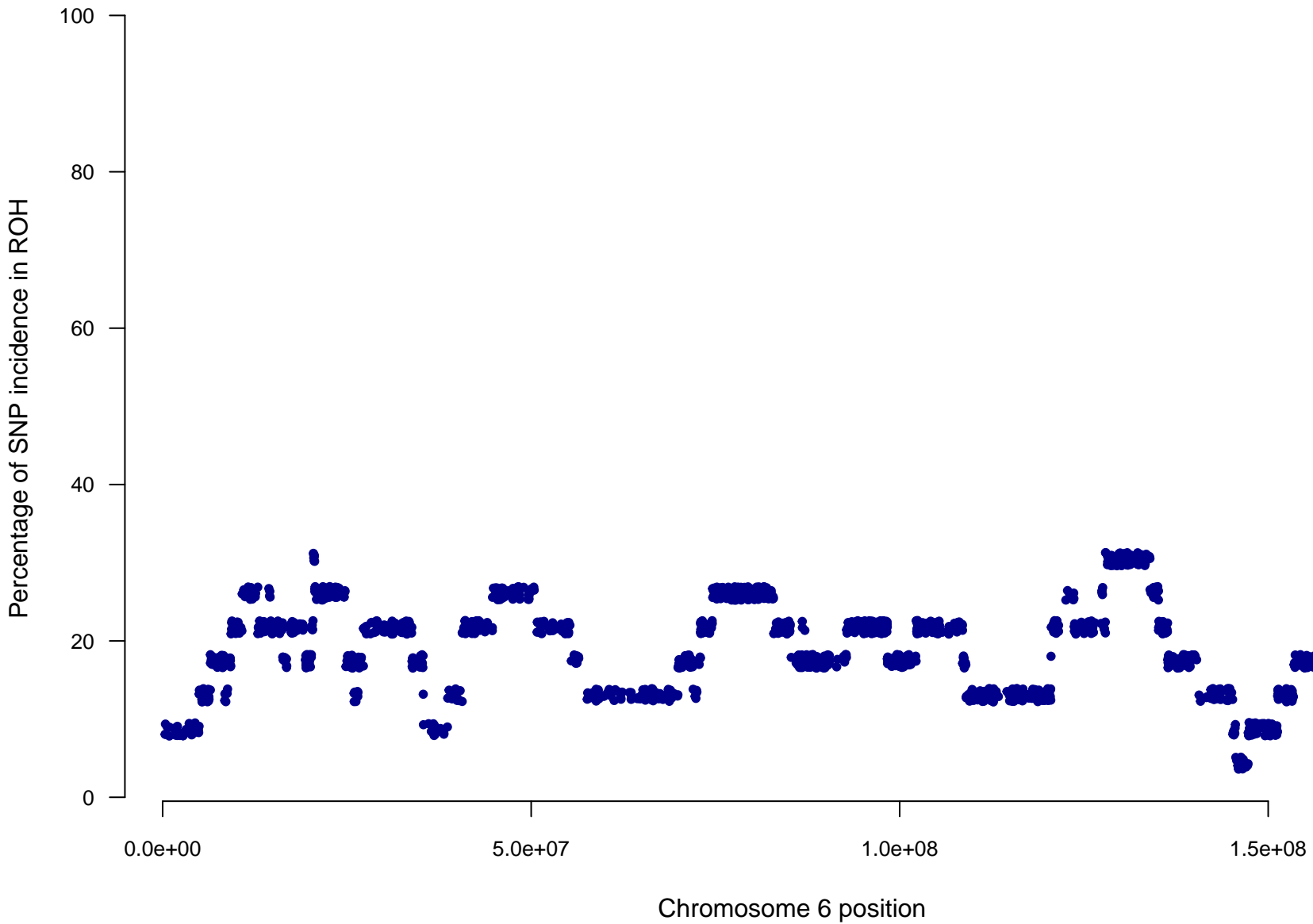

BM  
N= 23

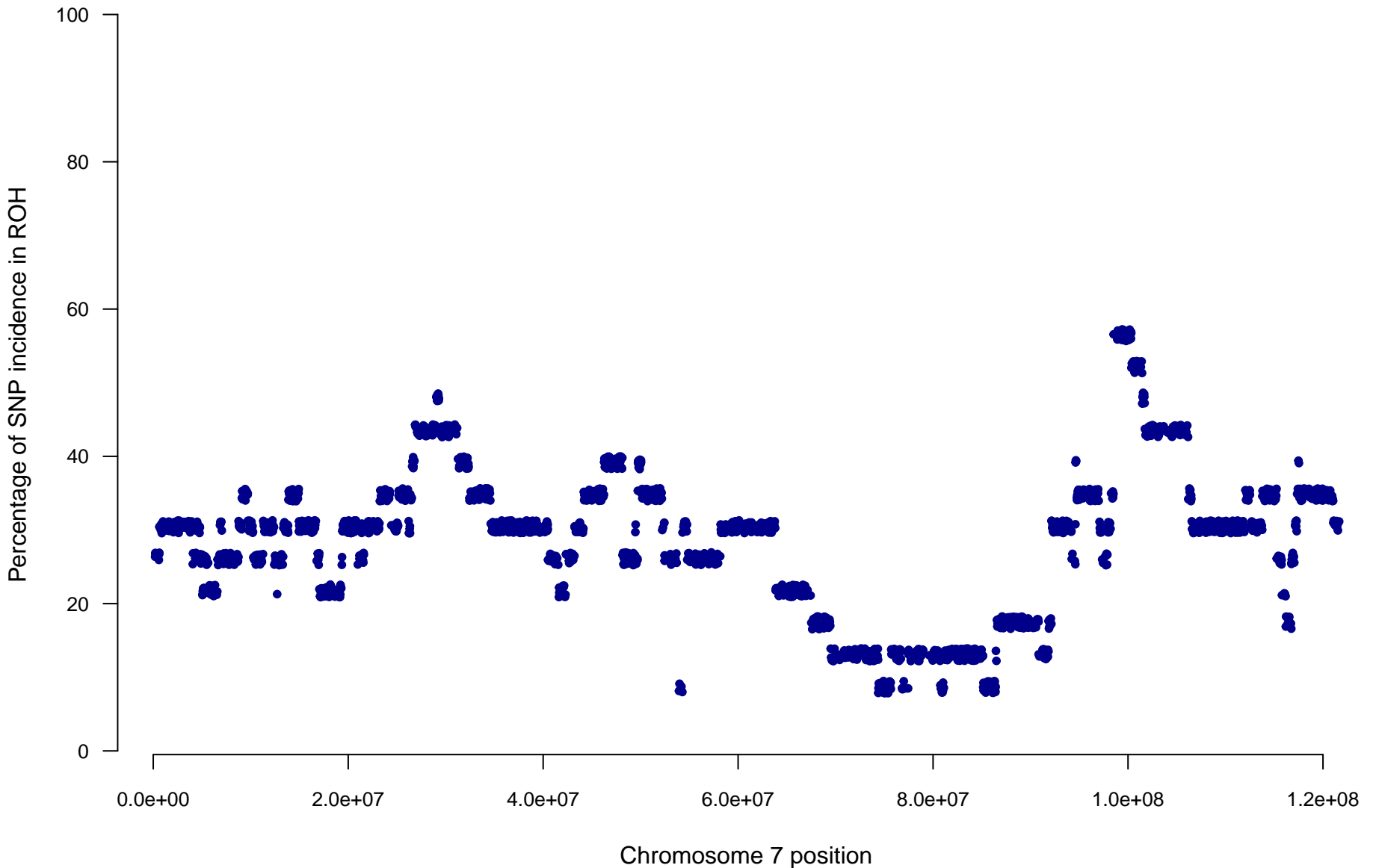

BM  
N= 23

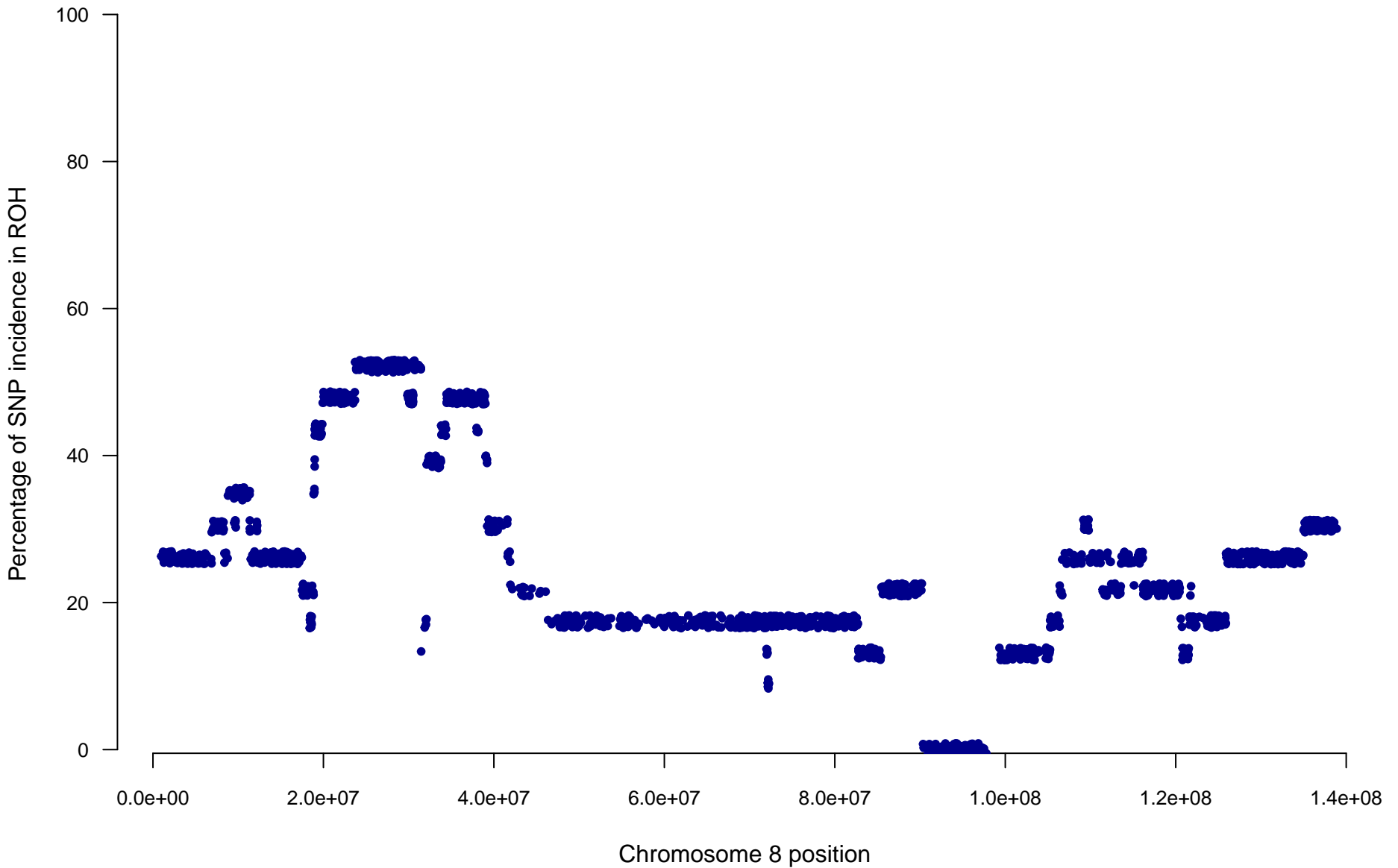

BM  
N= 23

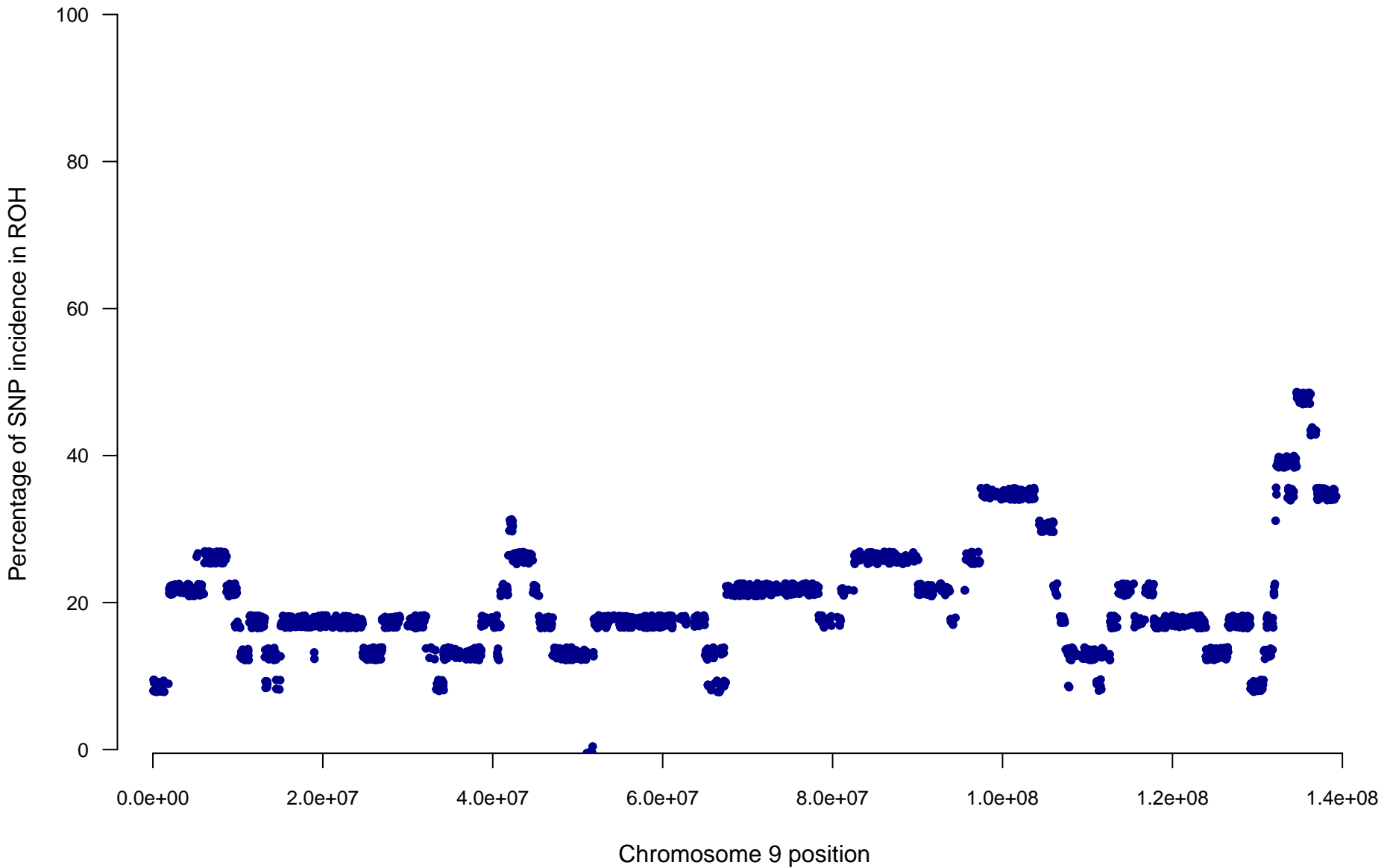

BM  
N= 23

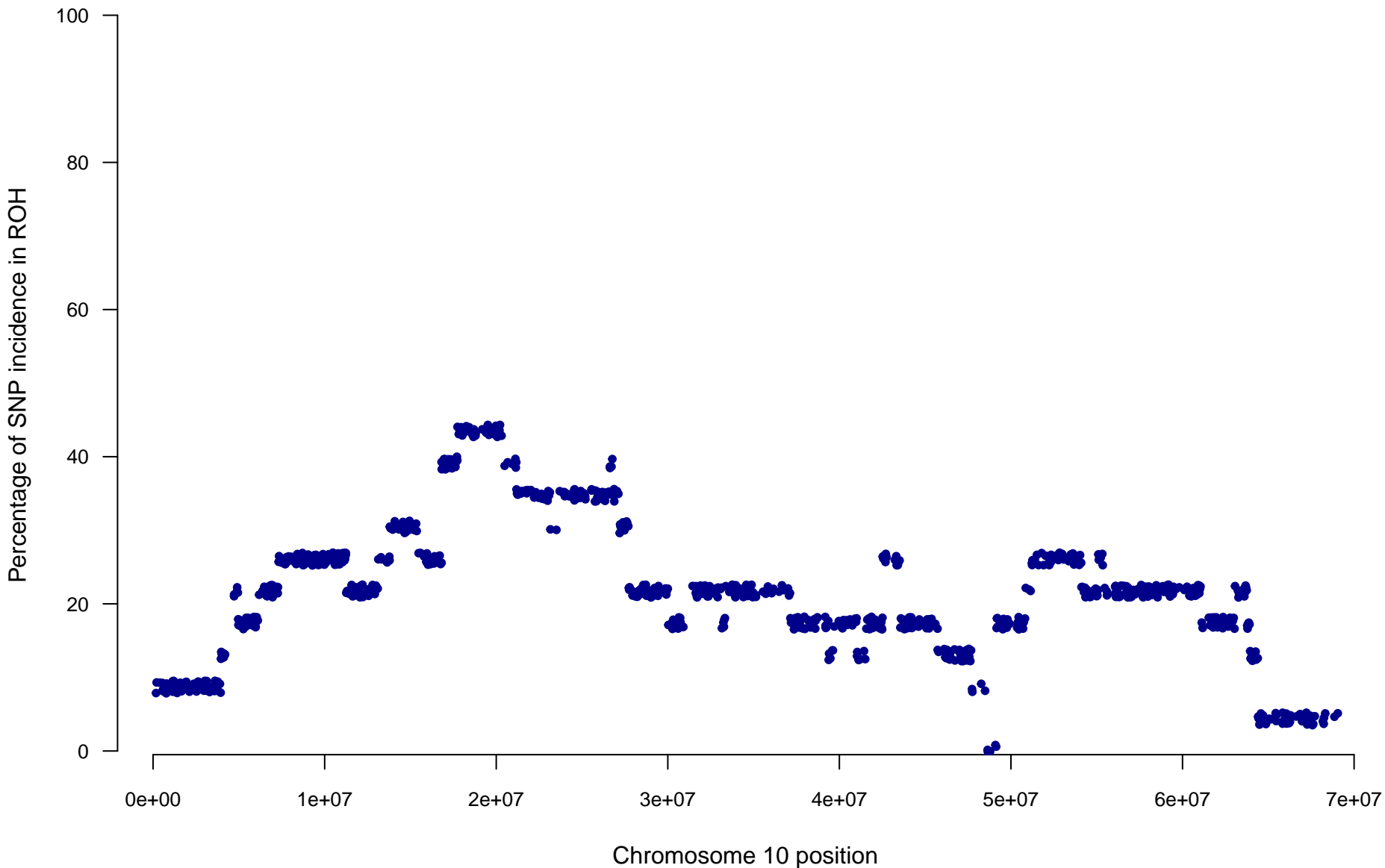

BM  
N= 23

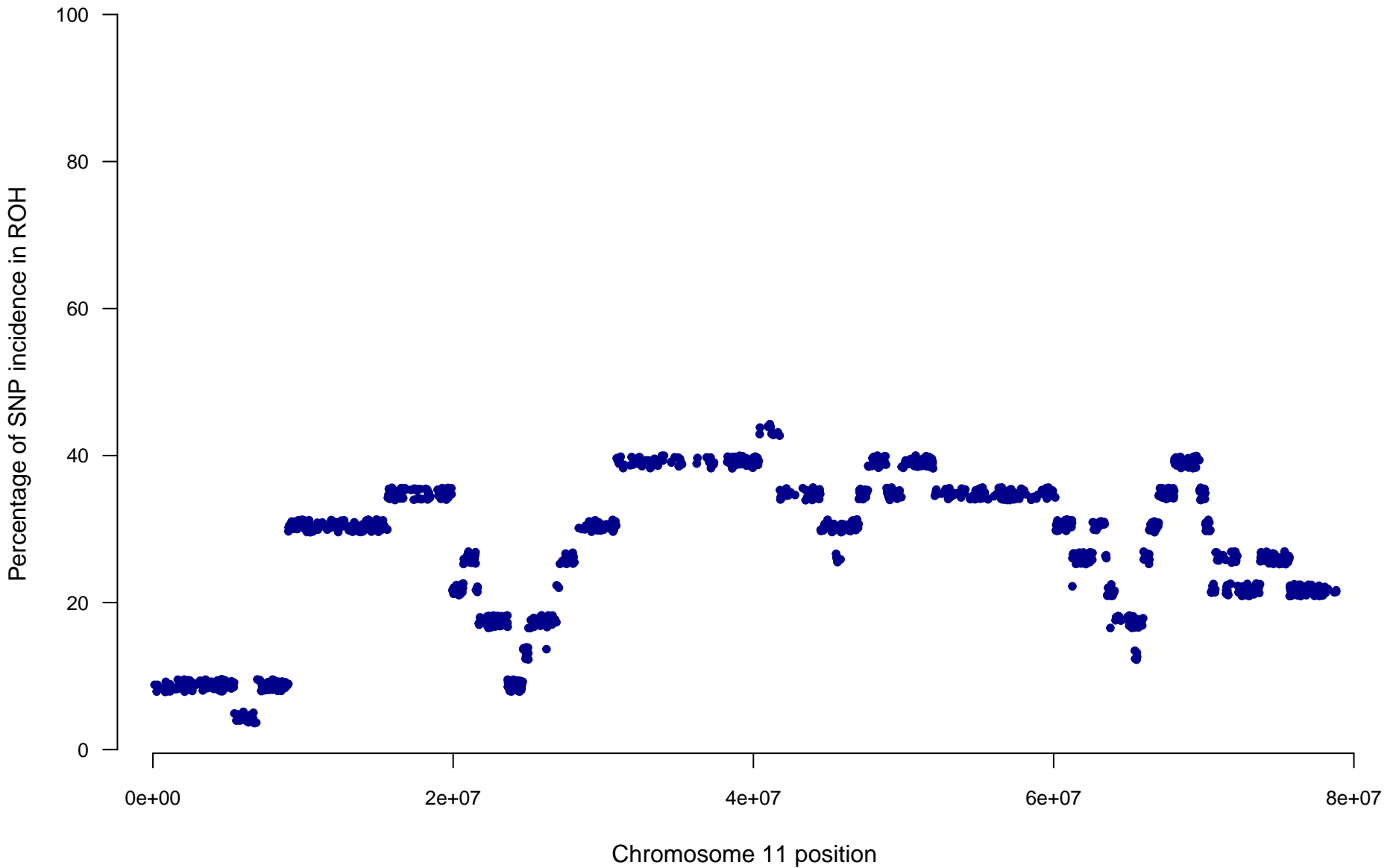

BM  
N= 23

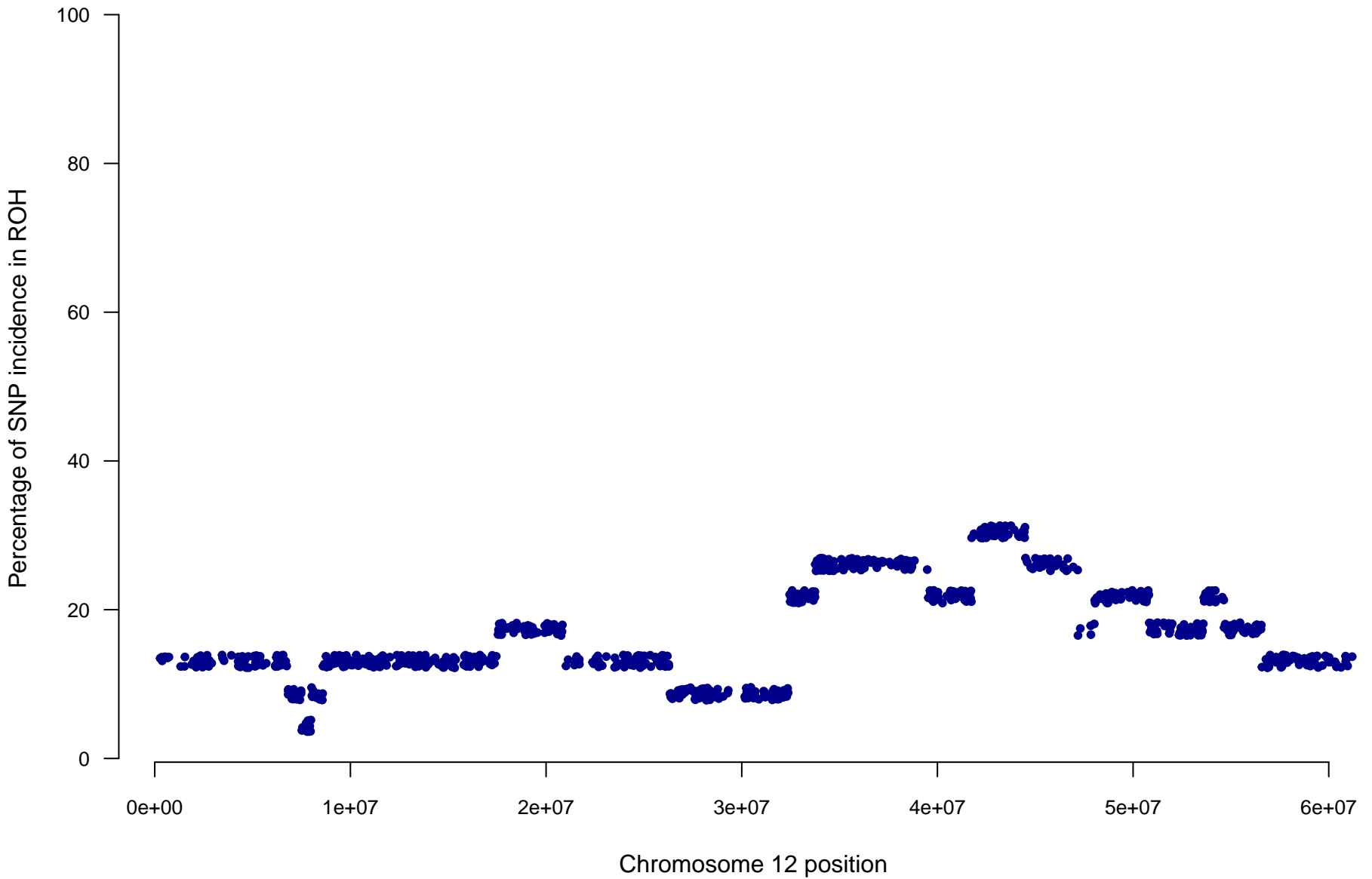

BM  
N= 23

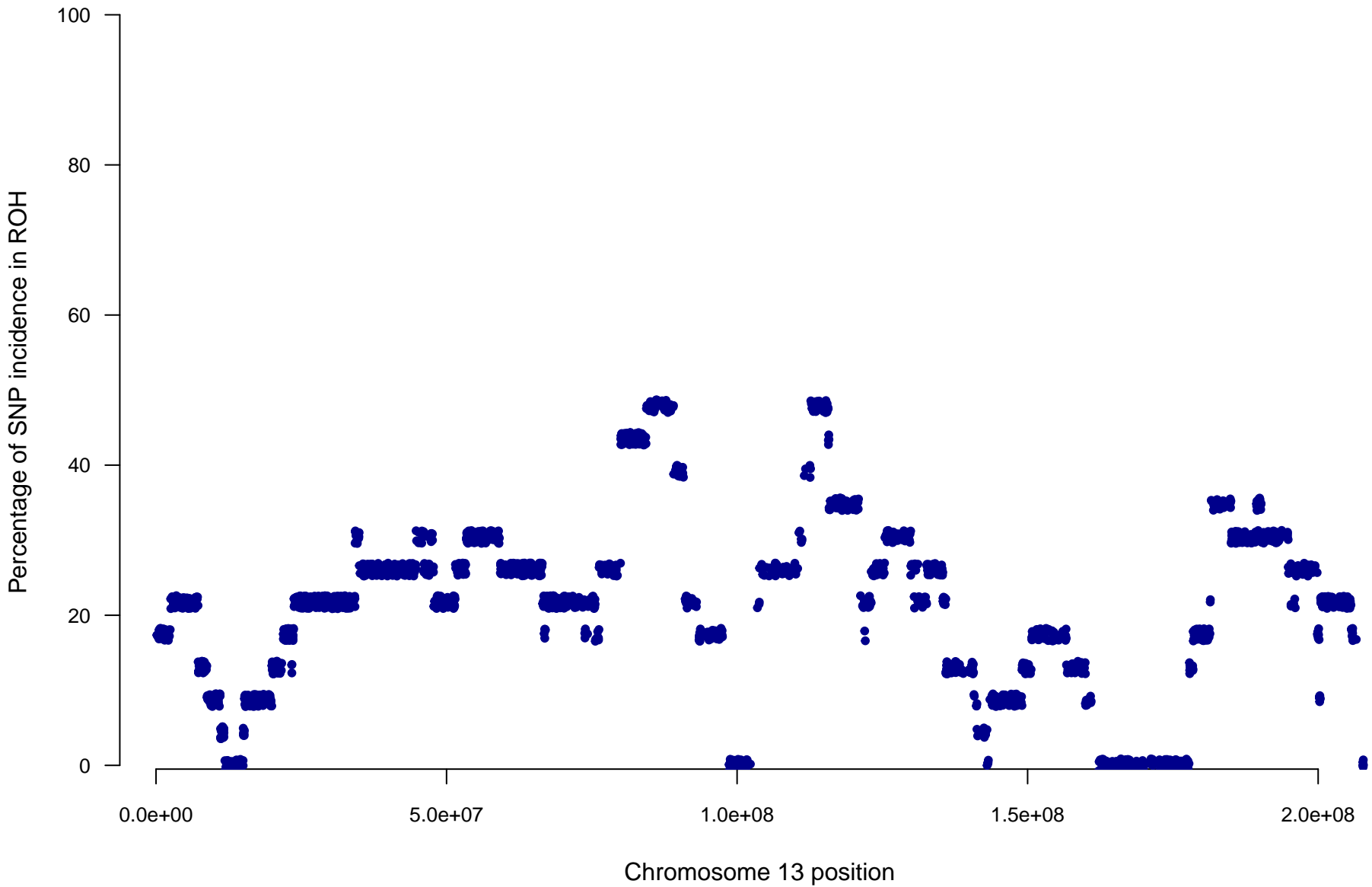

BM  
N= 23

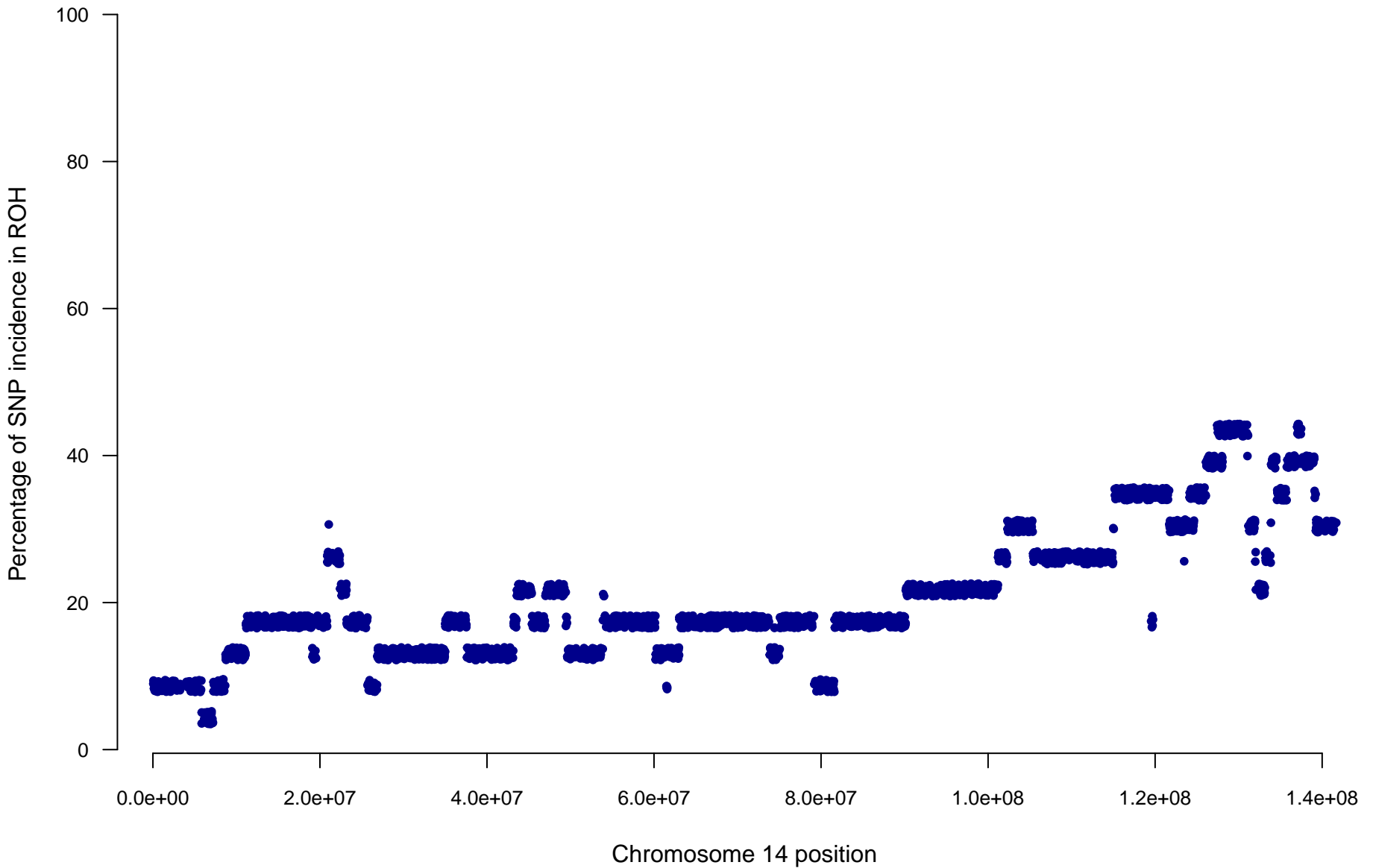

BM  
N= 23

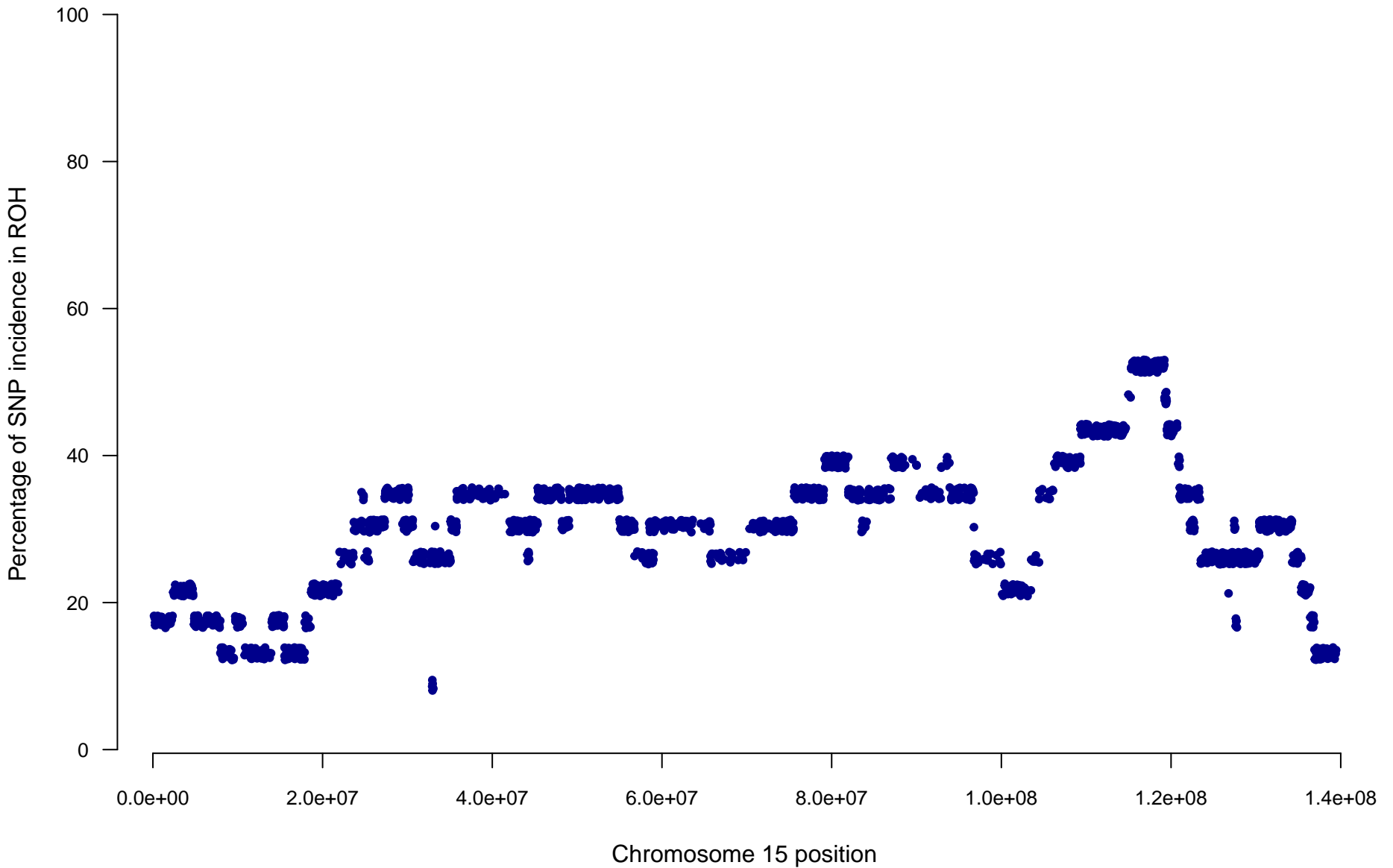

BM  
N= 23

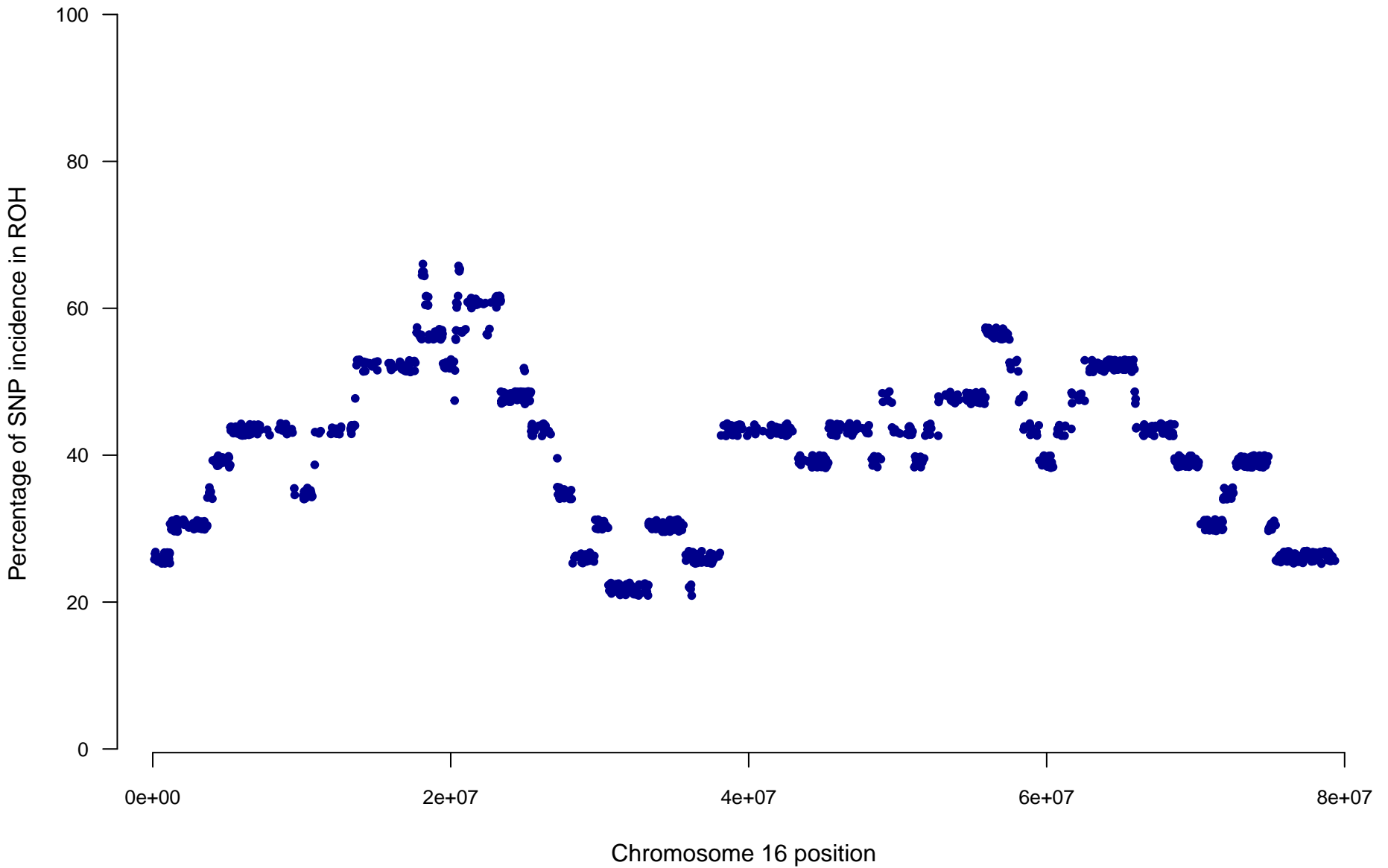

BM  
N= 23

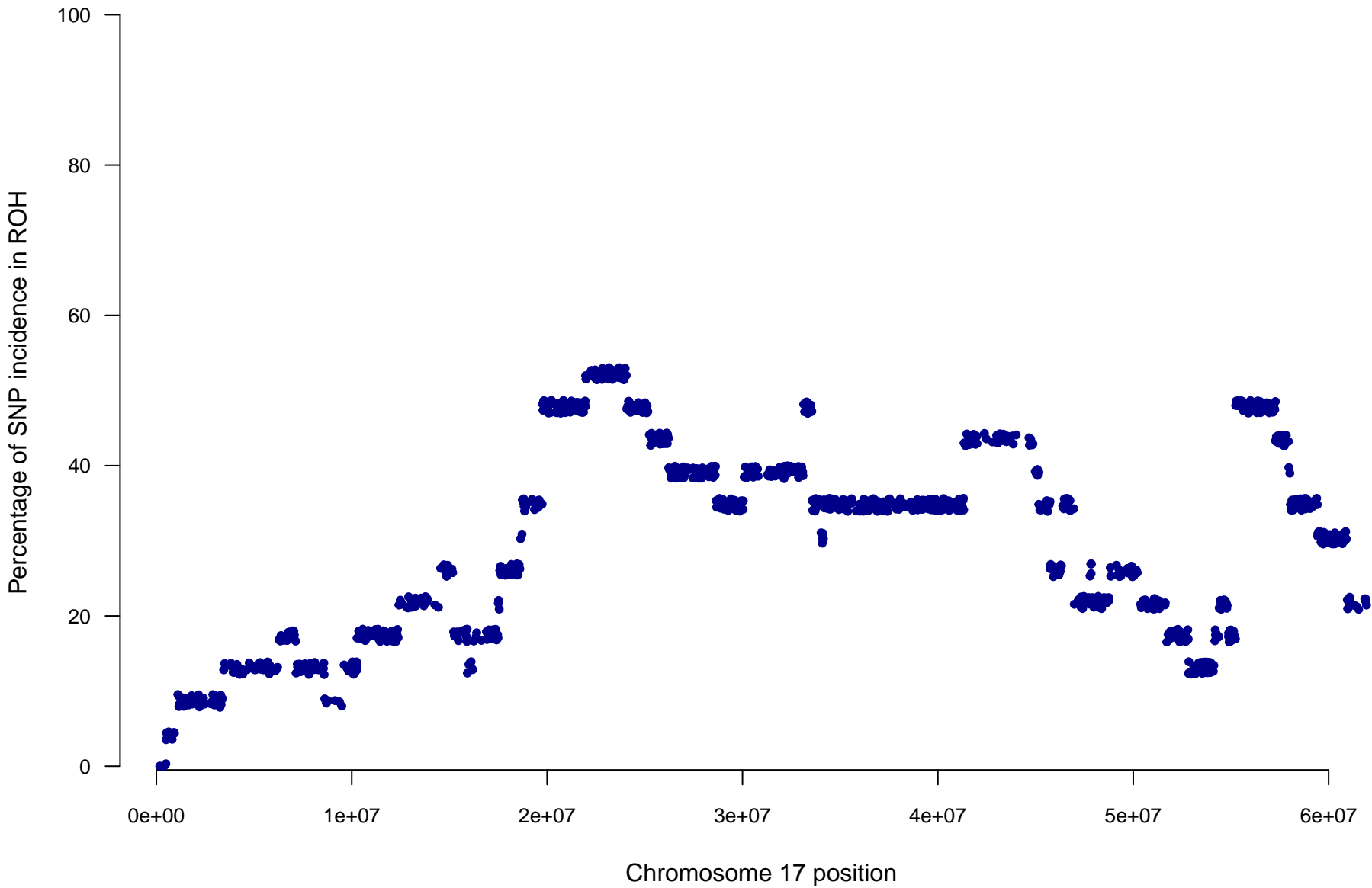

BM  
N= 23

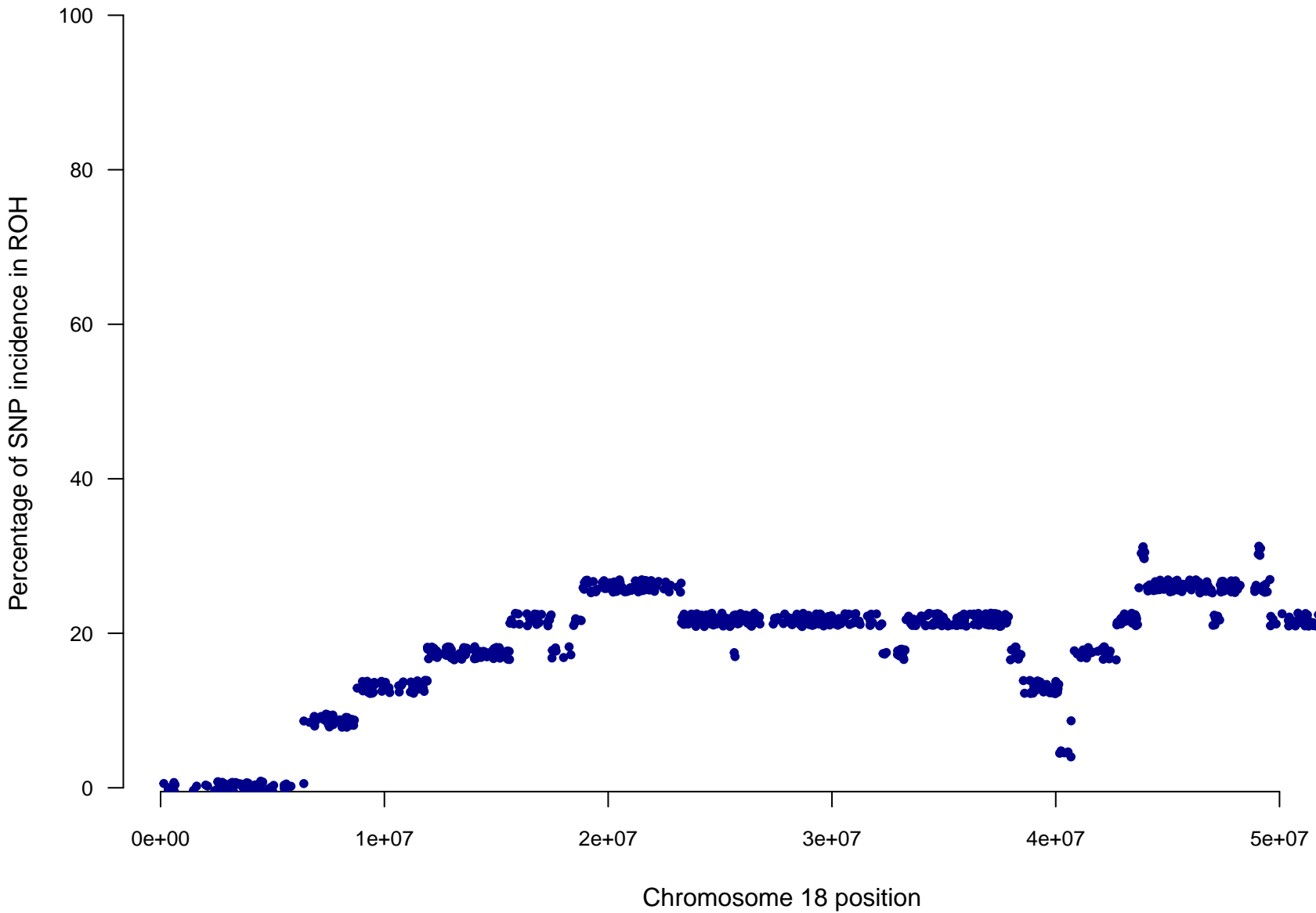

Supplement: Supplementary file 5 [file Image2.pdf]

RM

N= 24

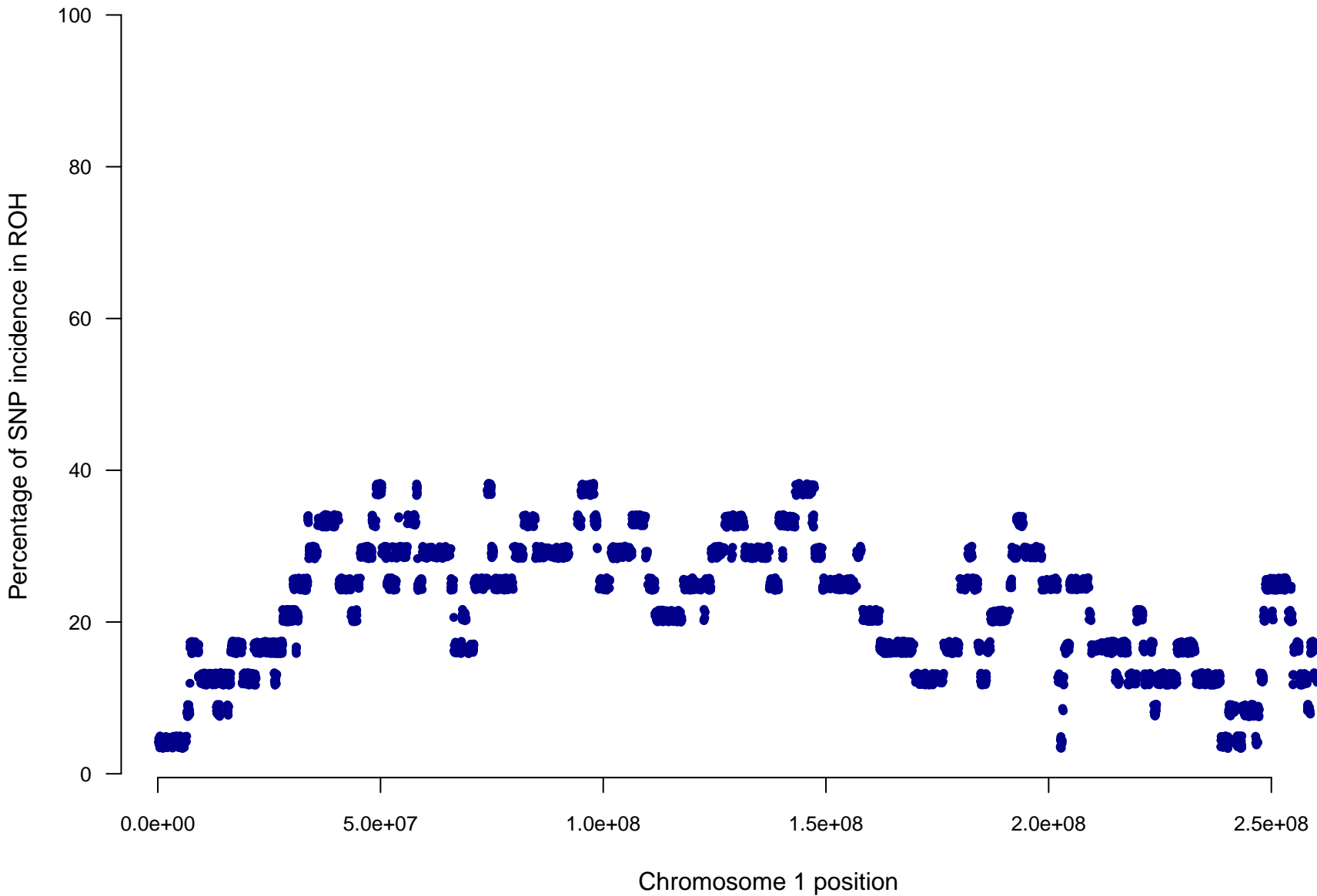

RM  
N= 24

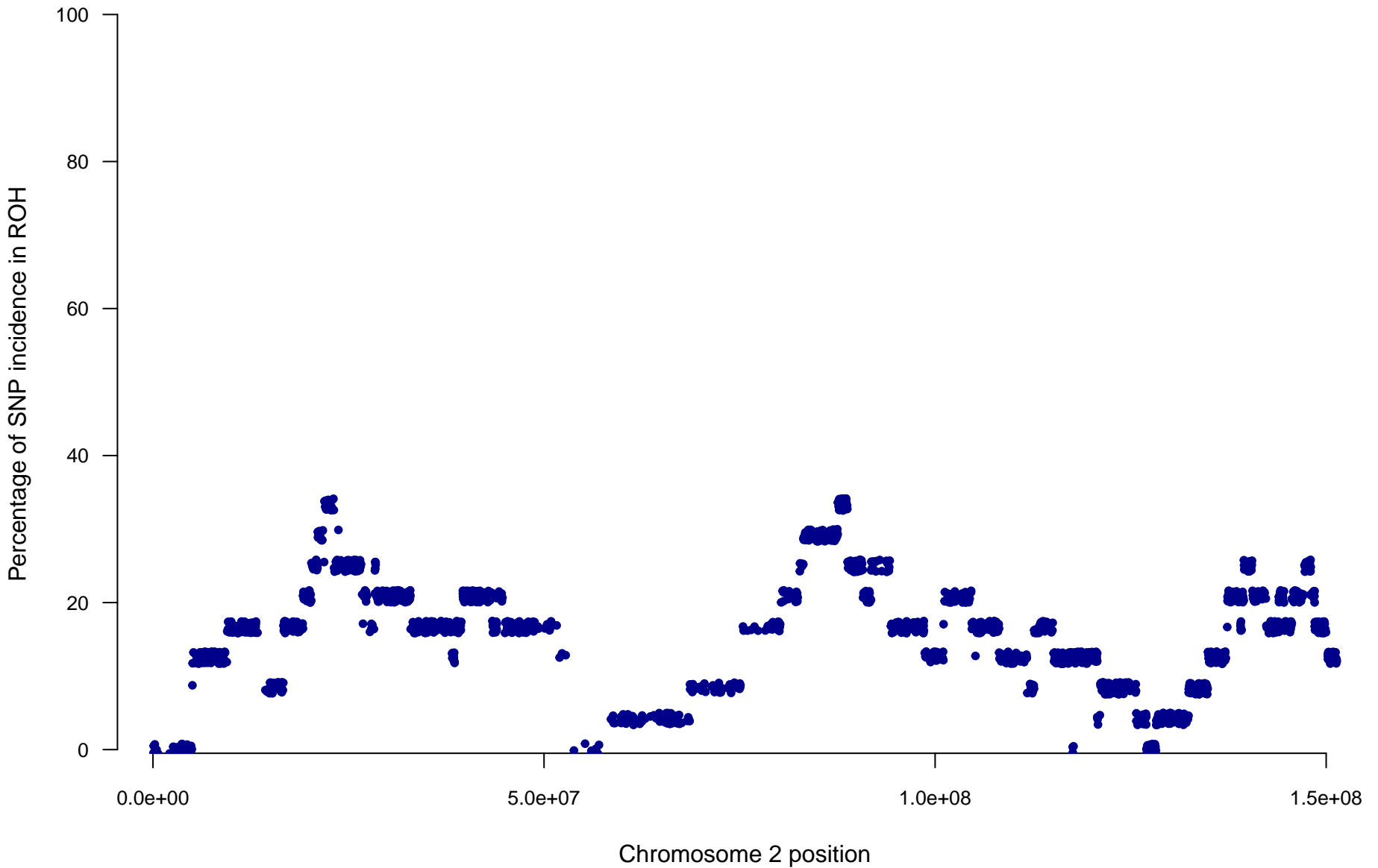

RM  
N= 24

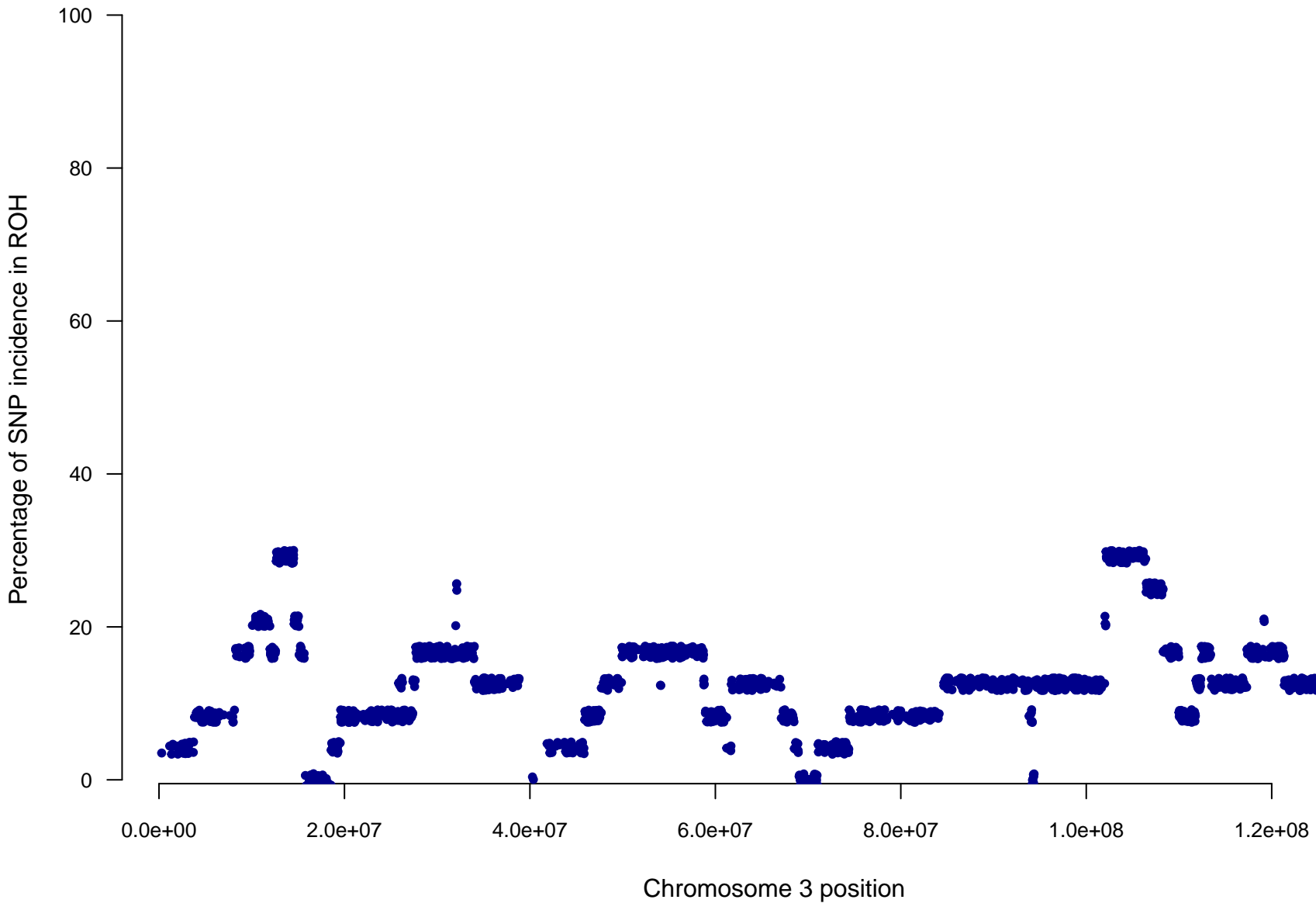

RM  
N= 24

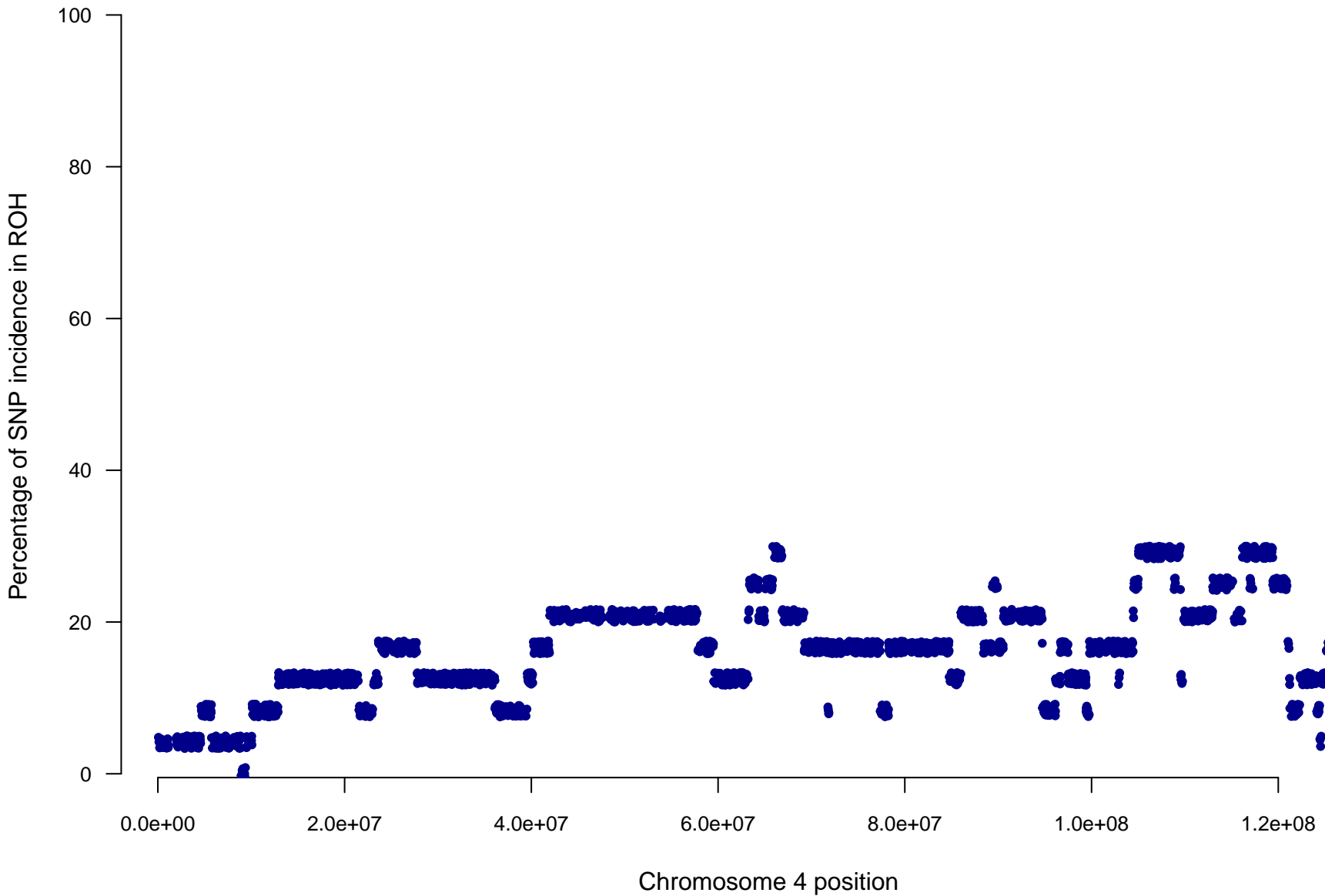

RM  
N= 24

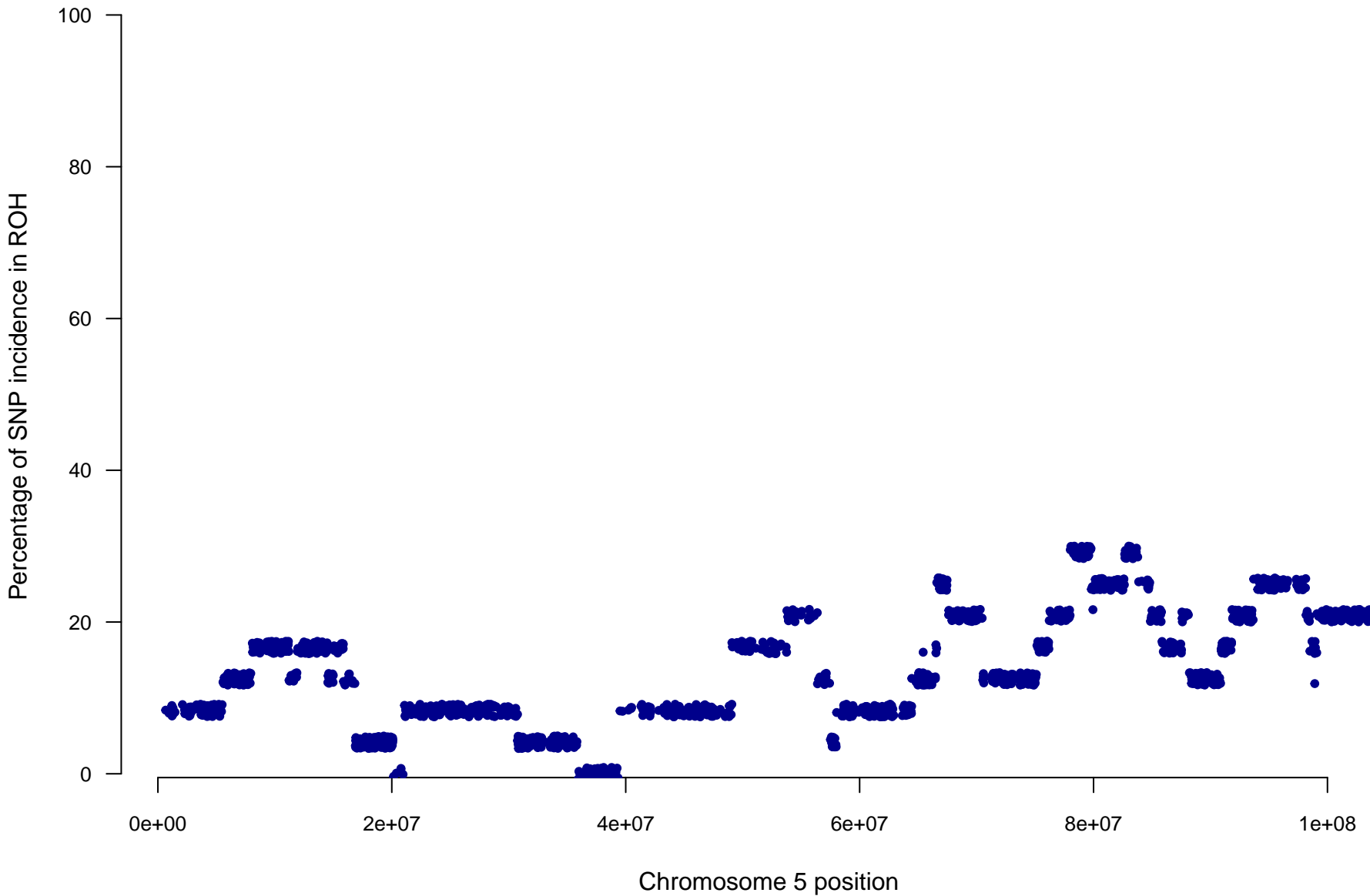

RM  
N= 24

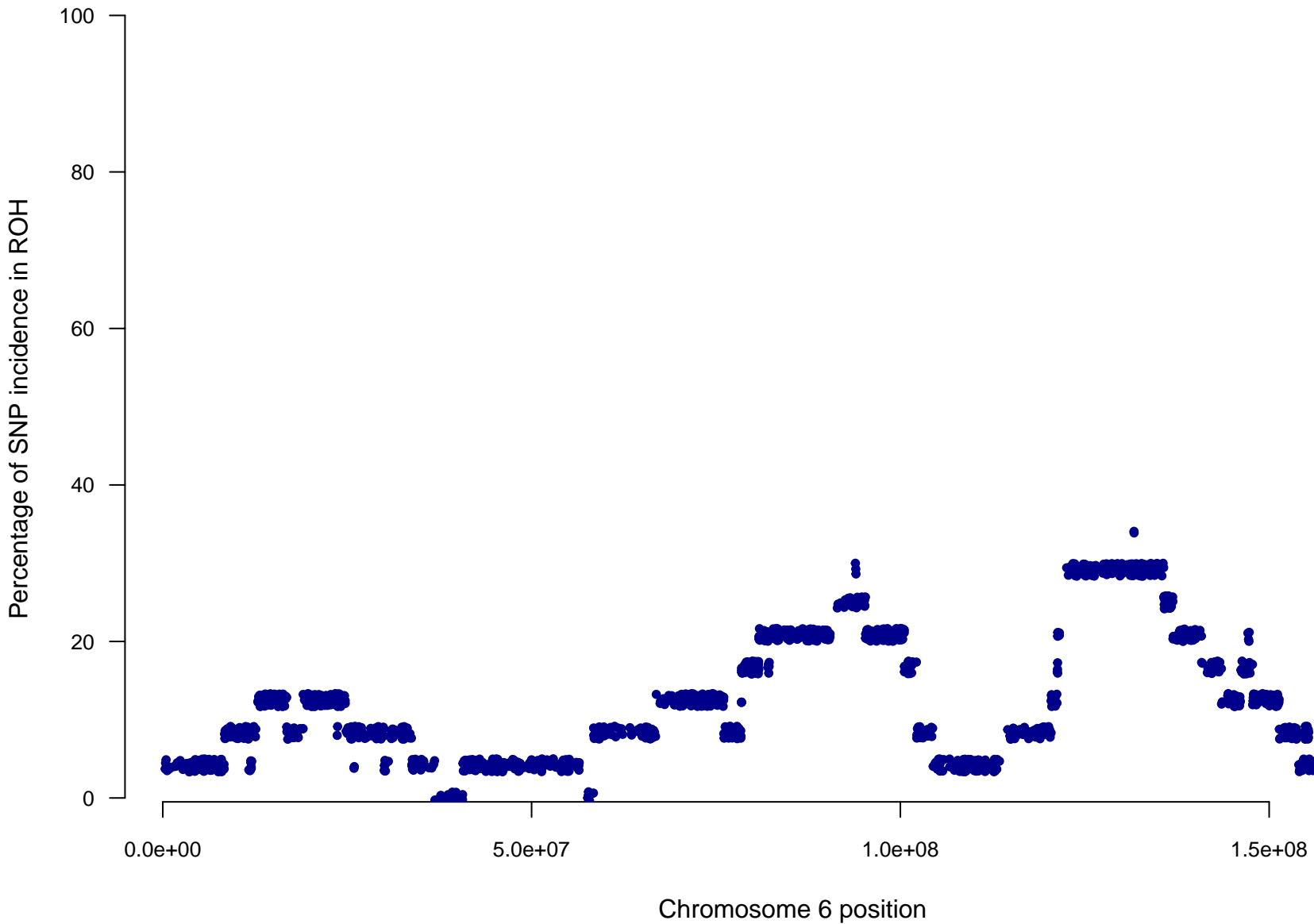

RM  
N= 24

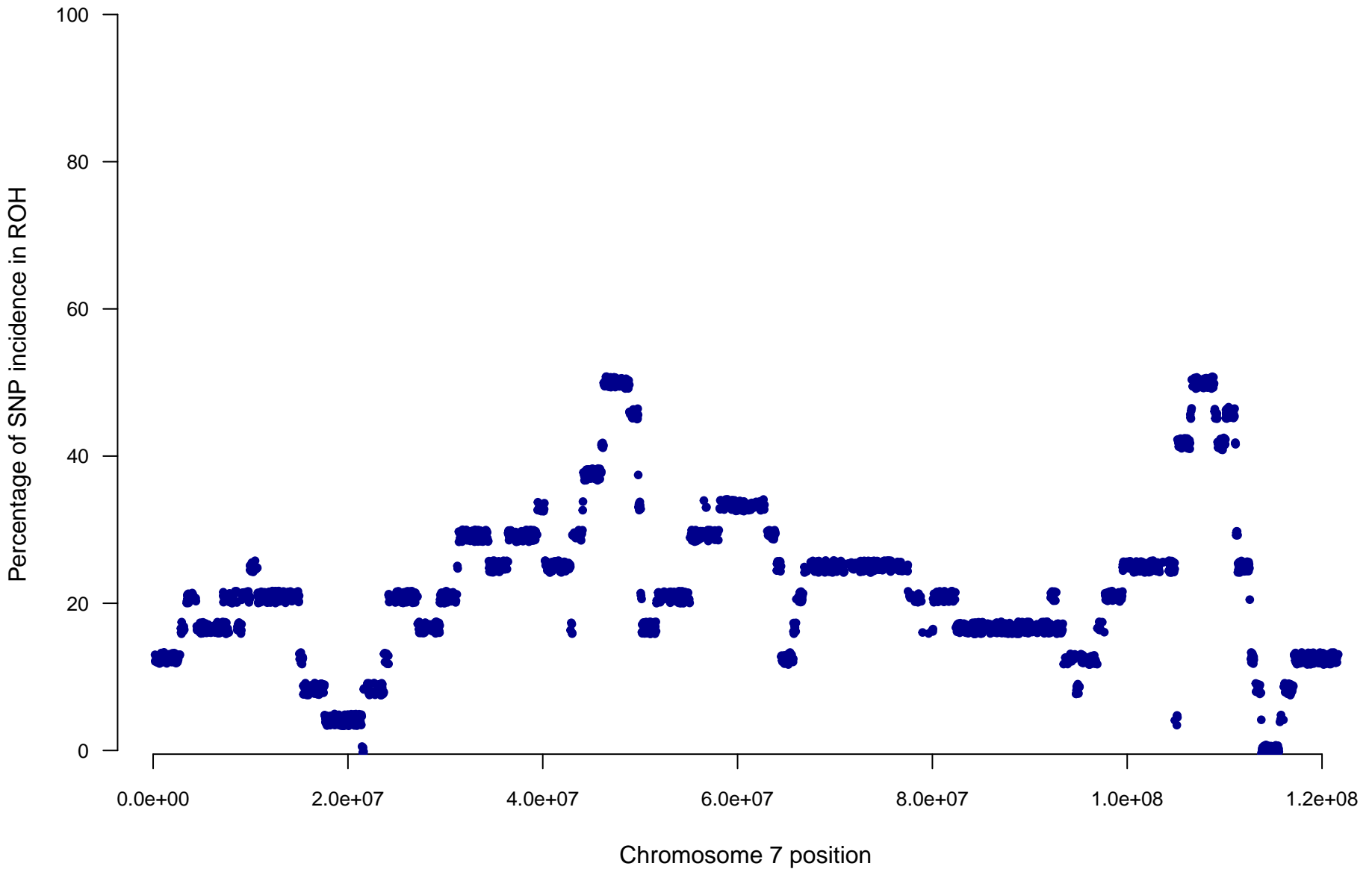

RM  
N= 24

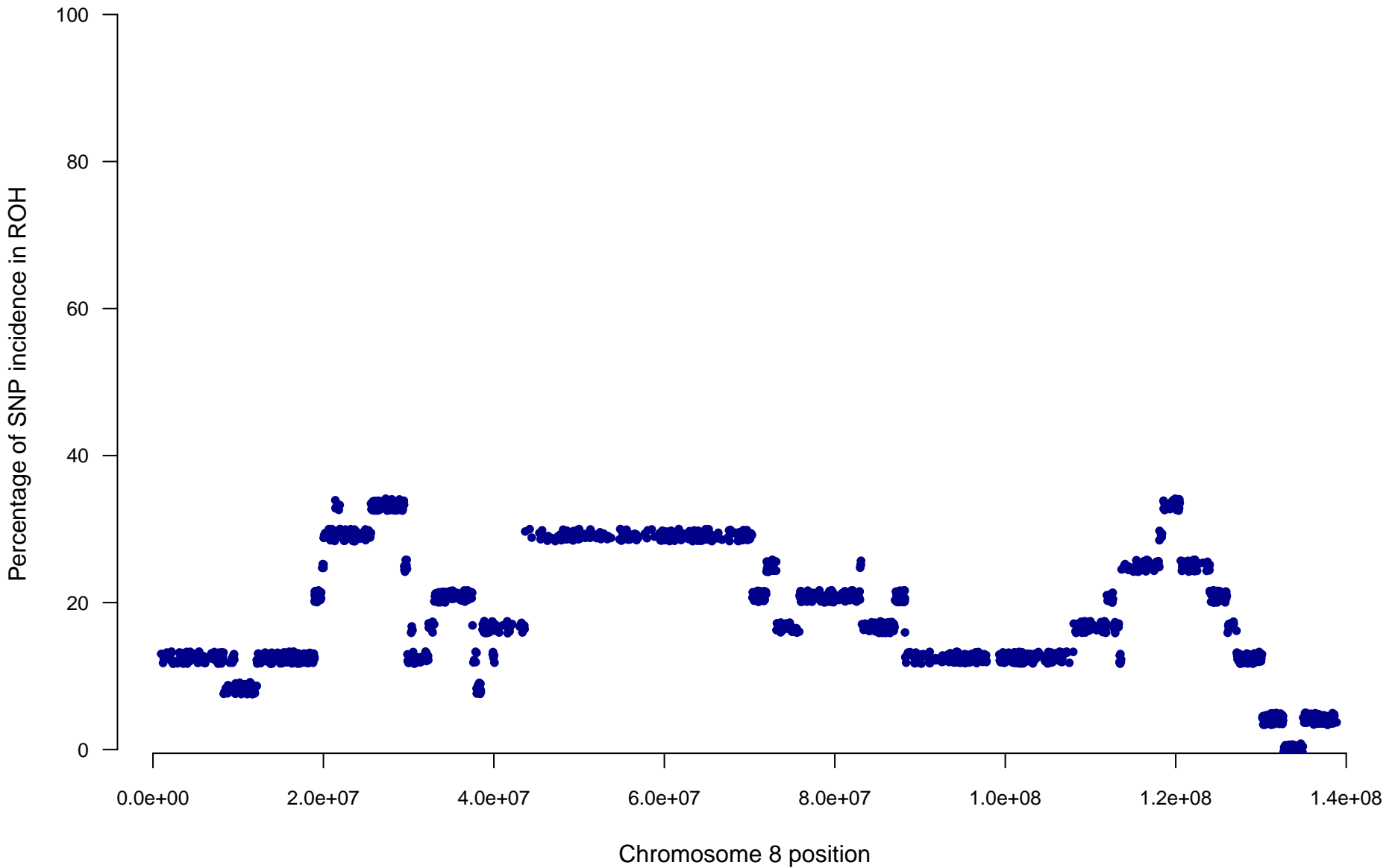

RM  
N= 24

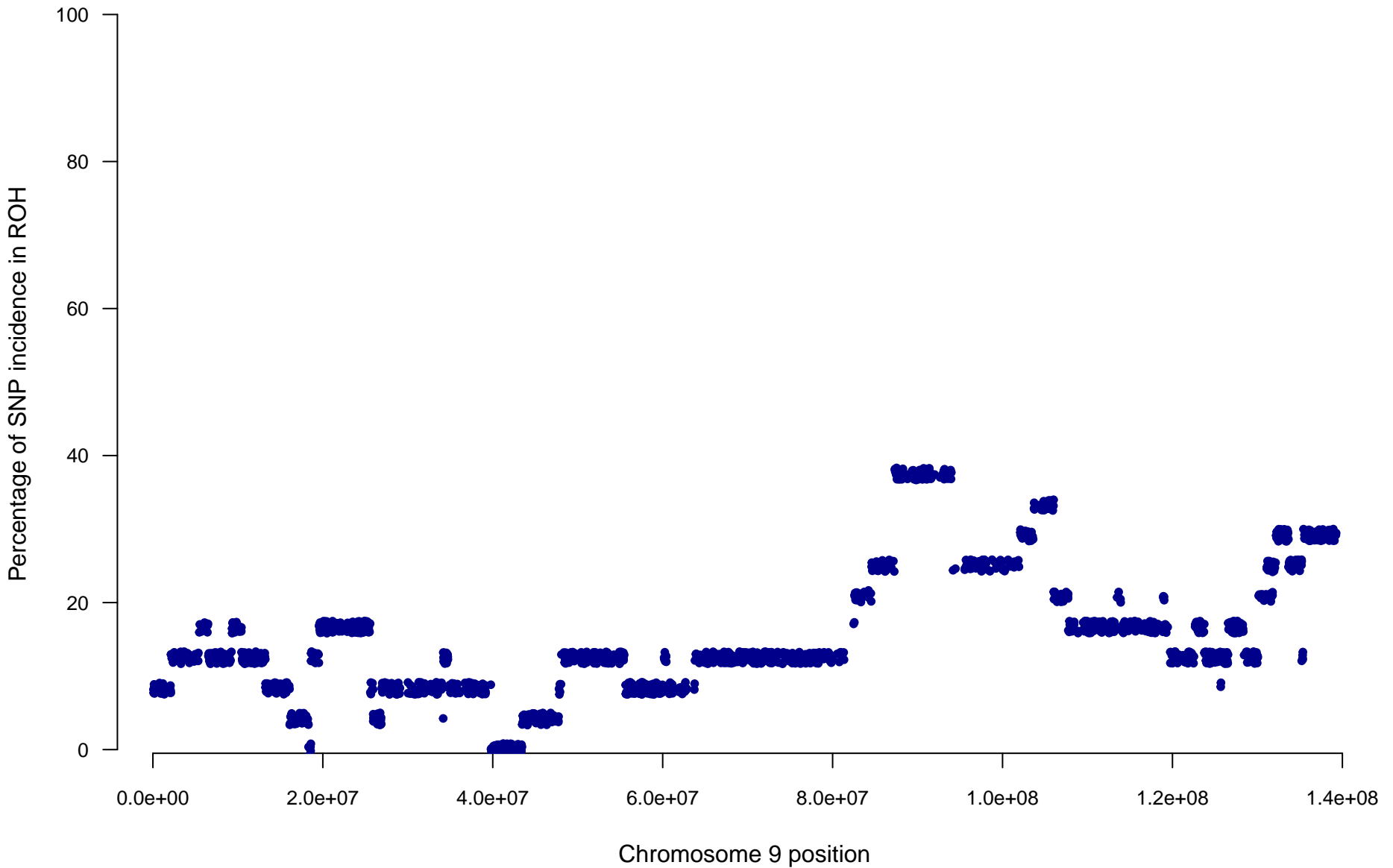

RM  
N= 24

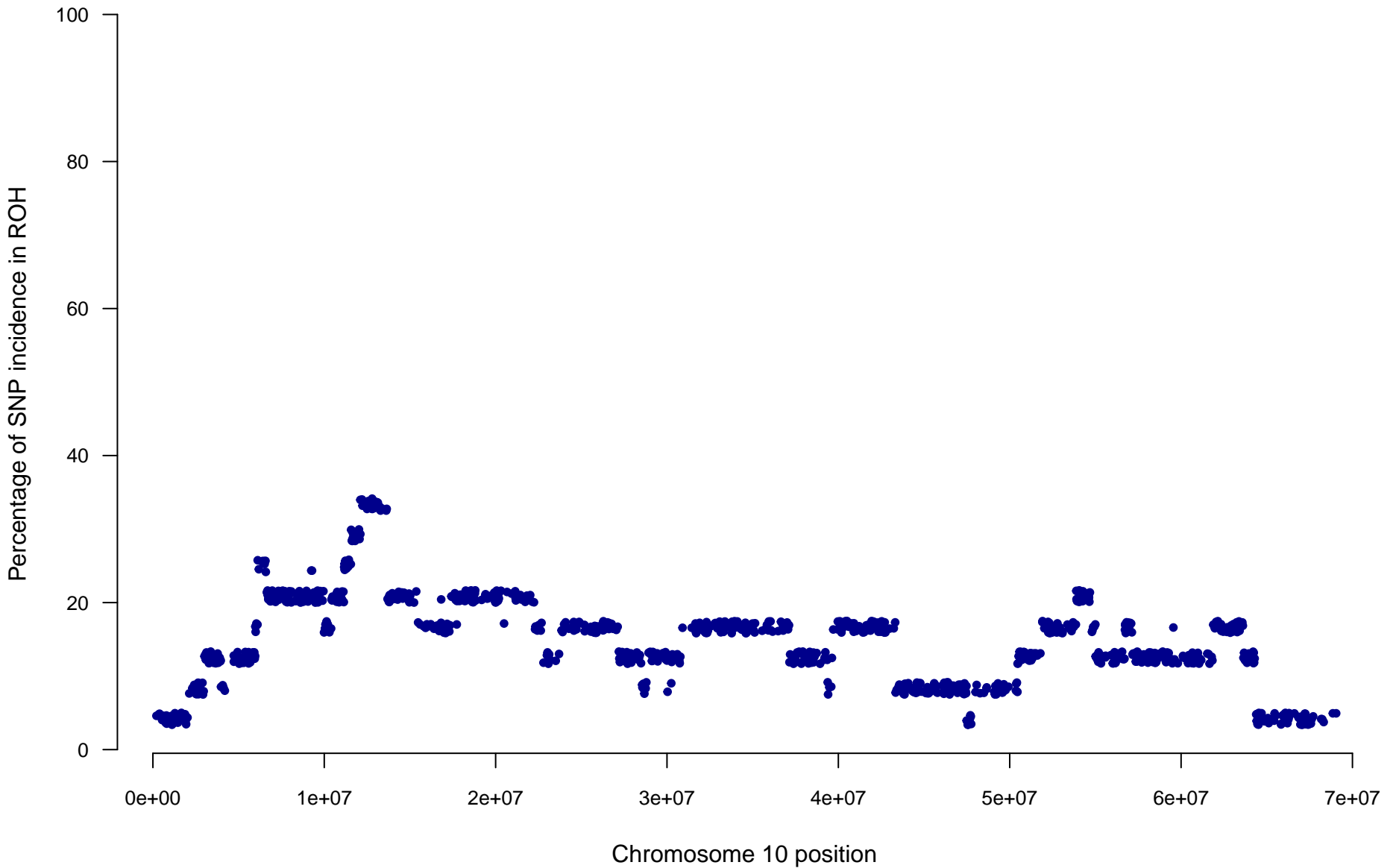

RM  
N= 24

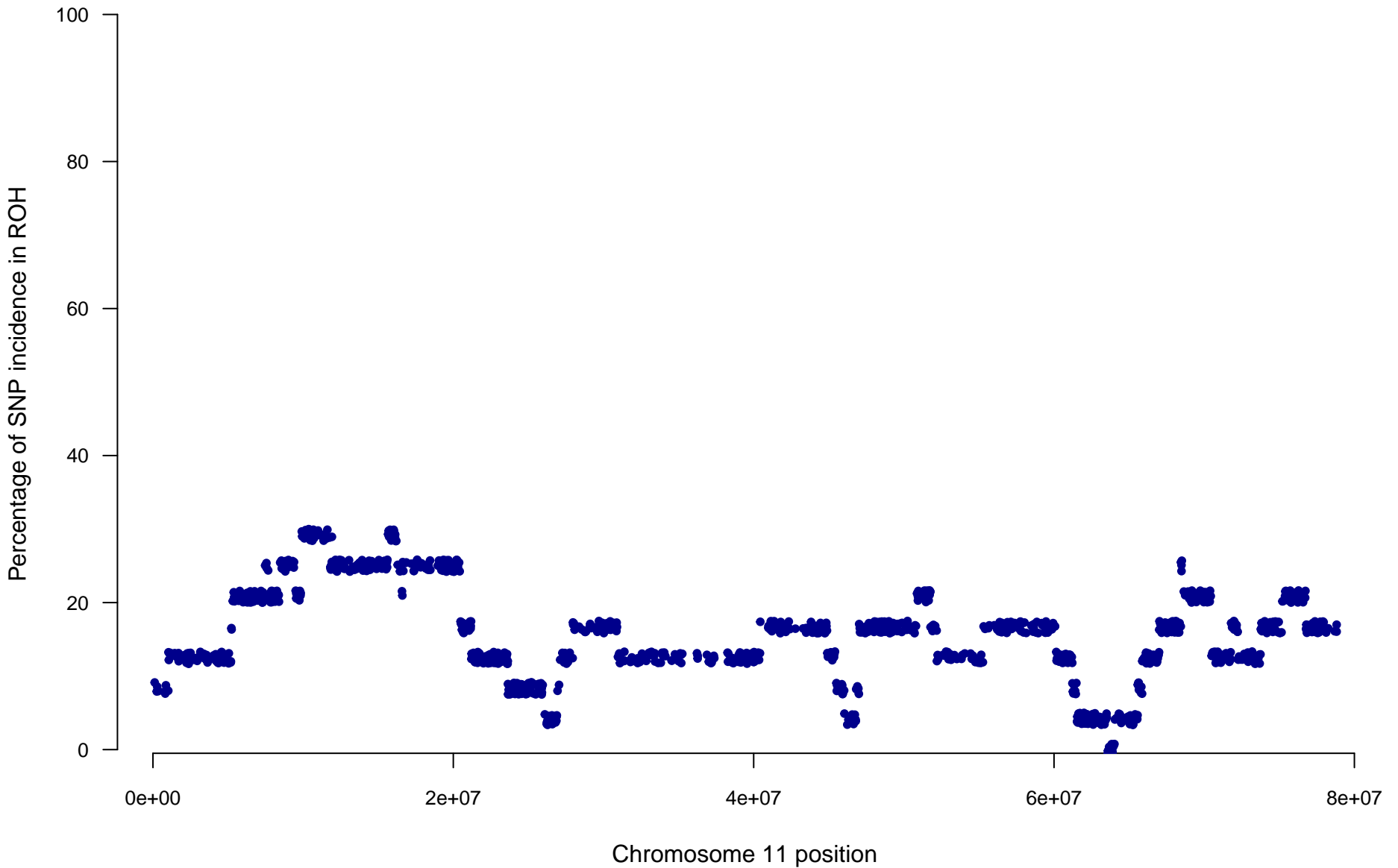

RM  
N= 24

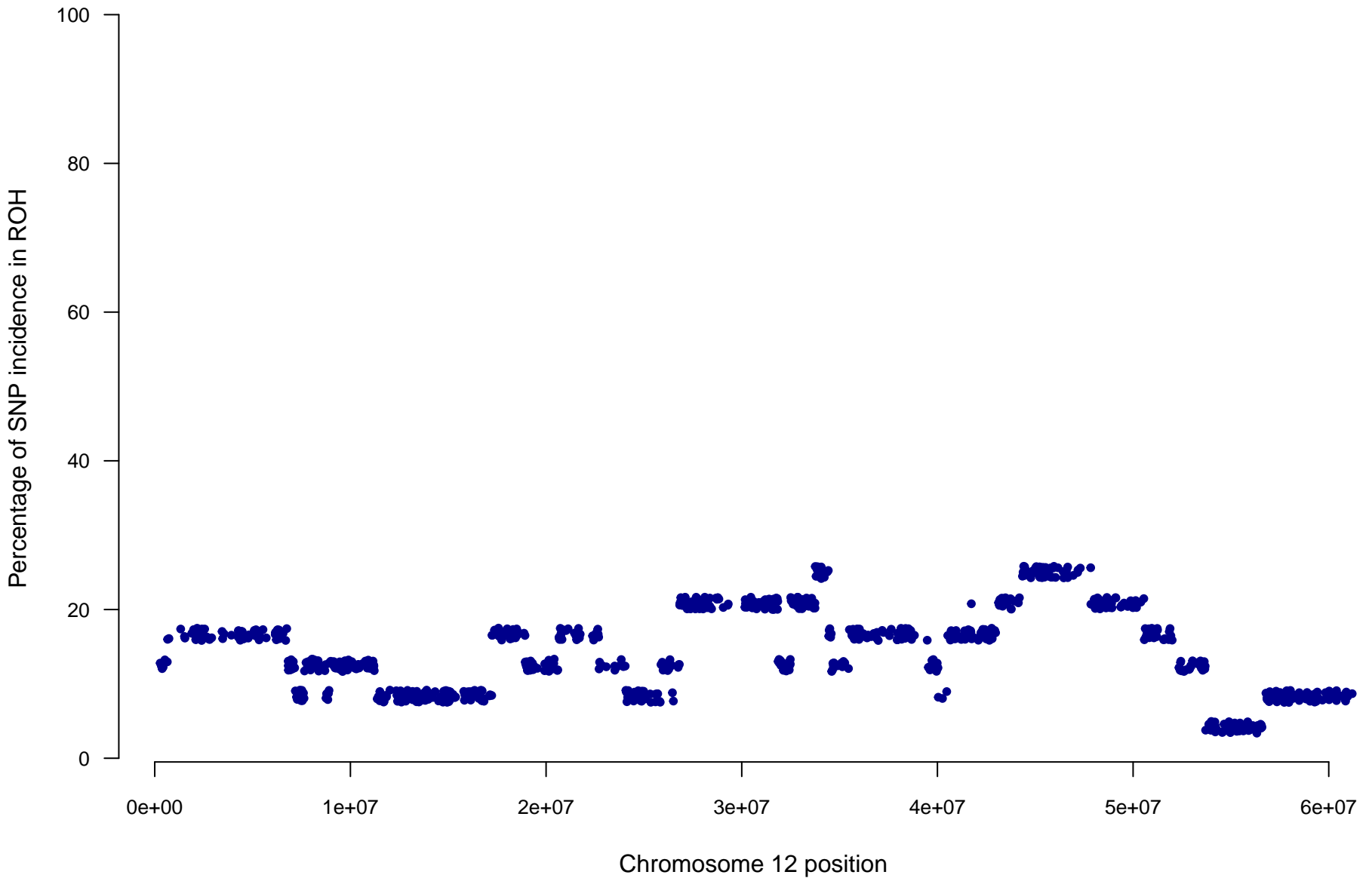

RM  
N= 24

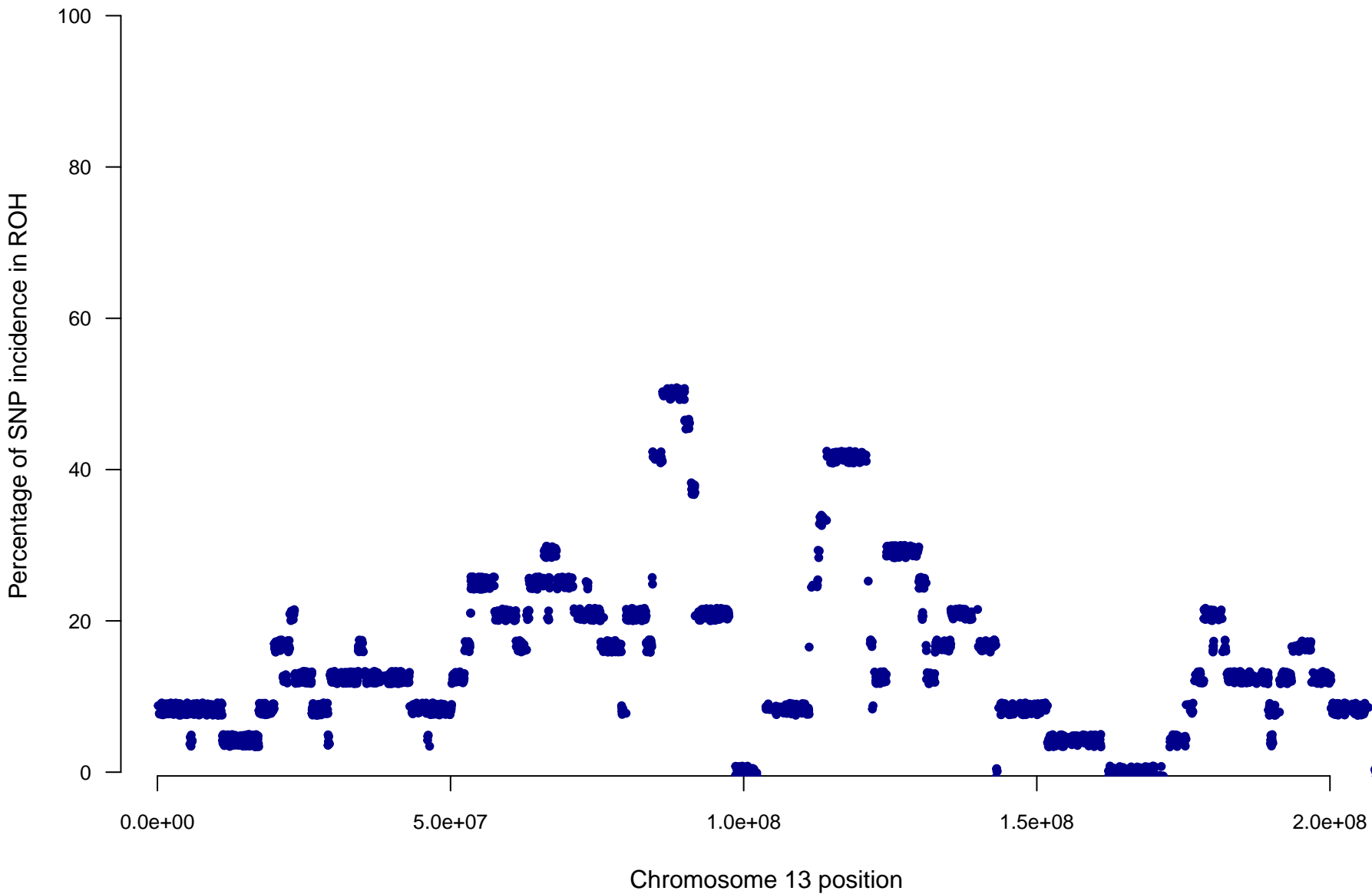

RM  
N= 24

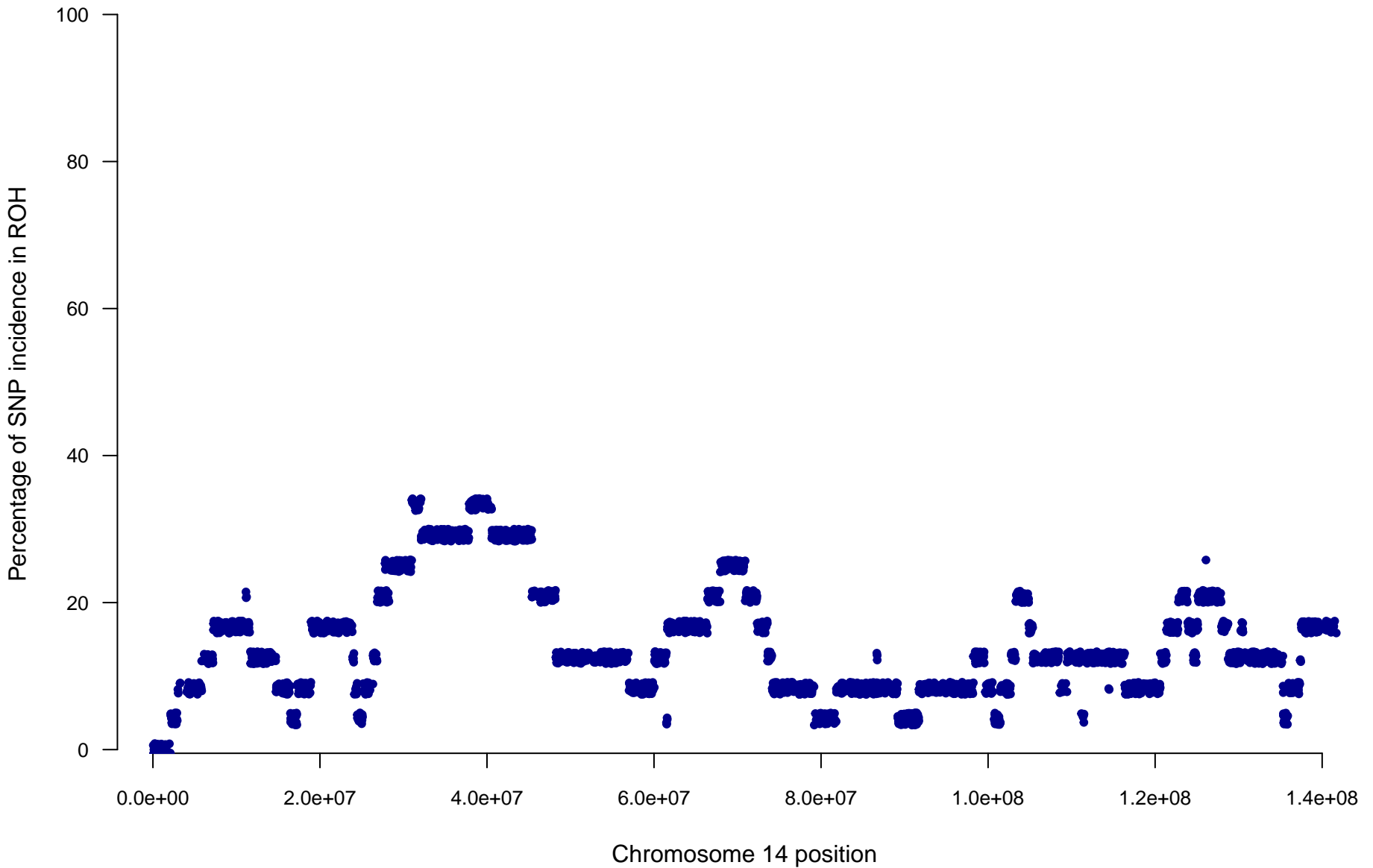

RM  
N= 24

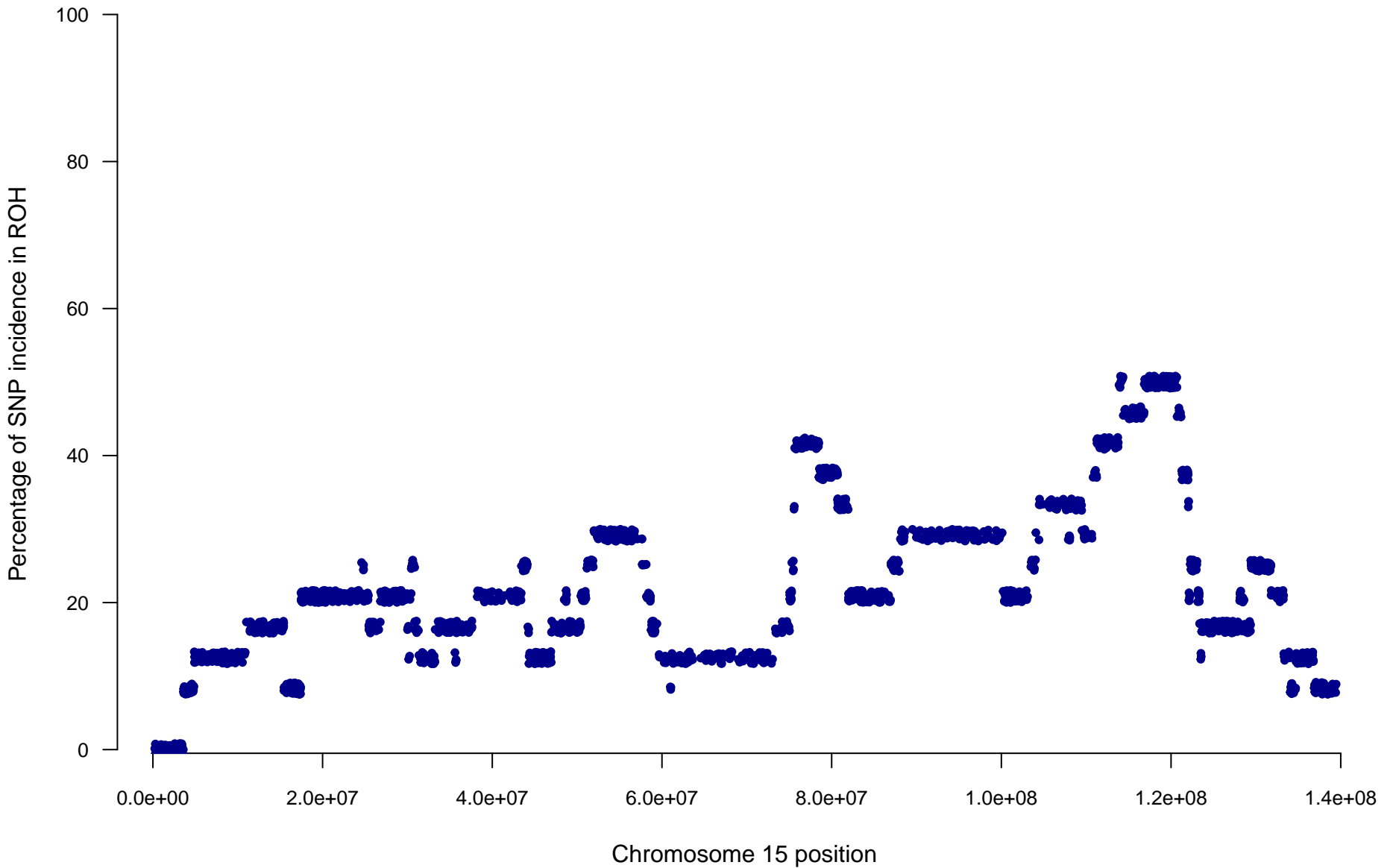

RM  
N= 24

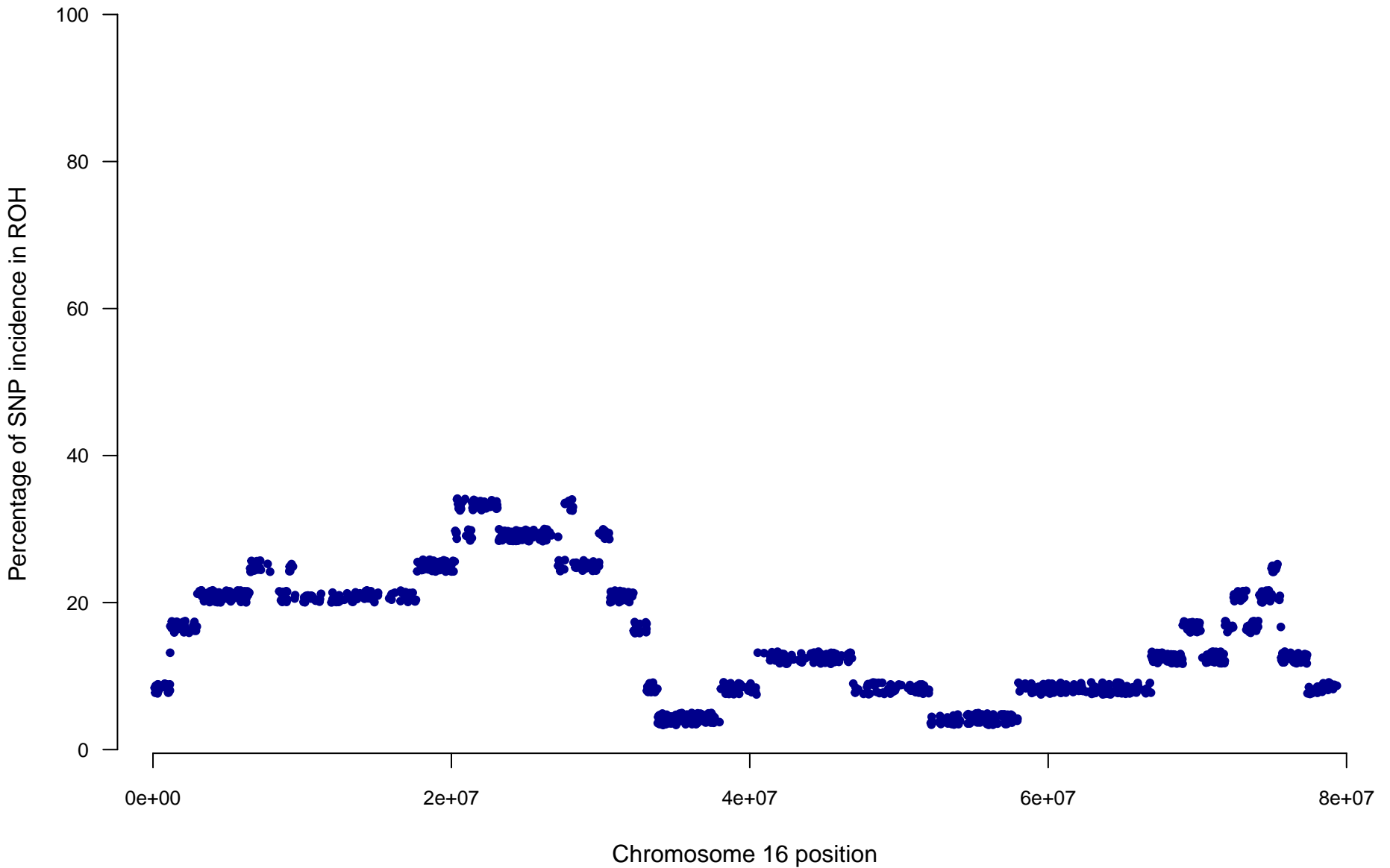

RM  
N= 24

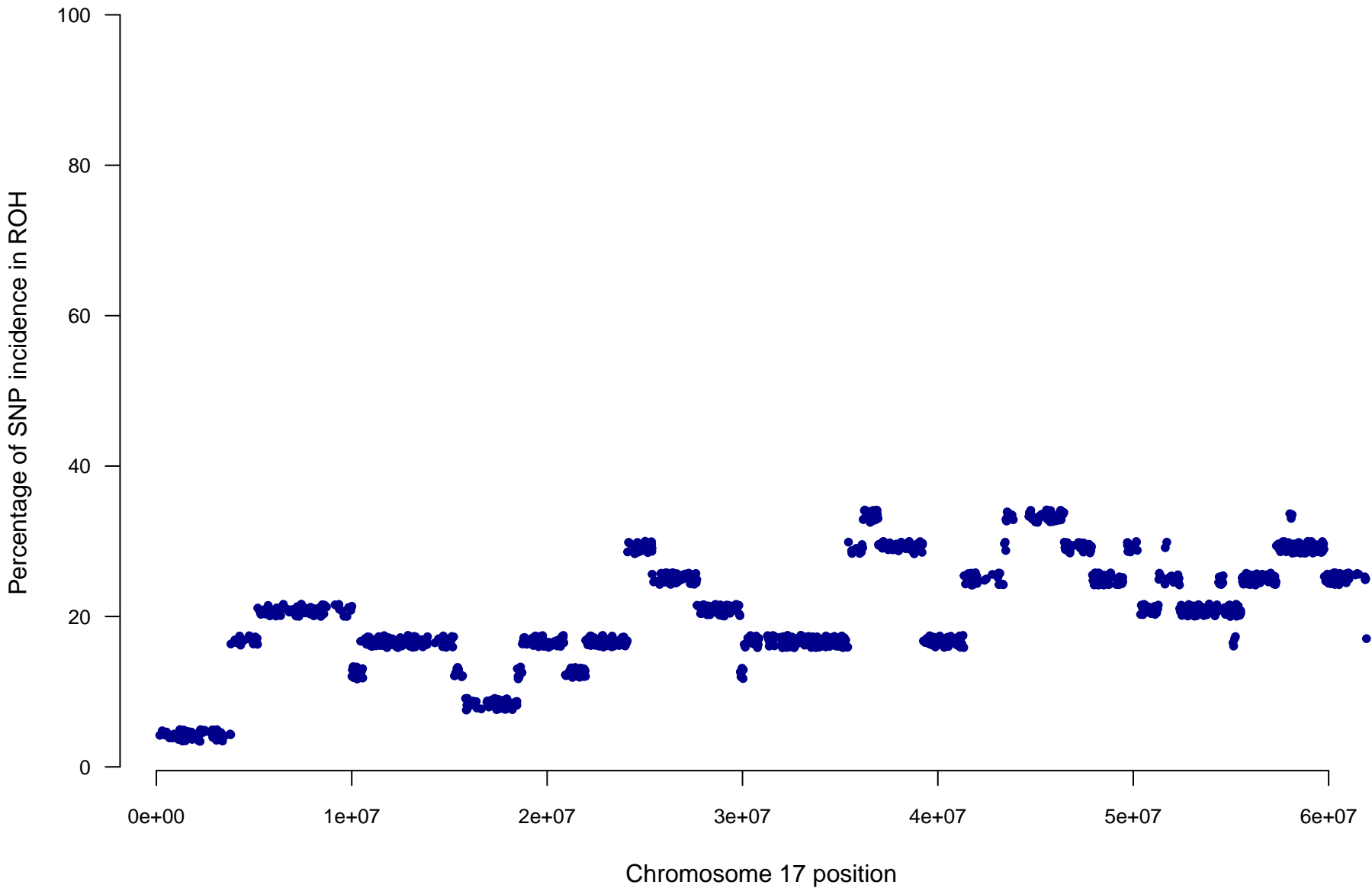

RM  
N= 24

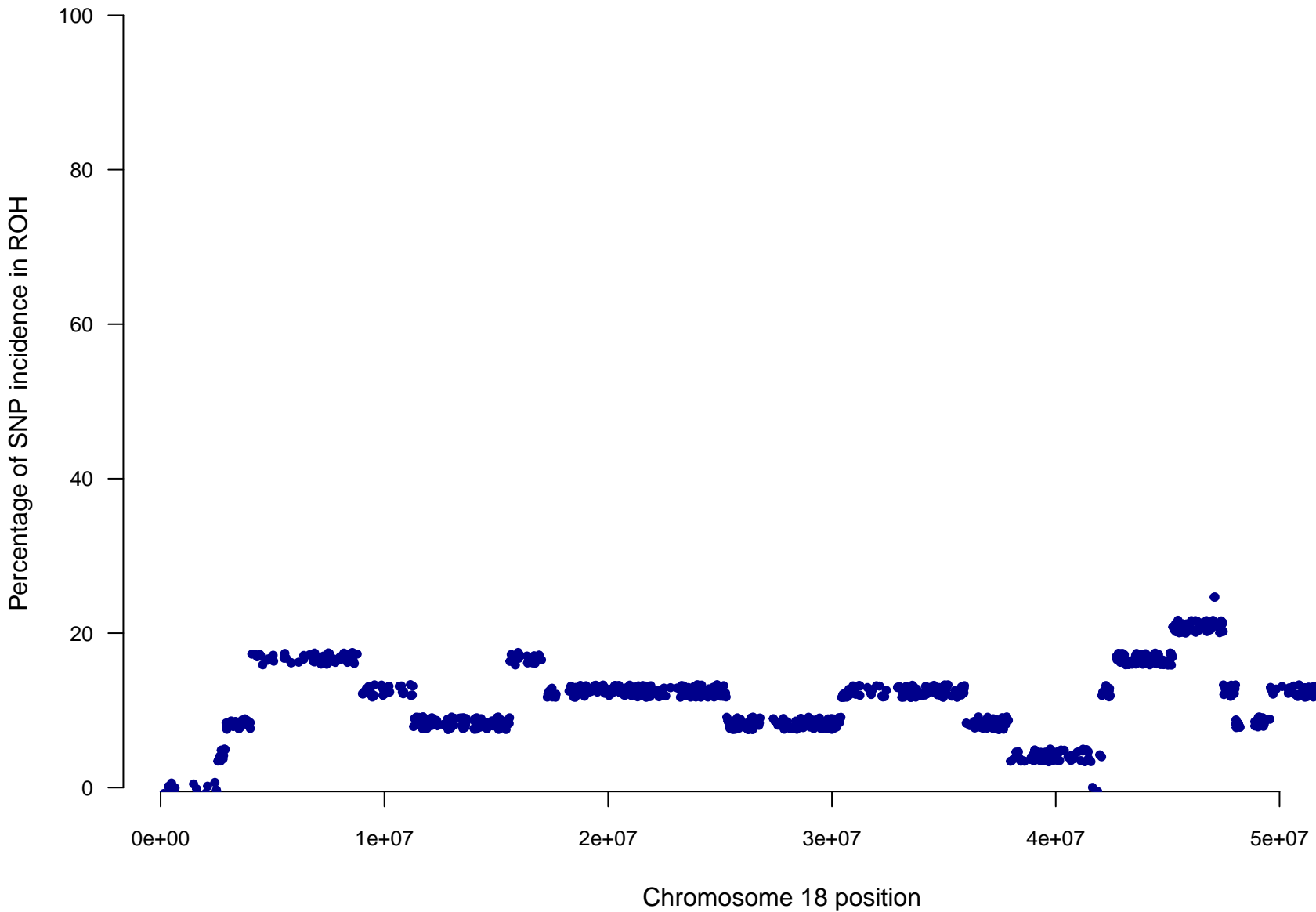

Supplement: Supplementary file 6 [file Image3.pdf]
